# Cognitive Processing Therapy for Posttraumatic Stress Disorder in Japan: A Randomized Clinical Trial

**DOI:** 10.1001/jamanetworkopen.2024.58059

**Published:** 2025-02-05

**Authors:** Masaya Ito, Akiko Katayanagi, Mitsuhiro Miyamae, Tamae Inomata, Yuriko Takagishi, Akiko Kikuchi, Miyuki Makino, Yoko Matsuda, Keiko Yamaguchi, Chiaki Nakayama, Kyosuke Kaneko, Chika Yokoyama, Fumi Imamura, Ayako Kanie, Mari Oba, Satoshi Tanaka, Satomi Nakajima, Tomomi Narisawa, Kyoko Akutsu, Rieko Konno, Yuki Oe, Naotsugu Hirabayashi, Toshi A. Furukawa, Patricia A. Resick, Masaru Horikoshi

**Affiliations:** 1 National Center for Cognitive Behavior Therapy and Research, National Center of Neurology and Psychiatry, Tokyo, Japan; 2 Cognitive Behavioral Therapy and Research Institute, Musashino University, Tokyo, Japan; 3 School of Arts and Sciences, Tokyo Woman’s Christian University, Tokyo, Japan; 4 National Center Hospital, National Center of Neurology and Psychiatry, Tokyo, Japan; 5 Division of Clinical Research & Education Promotion, Department of Clinical Data Science, National Center of Neurology and Psychiatry, Tokyo, Japan; 6 Department of Neuropsychiatry, Kyorin University School of Medicine, Tokyo, Japan; 7 Office of Institutional Advancement and Communications, Kyoto University, Kyoto, Japan; 8 Department of Psychiatry and Behavioral Sciences, Duke Health, Durham, North Carolina

## Abstract

**Importance:**

Cognitive processing therapy (CPT) is an evidence-based treatment for posttraumatic stress disorder (PTSD). However, there is little evidence on the efficacy of CPT in East Asia.

**Objective:**

To evaluate whether CPT is effective in treating PTSD among outpatients in a Japanese medical setting.

**Design, Setting, and Participants:**

This randomized clinical trial used a 16-week, single-center, assessor-blinded, parallel-group superiority design to examine the efficacy of CPT in conjunction with treatment as usual (CPT-TAU) vs waiting list with TAU (WL-TAU) from April 2016 through December 2022. The trial included adult patients with PTSD at a national psychiatric referral hospital in Tokyo, Japan. Analysis was based on intention to treat and per protocol and was performed from February 1 to April 30, 2024.

**Interventions:**

Participants were randomized 1:1 to CPT-TAU (n = 29), which consisted of 12 weekly individual CPT sessions, or WL-TAU (n = 31), which consisted of clinical monitoring and/or pharmacotherapy.

**Main Outcomes and Measures:**

The primary outcome was the Clinician-Administered PTSD Scale (CAPS-5) score for the *Diagnostic and Statistical Manual of Mental Disorders* (Fifth Edition) at 17 weeks. Secondary outcomes included self-reported PTSD symptoms assessed by the PTSD Checklist–5 and responder status at 17 weeks. Adverse events were evaluated using the Japanese version of the Common Terminology Criteria for Adverse Events, version 4.0.

**Results:**

Among 60 eligible participants (all included in the intention-to-treat analysis), mean (SD) age was 36.9 (9.9) years; 54 (90.0%) were women. The CPT-TAU group showed a mean (SE) reduction in CAPS-5 scores of 14.00 (1.92) points, with a low dropout rate (2 of 29 [6.9%]). Patients in the CPT-TAU group showed superiority in all secondary and other outcomes. The mean change difference was observed in depression (8.83; 95% CI, 6.00-11.66), suicidal ideation (6.73; 95% CI, 1.25-12.22), disability (8.16; 95% CI, 3.90-12.43), clinical global impression (0.84; 95% CI, 0.41-1.26), and loss of principal PTSD diagnosis (59.09; 95% CI, 37.19-81.00). There were no serious adverse events in the CPT-TAU group and 3 serious adverse events in the WL-TAU group during the intervention period.

**Conclusions and Relevance:**

In this randomized clinical trial of CPT-TAU vs WL-TAU, CPT was superior in reducing PTSD symptoms. These results strengthen the evidence for use of CPT in East Asian populations.

**Trial Registration:**

Umin.Uc.Jp/Ctr Identifier: UMIN000021670

## Key Points

**Question:**

Is cognitive processing therapy (CPT) combined with treatment as usual (TAU) superior to TAU alone among Japanese outpatients with posttraumatic stress disorder (PTSD)?

**Findings:**

In this randomized clinical trial that included 60 women and men, CPT with TAU was superior to TAU alone; 7% of participants dropped out of CPT.

**Meaning:**

These findings suggest that CPT should be considered for patients with PTSD in the Japanese clinical setting.

## Teaser

This randomized clinical trial investigates the safety and efficacy of cognitive processing therapy for posttraumatic stress disorder in a Japanese population.

## Introduction

Posttraumatic stress disorder (PTSD) is a persistent and debilitating mental disorder characterized by intrusion symptoms associated with a traumatic event, avoidance of triggering external and internal stimuli, alterations in cognition and mood, and hyperarousal or reactivity.^1^ Epidemiological studies reported a 12-month PTSD prevalence of 0.2% to 2.5% (Japan, 0.4%; US, 2.5%),^2^ with a global lifetime prevalence of 3.9% reported in 24 countries by the World Mental Health Surveys.^3^

Treatment guidelines^4,5,6,7^ and a systematic review^8^ have consistently reported that trauma-focused types of cognitive behavioral therapy (CBT) are the first-line treatment of choice for individuals with PTSD. Specifically, cognitive processing therapy (CPT) has been tested in 44 randomized clinical trials (RCTs) thus far^9^ using various formats (individual vs group, in-person vs telehealth) and versions (eg, CPT plus A [with written accounts] vs CPT without written account) among populations with diverse traumatic events.^10,11^ However, most previous studies have been limited to veterans or active military contexts. Although 4 RCTs have examined the efficacy of CPT outside Western cultural settings (ie, Egypt,^12^ Iraq,^13,14^ and the Democratic Republic of Congo^15^), to our knowledge, no RCTs have been reported in East Asia.

The present study aimed to investigate the safety and efficacy of CPT for PTSD in a Japanese population. The primary hypothesis was that the addition of CPT to TAU would result in superior outcomes compared with TAU alone regarding the severity of PTSD symptoms in patients with PTSD diagnoses, as assessed by the Clinician-Administered PTSD Scale (CAPS-5) for the *Diagnostic and Statistical Manual of Mental Disorders* (Fifth Edition) (*DSM-5*).^16^ The secondary objective was to test the superiority of CPT plus TAU vs TAU alone for self-reported PTSD symptoms, as assessed by patients’ responses on the PTSD Checklist for *DSM-5* (PCL-5) and the responder status and loss of PTSD diagnosis using CAPS-5. We also evaluated the safety of CPT for this patient population.

## Methods

### Design

We designed this study as a 16-week, single-center, assessor-blinded, parallel-group superiority RCT to compare the efficacy of CPT in conjunction with TAU vs TAU alone while wait-listed for CPT (WL-TAU) after the intervention period. The trial was conducted from April 2016 through December 2022. Minimization was used to balance the stratification factor of a traumatic event (single vs multiple or persistent) using a ratio of 1:1. The National Center of Neurology and Psychiatry institutional review board reviewed and approved the trial protocol (Supplement 1). All participants provided written informed consent. We also described the detailed methods in a published study protocol.^17^ Our report followed the Consolidated Standards of Reporting Trials (CONSORT) reporting guideline.

### Participants

Inclusion criteria consisted of (1) diagnosis of PTSD according to criteria outlined in the *DSM-5* as assessed by CAPS-5, (2) age between 18 and 70 years at baseline, and (3) provision of written informed consent before participation in the study. Exclusion criteria consisted of (1) severe substance use disorders at baseline as assessed by the Mini-International Neuropsychiatric Interview, version 7.0.0 (MINI),^18^ (2) current manic episode or psychotic disorders at baseline as assessed by the MINI, (3) severe suicidal ideation at baseline as assessed by the MINI, (4) severe or unstable physical disorders or major cognitive deficits at baseline, (5) involvement in manualized psychotherapy at baseline, and (6) other relevant reasons for exclusion as determined by the investigators.

### Interventions

#### CPT

The intervention group received in-person weekly CPT without a trauma account following the comprehensive manual.^11^ Although CPT usually consists of 12 sessions, a maximum of 16 sessions was allowed to approximate the timelines commonly used in Japanese medical settings for CBT.^19^ We considered completer status as the completion of all 12 sessions of CPT. The 7 psychotherapists who provided CPT were certified public psychologists with at least 14 hours of CPT training (M.I., A. Katayanagi, M. Miyamae, T.I., Y.T., A. Kikuchi, and M.H.). The supervisors (2 certified psychologists [M.I. and M.H.] and 1 physician [A. Kanie]) participated in more than 48 hours of training for CPT consultation. The CPT-trained clinical psychologists who did not serve as staff therapists (T.I. and S.T.) rated one-fifth of the sessions in each case using the Cognitive Processing Therapy: Therapist Adherence and Competence Protocol, Individual Version–Revised.^20^ Before starting this trial, we randomly sampled these sessions, and the staff therapist did not know which sessions would be assessed.

#### TAU

The Japanese Society for Traumatic Stress Studies published guidelines for the pharmacological management of PTSD.^21^ Because of the significant lack of clinicians trained in CBT and any systematic psychotherapy, the recommended first-line treatment involves the prescription of either paroxetine or sertraline, both of which are classified as selective serotonin reuptake inhibitors. In the present study, we considered pharmacotherapy, clinical monitoring, psychoeducation, and supportive counseling, commonly used for treating patients with PTSD in Japanese medical settings, as TAU. The psychiatrists who provided TAU referred all participants.

### Outcomes

The CAPS-5 was used to assess the primary outcome measure of PTSD symptoms at 17 weeks, whereas the PCL-5 and determination of responder status were used to assess the secondary outcomes at 17 weeks.^16^ Because there was no consensus operational definition of responder status,^22,23^ we used the following criteria to determine responder status: (1) clinical global impression improvement rating of 2 or less as assessed by item 28 of the CAPS-5 and (2) loss of PTSD diagnosis as assessed by CAPS-5. CAPS-5 scores range from 0 to 80, with higher scores indicating more severe PTSD symptoms.

We used PCL-5 to assess the severity of PTSD symptoms.^24,25^ For the other aspects of clinical outcomes, we used the Patient Health Questionnaire–9 (PHQ-9) to assess depression,^26^ the Suicidal Ideation Attributes Scale (SIDAS) to assess suicidality,^27^ the Euro-Qol 5-Dimension 5-Level (EQ-5D-5L) questionnaire to assess quality of life,^28^ the Sheehan Disability Scale (SDS) to assess functional impairment,^29^ and the Clinical Global Impression Severity and Improvement (CGI-S and CGI-I) in CAPS-5 to assess the clinical global impression. The time points for assessment with these secondary measures (except for loss of diagnosis) were 8, 17, and 34 weeks. We cautiously evaluated adverse events using the Japanese version of the Common Terminology Criteria for Adverse Events, version 4.0.^30^ The independent evaluators actively solicited patients at each visit to assess any signs of the occurrence or exacerbation of adverse symptoms and their severity, duration, and relation to the study. Serious adverse events included death, life-threatening conditions, hospital admission, hospitalization prolongation, disability, permanent damage, and congenital anomaly or disability.

### Statistical Analysis

We provided descriptive statistics to understand the participant characteristics. We examined the intraclass correlations to assess the interrater reliability used for the adherence and competence ratings for CPT sessions and ratings of CAPS-5. We calculated the Bang Index at the 8-, 17-, and 34-week assessments to examine the blinding success.^31^ If the Bang Index value was between −2.0 and 2.0 and its 1-sided 95% CI did not cross 0, the blinding was considered successful.

Following the intention-to-treat principle, we used data from all allocated participants to examine the safety and efficacy of the interventions. The linear mixed-effects model (LMM) analysis for the primary outcome used autoregressive repeated covariance type. Patient scores on CAPS-5 were the dependent variables. Group allocation (intervention, 0; control, 1), assessment time (baseline, 0; week 8, 1; and week 17, 2), and the interaction between group allocation and assessment time were fixed-effect factors. The participant was a random-effect factor. The level of statistical significance was set at *P* < .05 for 2-tailed *t* tests. We conducted similar LMM analyses for the secondary outcomes (eg, PCL-5) and other exploratory outcomes (PHQ-9, EQ-5D-5L, SIDAS, and SDS). All secondary analyses were exploratory. We examined a risk ratio with a 95% CI to evaluate the proportion of individuals who were considered to be responsive to treatment. We conducted data analysis using R, version 4.0.2 (R Program for Statistical Computing) and SAS, version 9.4 (SAS Institute Inc). For the per protocol analysis, we examined all data that did not meet the discontinuation criteria. We assessed maintenance and continuing improvement by calculating the within-effect size from posttreatment assessment (17 weeks) to follow-up (34 weeks). Additionally, we calculated the occurrence of adverse events during the intervention and follow-up period. We conducted sensitivity analyses controlling the types of traumatic events (single vs multiple). Data were analyzed from February 1 to April 30, 2024. The details of the sample size calculation are presented in the eMethods in Supplement 2.

## Results

### Description of Participants

**Figure 1.**
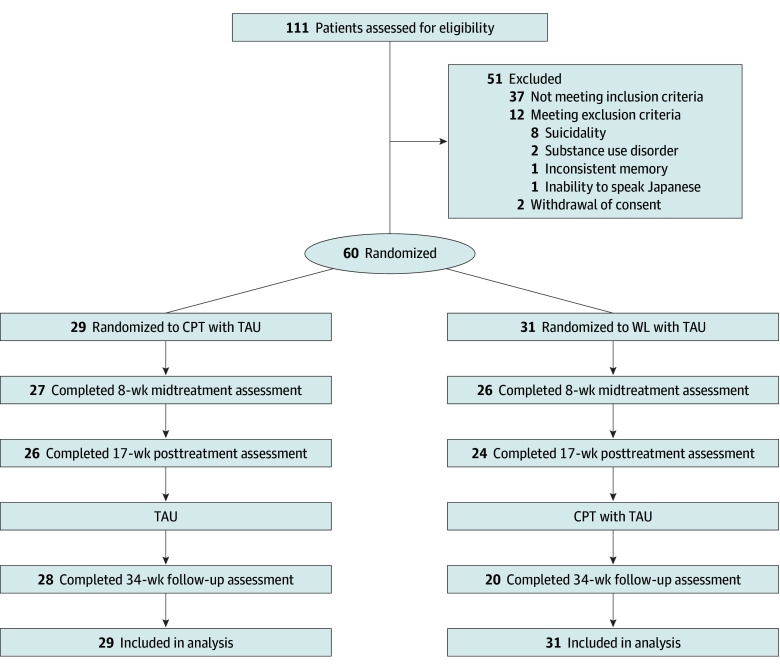
Study Participant Flow Diagram CPT indicates cognitive processing therapy; TAU, treatment as usual; and WL, waiting list.

We assessed 111 participants for eligibility between April 2016 and March 2022 (Figure 1). After exclusions, 60 participants were included in the intention-to-treat analysis. Mean (SD) age was 36.9 (9.9) years; 54 (90.0%) were women, and 6 (10.0%) were male. Of the 60 participants, 29 were randomized to CPT-TAU and 31 to WL-TAU. Table 1 shows the participants’ demographic and clinical characteristics at baseline. eTable 1 in Supplement 2 shows the types of index traumatic events, time with PTSD, time since index events, and reported history of childhood abuse. The most common index events were sexual assault (17 [28.3%]), other unwanted or uncomfortable sexual experiences (8 [13.3%]), and physical assault (17 [28.3%]). The mean (SD) duration of PTSD was 147.0 (126.2) months. Most patients had psychiatric comorbidity (38 [63.3%]) and suicidality (40 [66.7%]), used psychotropic medicine (43 [71.7%]), and had a history of abuse in childhood (27 [45.0%]).

**Table 1.** Baseline Characteristics

Characteristic	Patients^a^		
	All (N = 60)	CPT-TAU (n = 29)	WL-TAU (n = 31)
Age, mean (SD), y	36.9 (9.9)	37.2 (10.4)	36.9 (9.3)
Sex			
Female	54 (90.0)	27 (93.1)	27 (87.1)
Male	6 (10.0)	2 (6.9)	4 (12.9)
Marital status			
Single	28 (46.7)	13 (44.8)	15 (48.4)
Married	18 (30.0)	7 (24.1)	11 (35.5)
Divorced	10 (16.7)	7 (24.1)	3 (9.7)
Widowed	1 (1.7)	0	1 (3.2)
Married and living apart	3 (5.0)	2 (6.9)	1 (3.2)
Current occupation			
Employment	12 (20.0)	5 (17.2)	7 (22.6)
Student	6 (10.0)	3 (10.3)	3 (9.7)
Medical leave from job	7 (11.7)	3 (10.3)	4 (12.9)
Homemaker	9 (15.0)	3 (10.3)	6 (19.4)
Part-time job	6 (10.0)	2 (6.9)	4 (12.9)
Unemployed	19 (31.7)	13 (44.8)	6 (19.4)
Other	1 (1.7)	0	1 (3.2)
Educational level			
Junior high school	4 (6.7)	3 (10.3)	1 (3.2)
High school	17 (28.3)	11 (37.9)	6 (19.4)
Professional school	8 (13.3)	2 (6.9)	6 (19.4)
2-y College	7 (11.7)	4 (13.8)	3 (9.7)
University or college	21 (35.0)	8 (27.6)	13 (41.9)
Graduate school	2 (3.3)	1 (3.4)	1 (3.2)
Other	1 (1.7)	0	1 (3.2)
Comorbid diagnoses			
Depressive disorder	24 (40.0)	10 (34.5)	14 (45.2)
Anxiety disorder			
Panic disorder	5 (8.3)	1 (3.4)	4 (12.9)
Social anxiety disorder	7 (11.7)	2 (6.9)	5 (16.1)
Agoraphobia	14 (23.3)	6 (20.7)	8 (25.8)
Obsessive-compulsive disorder	2 (3.3)	1 (3.4)	1 (3.2)
Bipolar I disorder	1 (1.7)	0	1 (3.2)
Eating disorder			
Bulimia nervosa	2 (3.3)	1 (3.4)	1 (3.2)
Binge-eating disorder	4 (6.7)	2 (6.9)	2 (6.5)
Alcohol use disorder	2 (3.3)	2 (6.9)	0
Any of the above disorders	38 (63.3)	17 (58.6)	21 (67.7)
Suicidality			
Any	40 (66.7)	19 (65.5)	21 (67.7)
Low	19 (31.7)	6 (20.7)	13 (41.9)
Moderate	21 (35.0)	13 (44.8)	8 (25.8)
Suicidal behavior disorder			
Any	28 (46.7)	16 (55.2)	12 (38.7)
Current	5 (8.3)	4 (13.8)	1 (3.2)
In early remission	1 (1.7)	1 (3.4)	0
In remission	22 (36.7)	11 (37.9)	11 (35.5)
Psychotropic medication			
SSRI	15 (25.0)	7 (24.1)	8 (25.8)
SNRI	9 (15.0)	4 (13.8)	5 (16.1)
Tricyclic antidepressant	0	0	0
Mood stabilizer	4 (6.7)	1 (3.4)	3 (9.7)
Benzodiazepine	25 (41.7)	12 (41.4)	13 (41.9)
Antipsychotics	16 (26.7)	7 (24.1)	9 (29.0)
Other	21 (35.0)	12 (41.4)	9 (29.0)
Any of the above medications	43 (71.7)	21 (72.4)	22 (71.0)
Time from first psychiatric visit, mean (SD), mo	77.87 (86.7)	76.31 (82.1)	79.32 (90.7)
Previously used medical institutions, mean (SD), No.	2.78 (2.3)	2.41 (1.8)	3.14 (2.6)
Previous psychiatric hospitalizations	17 (28.3)	8 (27.6)	9 (29.0)
No. of times psychotherapy was used in the past, mean (SD)	0.88 (1.7)	0.97 (2.2)	0.8 (0.9)
Habitual use of alcohol	22 (36.7)	11 (37.9)	11 (35.5)
Habitual use of tobacco	8 (13.3)	4 (13.8)	4 (12.9)

Abbreviations: CPT, cognitive processing therapy; SNRI, selective noradrenergic reuptake inhibitor; SSRI, selective serotonin reuptake inhibitor; TAU, treatment as usual; WL, waiting list.

^a^ Data are presented as number (percentage) of participants unless otherwise indicated.

### Attrition, Treatment Expectancy, and Treatment Adherence

Of 29 participants randomized to CPT-TAU, 27 (93.1%) completed the intervention, while 24 of 31 participants (77.4%) completed the WL-TAU condition. In the CPT-TAU group, 1 participant dropped out before starting CPT, and 1 dropped out after session 1 (dropout rate, 2 of 29 [6.9%]). The mean (SD) number of sessions for those who completed treatment was 13.19 (1.25) for the CPT-TAU group. The median treatment duration was 88.9 (IQR, 84.0-101.5) days. Eighteen participants (62.1%) terminated CPT at 17 weeks. The total number of sessions in the CPT-TAU group was 307 during the intervention period. Of these 307 sessions, the CPT-trained clinical psychologists who did not serve as staff therapists assessed 55 sessions for adherence and competence. The mean (SD) adherence for 252 item ratings was 90% (30% [range, 0%-100%]); the mean (SD) competence score for 222 item ratings was 4.22 (1.09 [range, 1.00-5.00]). Single-rater, 2-way, random-effects intraclass correlation coefficients (ICCs) estimated from 55 double-rated sessions were good for both adherence (ICC [2,1], 0.91) and competence (ICC [2,1], 0.66) scores.

### Assessment Integrity and Blinding Success for Random Allocation

To examine the interevaluator reliability of CAPS-5, we examined 38 of 196 randomly selected assessments (19.4%). The single-measure interrater ICC between the 2 evaluators was 0.99 (95% CI, 0.98-0.99) for the total CAPS-5 score. The mean ICC at the item level of CAPS-5 was 0.92 (range, 0.78-0.99). The blinding procedure was successful at 8 weeks but failed at 17 and 34 weeks, as described in the eResults in Supplement 2.

### Primary Outcome

**Figure 2.**
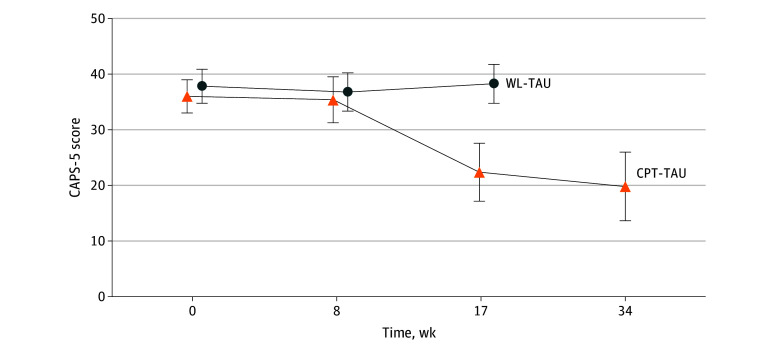
Posttraumatic Stress Disorder (PTSD) Severity From Baseline to Week 34 PTSD severity was measured using the Clinician-Administered PTSD Scale (CAPS-5) for the *Diagnostic and Statistical Manual of Mental Disorders* (Fifth Edition). CAPS-5 scores range from 0 to 80, with higher scores indicating more severe PTSD symptoms. Error bars represent 95% CIs. CPT indicates cognitive processing therapy; TAU, treatment as usual; and WL, waiting list.

Mean (SD) CAPS-5 scores in the CPT-TAU and WL-TAU groups were 35.97 (7.82) and 37.81 (8.23) at baseline, respectively, and 22.36 (11.76) and 38.23 (7.89) at 17 weeks, respectively (Table 2). As shown in Table 3, LMM analysis showed a significant difference in the CAPS-5 score at the primary time point of 17 weeks between the CPT-TAU and WL-TAU groups (mean change difference, 13.85 points; 95% CI, 8.46-19.24 points). The mean (SE) estimated changes from baseline to 17 weeks were 14.00 (1.92) for the CPT-TAU group and 0.15 (1.91) for the WL-TAU group (Figure 2).

**Table 2.** Descriptive Statistics of Outcomes

Variable	Baseline		Midassessment (8 wk)		Posttreatment assessment (17 wk)		Follow-up (34 wk)^a^	
	CPT-TAU (n = 29)	WL-TAU (n = 31)	CPT-TAU (n = 24)	WL-TAU (n = 25)	CPT-TAU (n = 22)	WL-TAU (n = 22)	CPT-TAU (n = 24)	WL-TAU (n = 19)
Score, mean (SD)								
CAPS-5^b^	35.97 (7.82)	37.81 (8.23)	35.38 (9.78)	36.76 (8.30)	22.36 (11.76)	38.23 (7.89)	19.79 (14.61)	27.26 (11.60)
PCL-5^b^	49.39 (14.61)	48.84 (11.17)	43.70 (16.04)	47.64 (12.18)	26.52 (20.88)	51.46 (9.75)	22.88 (18.95)	26.05 (17.81)
PHQ-9^c^	17.14 (6.17)	16.39 (5.16)	14.85 (6.68)	17.00 (6.27)	9.27 (6.79)	18.12 (4.90)	9.00 (7.12)	11.35 (6.86)
SIDAS^d^	7.59 (8.62)	8.03 (8.62)	4.96 (6.15)	9.58 (14.06)	2.73 (3.67)	10.29 (11.10)	3.44 (7.02)	6.00 (10.34)
EQ-5D-5L^e^	0.63 (0.20)	0.60 (0.13)	0.64 (0.13)	0.59 (0.12)	0.75 (0.16)	0.55 (0.16)	0.76 (0.17)	0.62 (0.20)
SDS^f^	17.52 (6.58)	18.58 (6.67)	17.04 (6.24)	20.65 (6.63)	13.15 (8.86)	22.67 (6.04)	9.26 (8.77)	13.45 (7.11)
CGI-S^g^	2.59 (0.50)	2.58 (0.50)	2.54 (0.51)	2.40 (0.76)	1.73 (0.88)	2.59 (0.59)	1.58 (0.93)	2.16 (0.90)
CGI-I^h^	NA	NA	3.67 (0.64)	3.52 (0.71)	2.41 (1.18)	3.68 (0.65)	1.96 (1.04)	2.47 (0.84)
Responder status, No. (%)^i^	NA	NA	2 (6.9)	3 (9.7)	13 (44.8)	2 (6.5)	17 (58.6)	10 (32.3)
Loss of PTSD diagnosis, No. (%)^i^	NA	NA	4 (13.8)	4 (12.9)	14 (48.3)	1 (3.2)	14 (48.3)	9 (29.0)

Abbreviations: CAPS-5, Clinician-Administered PTSD Scale for the *Diagnostic and Statistical Manual of Mental Disorders* (Fifth Edition); CGI-I, Clinical Global Impression–Improvement; CGI-S, Clinical Global Impression–Severity; CPT, cognitive processing therapy; EQ-5D-5L, Euro-QoL 5-Dimension 5-Level; PCL-5, Posttraumatic Stress Disorder Checklist for the *Diagnostic and Statistical Manual of Mental Disorders* (Fifth Edition); PHQ-9, Patient Health Questionnaire–9; PTSD, posttraumatic stress disorder; SDS, Sheehan Disability Scale; SIDAS, Suicidal Ideation Attributes Scale; TAU, treatment as usual; WL, waiting list.

^a^ Participants allocated to WL-TAU received CPT during the follow-up period.

^b^ Scores range from 0 to 80, with higher scores indicating greater severity of PTSD.

^c^ Scores range from 0 to 27, with higher scores indicating greater severity of depression.

^d^ Scores range from 0 to 50, with higher scores indicating more suicidal ideation.

^e^ Scores range from −0.025 to 0.895, with higher scores indicating higher health-related quality of life.

^f^ Scores range from 0 to 30, with higher scores indicating greater impairment.

^g^ Scores range from 0 to 7, with higher scores indicating greater severity.

^h^ Scores range from 0 to 7, with higher scores indicating worse illness.

^i^ Proportions were calculated using the baseline number as the denominator.

**Table 3.** Primary and Secondary Outcomes

Measure	Mean change from baseline to 17 wk (SE)		Mean change difference between WL-TAU and CPT-TAU (95% CI)
	CPT-TAU	WL-TAU	
Primary outcome			
CAPS-5 score	14.00 (1.92)	0.15 (1.91)	13.85 (8.46 to 19.24)
Secondary outcomes			
PCL-5 score	22.96 (3.20)	−2.22 (3.18)	25.18 (16.24 to 34.13)
PHQ-9 score	7.69 (1.00)	−1.14 (1.02)	8.83 (6.00 to 11.66)
SIDAS score	4.79 (1.94)	−1.95 (1.97)	6.73 (1.25 to 12.22)
EQ-5D-5L score	−0.11 (0.03)	0.03 (0.03)	−0.15 (−0.22 to −0.08)
SDS score	4.39 (1.51)	−3.77 (1.53)	8.16 (3.90 to 12.43)
CGI-S score	0.83 (0.15)	0.00 (0.15)	0.84 (0.41 to 1.26)
CGI-I score	NA	NA	−1.26 (−1.76 to −0.76)

Abbreviations: CAPS-5, Clinician-Administered PTSD Scale for the *Diagnostic and Statistical Manual of Mental Disorders* (Fifth Edition); CGI-I, Clinical Global Impression–Improvement; CGI-S, Clinical Global Impression–Severity; CPT, cognitive processing therapy; EQ-5D-5L, Euro-QoL 5-Dimension 5-Level; NA, not applicable; PCL-5, Posttraumatic Stress Disorder Checklist for the *Diagnostic and Statistical Manual of Mental Disorders* (Fifth Edition); PHQ-9, Patient Health Questionnaire–9; SDS, Sheehan Disability Scale; SIDAS, Suicidal Ideation Attributes Scale; TAU, treatment as usual; WL, waiting list.

### Secondary Outcomes and Sensitivity Analyses

For the dichotomous outcomes, 8 of 29 patients in the CPT-TAU group (44.8%) and 2 of 31 patients in the WL-TAU group (6.5%) exhibited a treatment response at 17 weeks, resulting in an incidence proportion ratio of 6.50 (95% CI, 1.66–25.49). A loss of the PTSD diagnosis was observed in 14 patients (48.3%) in the CPT-TAU group and in 1 (3.2%) in the WL-TAU group, resulting in an incidence proportion ratio of 14.00 (95% CI, 2.01-97.50). The details of the analyses are presented in eTable 2 in Supplement 2. From baseline to 17 weeks, the CPT-TAU group had a decrease in suicidal ideation (mean [SD] SIDAS score, 7.59 [8.62] vs 2.73 [3.67]), while the WL-TAU group showed an increase (mean [SD] SIDAS score, 8.03 [8.62] vs 10.29 [11.10]). As shown in Table 3, the LMM analyses showed differences in changes of all continuous measures for secondary outcomes. Patients in the CPT-TAU group showed superiority in all secondary and other outcomes. The mean change difference was observed in depression (8.83; 95% CI, 6.00-11.66), suicidal ideation (6.73; 95% CI, 1.25-12.22), disability (8.16; 95% CI, 3.90-12.43), clinical global impression (0.84; 95% CI, 0.41-1.26), and loss of principal PTSD diagnosis (59.09; 95% CI, 37.19-81.00). The LMM analyses that included stratified variables (ie, single traumatic event vs multiple or serial traumatic events) as covariates showed significant differences between the CPT-TAU and WL-TAU groups regarding the changes in all outcome measures at the primary time point of 17 weeks (eTable 3 in Supplement 2). The LMM analyses using per protocol samples revealed consistent results, as significant differences were observed between the CPT-TAU and WL-TAU groups regarding the changes in all outcome measures (eTable 4 in Supplement 2).

### Maintenance of Treatment Effect During the Follow-Up Period

The changes in each outcome from posttreatment to follow-up for the CPT-TAU group were 2.88 (95% CI, −1.47 to 7.24) for CAPS-5 score; 4.62 (95% CI, −1.99 to 11.23) for PCL-5 score; 0.42 (95% CI, −1.94 to 2.79) for PHQ-9 score; −0.41 (95% CI, −4.39 to 3.56) for SIDAS score; −0.02 (95% CI, −0.07 to 0.04) for EQ-5D-5L score; and 0.17 (95% CI, −0.19 to 0.52) for the Clinical Global Impression–Severity score (eTable 5 in Supplement 2). The 95% CI crossed 0 for all of these variables, suggesting the maintenance of the treatment effect. The significant within-group effect size in SDS (Hedges *g,* 4.05; 95% CI, 0.99-7.11) and Clinical Global Impression–Improvement (Hedges *g,* 0.46; 95% CI, 0.03-0.89) showed further improvement in patients in the CPT-TAU group.

### Adverse Events

The independent evaluators’ assessment of the occurrence of adverse events is shown in eTable 6 in Supplement 2. We observed no serious adverse events in the CPT-TAU group and 3 serious adverse events in the WL-TAU group during the intervention period. During the intervention period, 17 of 29 participants in the CPT-TAU group (58.6%) reported 62 adverse events. Twenty-three of 31 participants in the WL-TAU group (74.2%) reported 75 adverse events.

## Discussion

The present study is the first RCT, to our knowledge, to test the efficacy of CPT in East Asia. As hypothesized, adding CPT to TAU was more effective than waiting for CPT while receiving TAU in reducing the severity of PTSD symptoms from baseline to 17 weeks. The CPT-TAU group showed significant improvements compared with the WL-TAU group in the blinded assessor evaluation of severity and improvement of clinical global impression, responder status, and loss of PTSD diagnosis. We observed the superiority of CPT-TAU over WL-TAU in self-report measures of PTSD symptoms, depression, suicidal ideation, quality of life, and functioning. The CPT-TAU group maintained improvements in all outcomes from 17 to 34 weeks. Sensitivity analyses controlling for the types of traumatic events (single vs multiple) and per protocol analyses supported the robustness of these results. The proportion of dropouts was low in the CPT-TAU group (2 of 29 [6.9%]). No serious adverse events occurred in the CPT-TAU group during the intervention period.

The results supported the superior efficacy of CPT with TAU over TAU alone in all primary and secondary outcomes. The mean (SE) reduction of PTSD symptoms assessed by CAPS-5 (14.00 [1.92]) was comparable to those in previous clinical trials that tested CPT in the US^32,33^ and Egypt.^12^ The decrease in depression was consistent with that in previous reports.^33,34,35,36^ Given that functioning and clinical global impression continued to improve until 34 weeks, functioning in daily life might improve later, after the earlier improvement in posttraumatic symptoms, depression, and suicidal ideation. These results are consistent with previous findings supporting the efficacy of CPT in terms of broader clinical features.^9^ Participants in our study showed some clinical conditions at baseline, such as a long duration of PTSD (mean number of months, 147.0), high levels of psychiatric comorbidity (63.3%) and suicidality (66.7%), use of psychotropic medicine (71.7%), and any history of abuse in childhood (45.0%). Notwithstanding these characteristics, CPT was found to be effective.

Our study is the first, to our knowledge, to report the *DSM-5* diagnosis outcome for CPT in Asia. Half of the participants in the CPT-TAU group lost the PTSD diagnosis (48.3%), while 1 participant in the WL-TAU group lost the diagnosis (3.2%). This is comparable to previous reports of CPT plus A (with written accounts) for achieving loss of diagnosis after treatment among treatment-seeking veterans with military-related PTSD (28%,^37^ 29%,^38^ 39%,^39^ and 40%^34^) and females with PTSD who had experienced rape (53%).^35^ Reports indicated that CPT resulted in a higher proportion of loss of diagnosis compared with other evidence-based treatments for PTSD.^40^ Our administration of CPT had a high proportion of adherence (90% of the 252 item ratings were adherent) and a high level of competence (mean competence score of 4.22, corresponding to “good skills, included all major elements of the session; some areas could be improved”^20^). Such appropriate delivery may have elicited the therapeutic effects of CPT.

We observed a low proportion of dropouts from CPT (6.9%). Although this finding is consistent with the rate in our pilot trial (1 of 25 [4.0%]),^36^ it differs from the results of previous CPT studies (14.9%-46.0%)^32,33,41,42^ and other types of evidence-based psychotherapies for PTSD (18%).^43^ This difference might be partially attributable to the clinical context or administration of CPT. In Japan, evidence-based treatment for PTSD is rare. To our knowledge, only 1 RCT of prolonged exposure therapy for PTSD has been reported.^44^ Such clinical circumstances might motivate participants to continue treatment.

The CPT-TAU group had fewer adverse events compared with the WL-TAU group. No serious adverse events occurred in the CPT-TAU group over 17 weeks, whereas 3 serious adverse events occurred in the WL-TAU group. Thus, CPT might prevent adverse events and serious adverse events. In addition, from baseline to 17 weeks, the CPT-TAU group exhibited a decrease in suicidal ideation (mean SIDAS score, 7.59 vs 2.73), while the WL-TAU group showed an increase (mean SIDAS score, 8.03 vs 10.29). These findings are notable because many clinicians assume that focusing on and discussing traumatic events is more distressing than other types of psychotherapy.^45^

### Strengths and Limitations

The strengths of our research include the rigorous design using blinded CAPS-5 assessment with high interrater reliability, administration of CPT with high adherence and competence, and cautious evaluation of adverse events, in contrast to previous trials, which did not report adverse events and their causes.^46^ Because the national health insurance scheme in Japan only covers prolonged exposure therapy for PTSD conducted by a trained psychiatrist or nurse and this inclusion of prolonged exposure therapy in 2016 had no impact on the increase in the total number of CBT sessions in Japan,^47^ the efficacy and safety results of CPT conducted by certified public psychologists in the present study are important in considering the dissemination of evidence-based treatments for PTSD in Japan.

This study also has limitations. First, we failed to blind the allocation from the independent evaluators at 17 and 34 weeks. The large treatment effect may have made the independent evaluators’ allocation assumptions more accurate. Such breaking of blinding is not considered to constitute a risk of bias in the Cochrane risk of bias assessment for assessment bias.^48^ Second, most of the participants were female. Third, we could not assess PTSD and complex PTSD diagnoses defined by the *International Classification of Diseases, 11th Revision*. Fourth, we did not use active control conditions, such as prolonged exposure or present-centered therapy. Fifth, we could not restrict changes to pharmacotherapy during this study because we considered that participant recruitment would be hindered by doing so in Japan.

## Conclusions

In this RCT of CPT-TAU vs WL-TAU, CPT was superior to WL in reducing PTSD symptoms. The current study is, to our knowledge, the largest RCT of individual psychotherapy for PTSD in Japan. These findings indicate that adding CPT to TAU may be efficacious for patients with PTSD in reducing posttraumatic symptoms, depression, and suicidality and improving quality of life and functioning.

## Notes

Supplement 1.
Trial Protocol

Supplement 2. **eMethods.** Sample Size Estimation **eTable 1.** Baseline Trauma Characteristics **eResults.** Blinding Success for Random Allocation **eTable 2.** Secondary Outcomes (Dichotomous Variables) **eTable 3.** Sensitivity Analyses Using a Stratified Variable as a Covariate **eTable 4.** Sensitivity Analyses Among the Per-Protocol Sample **eTable 5.** Differences Between the 17- and 34-Week Time Points in the CPT-TAU Group **eTable 6.** Summary of Independent Evaluator-Rated Adverse Events

Supplement 3.
Data Sharing Statement
