# Supplementary material for: Cognitive Processing Therapy for Posttraumatic Stress Disorder in Japan: A Randomized Clinical Trial
Source: JAMA Netw Open. 2025 Feb 5;8(2):e2458059. doi: 10.1001/jamanetworkopen.2024.58059 (PMC11800015; doi:10.1001/jamanetworkopen.2024.58059)
Supplement: Supplement 1. — Trial Protocol [file jamanetwopen-e2458059-s001.pdf]

研究計画書

Research Protocol

(※NCNP倫理審査承認2015−111から数えて)第8.6版：2024年4月12日
(Version 8.6, Approved by NCNP Ethics Review Board, 2024-04-12)

1. 研究の名称 Research Title

研究課題名 Research Topic	心的外傷後ストレス障害に対する認知処理療法の有効性に関するランダム化比較試験（**SPINET**）<br>Randomized Controlled Trial on the Effectiveness of Cognitive Processing Therapy for Post-Traumatic Stress Disorder (SPINET)
研究責任者（所属）Principal Investigator (Affiliation):	伊藤正哉 （認知行動療法センター）<br>Masaya Ito (National Center for Cognitive-Behavior Therapy and Research)

【提出に際してのチェックリスト】（提出前に必ずすべての項目を確認してください。）
Checklist for Submission (Please ensure all items are checked before submission)

以下の項目について、本研究計画に関する状況に当てはまる選択肢の前にある□を■に置き換えてください。
Please replace the □ before the options that apply to the situation of this research plan with ■.

1 臨床研究法で定義される「臨床研究」への該当性
研究目的は医薬品等※の有効性又は安全性、医療機器の性能評価である。 □ はい ■ いいえ
※「医薬品等」とは、薬機法上の医薬品・医療機器・再生医療等製品（体外診断用医薬品を除く）

1. Applicability under the Clinical Research Act: The research purpose is to evaluate the efficacy or safety of a pharmaceutical product or the performance of a medical device. □ Yes ■ No
"Medicines, etc." refers to pharmaceuticals, medical devices, and regenerative medicine products under the Pharmaceutical Affairs Law (excluding in vitro diagnostic drugs).

→「はい」の場合、臨床研究法で定める「臨床研究」に該当する可能性があります。
続いて、以下の項目を確認してください。

→ If the answer is "Yes," it may correspond to "Clinical Research" as defined by the Clinical Research Act.
Next, please review the following items.

1.1 未承認/適応外の医薬品等※を人に対して投与又は使用する予定である
□ はい □ いいえ
1.2 医薬品等製造販売業者から研究資金等の提供を受けて実施予定である
□ はい □ いいえ
※ いずれかで「はい」が選択される場合、特定臨床研究に該当する可能性があります。倫理委員会事務局または臨床研究相談窓口にご相談ください。

1.1 Unapproved/off-label pharmaceutical products will be administered or used on humans.

□ Yes □ No

1.2 1.2 Funding or other resources from a pharmaceutical product manufacturer will be provided for this study.

□ Yes □ No

If any of the above is answered "Yes," the study may be subject to specific clinical research regulations. Please consult the Ethics Committee Secretariat or the Clinical Research Consultation Office.

2 迅速審査への該当性
Eligibility for Expedited Review:

2.1 適用を受ける指針
■人を対象とする医学系研究に関する倫理指針
□その他（ ）

2.1 Applicable guidelines:
■ Ethical Guidelines for Medical and Health Research Involving Human Subjects
□ Others (__________________________)

2.2 他施設での審査実施の有無
多施設共同研究であって、既に当該研究の全体について主幹となる機関で倫理審査委員会の審査を受け、その実施について承認されている
■該当しない □実施について承認を得ている
→承認を得ている場合は、承認書の写しと提出した計画書等一式を添付すること

2.2 Approval Status from Other Institutions:

The study is a multicenter collaborative study and has already been reviewed and approved by the Ethics Committee of the primary institution.

■ Not Applicable　　　□ Approval has been obtained

→ If approved, please attach a copy of the approval document and the submitted protocol.

2.3　侵襲※の有無と程度
本研究計画の実施において、研究対象者に対して
□侵襲を伴わない　　■軽微な侵襲を伴う　　　　□侵襲を伴う（実施・安全体制確認書添付）
※「侵襲」とは、研究目的で行われる、穿刺、切開、薬物投与、放射線照射、心的外傷に触れる質問等によって、研究対象者の身体又は精神に傷害又は負担が生じることをいう。侵襲のうち、研究対象者の身体及び精神に生じる傷害及び負担が小さいものを「軽微な侵襲」という。詳細に関しては「人を対象とする医学系研究に関する倫理指針」ガイダンス P6 を参照。
→「侵襲を伴う」場合には、実施医療機関の管理者の「実施・安全体制確認書」が必須となるため、必要事項を記入し、倫理審査委員会の申請書と一緒に添付してください。

2.4　**Invasiveness and Degree:**

During the execution of this research plan, the following levels of invasiveness will be experienced by the research subjects: □ No invasiveness ■ Minor invasiveness □ Invasive (attach Implementation and Safety Confirmation Form)

"Invasion" refers to actions conducted for research purposes that cause harm or burden to the physical or mental well-being of research subjects, such as through punctures, incisions, drug administration, radiation exposure, or questions touching on psychological trauma. Among these, "minor invasions" are those that cause minimal harm or burden to the body and mind of the research subjects. For details, refer to the "Ethical Guidelines for Medical Research Involving Human Subjects," Guidance P6.

→ If the study involves "invasion," a "Confirmation of Implementation and Safety System" from the managing administrator of the implementing medical institution is required. Please complete the necessary information and attach it with the application form to the Ethics Committee.

2.5　介入※の有無
本研究計画の実施において、研究対象者に対して
□介入を行わない　　■介入を行う
※「介入」とは、研究目的で、人の健康に関する様々な事象に影響を与える要因（健康の保持増進につながる行動及び医療における傷病の予防、診断又は治療のための投薬、検査等を含む。）の有無又は程度を制御する行為（通常の診療を超える医療行為であって、研究目的で実施するものを含む。）をいう。詳細に関しては「人を対象とする医学系研究に関する倫理指針」ガイダンス P9 を参照。

2.4. Intervention:

During the execution of this research plan, the following intervention will be performed on the research subjects: □ No intervention ■ Intervention will be conducted

"Intervention" refers to actions that control the presence or extent of factors affecting various aspects of human health for research purposes (including behaviors that promote health and measures in medical settings such as prevention, diagnosis, or treatment of illness through medication, tests, etc.). This includes medical practices beyond routine clinical care that are conducted for research purposes. For details, refer to the "Ethical Guidelines for Medical Research Involving Human Subjects," Guidance P9.

2.6　他機関からの倫理審査依頼の有無

□審査依頼がある　　■審査依頼はない

→主たる研究機関が当センターであり、審査を依頼する機関に倫理委員会がない場合のみ審査依頼可能。倫理審査依頼書および申請書を添付してください。

2.5. Requests for Ethics Review from Other Institutions:

□ There is a review request ■ No review request

→ Review requests can only be made if the primary research institution is our center and the institution requesting the review does not have an ethics committee. Please attach the ethics review request form and the application form.

3　利益相反マネジメント状況

3　Conflict of Interest Management:

本研究計画書に記載する共同研究者のうち当センターに所属する全ての者（新たに追加された共同研究者も含む）に関して、本研究についてセンター利益相反マネジメント委員会に

■申告済み　　□申告の必要はない

→新規申請においては、必ず COI 申告をお願いします。

→倫理委員会への申請前（又は同時に）COI 申告を行ってください。倫理委員会での承認は COI 申告結果通知後となります。詳細は COI 委員会事務局（内線 2224、mail：coi-jimu@ncnp.go.jp）まで問い合わせ願います。

Regarding the co-researchers described in this research protocol, all members affiliated with this center (including newly added co-researchers) have:

■ Submitted their Conflict of Interest (COI) declaration □ No need to submit a COI declaration

→ For new applications, please be sure to submit a COI (Conflict of Interest) declaration.

→ Submit the COI declaration before (or simultaneously with) the application to the Ethics Committee. Approval by the Ethics Committee will occur after receiving the COI declaration results. For details, please contact the COI Committee Secretariat (Extension 2224, email: coi-jimu@ncnp.go.jp).

4　試料・情報の提供状況

当センターから他機関へ試料・情報の提供を　　□　行う　　　■　行わない

→資料・情報の提供を「行う」場合、必ず別添１：「試料・情報を提供する場合における記録事項表」を記載してください。

→他機関に試料・情報を提供（無償、有償に関わらず）する場合は、契約が必要となる可能性があります。他機関へ試料・情報を提供する場合は、BD 室（mail: tmcbd@ncnp.go.jp）に相談してください。

4. Provision of Samples/Information:

No provision of samples/information from this center to other institutions: □ Yes ■ No

→ If you provide materials or information, please be sure to fill out Appendix 1: "Record of Items to be Noted When Providing Samples or Information."

→ If you are providing samples or information to other institutions (whether free of charge or for a fee), a contract may be required. Please consult with the BD Office (email: tmcbd@ncnp.go.jp) when providing samples or information to other institutions.

5　契約締結の要否の確認状況

本研究の実施に際して、ビジネス・ディベロップメント室（BD 室）又は契約係に相談した結果

□契約書を締結する　　□契約書の締結は不要である　　■契約締結は不要のため相談していない

→他機関へ業務を委託する場合、他機関と共同研究もしくは委受託研究を行う場合に、契約が必要となる可能性があります。他機関へ業務を委託する場合は契約係に、他機関と共同研究もしくは委受託研究を行う場合には、BD 室（mail: tmcbd@ncnp.go.jp）に相談してください。

5　Confirmation Status of Contract Conclusion:

In the course of executing this research, consultation with the Business Development Office (BD Office) or the Contracts Section has resulted in:

□ A contract will be concluded □ No need to conclude a contract ■ No consultation required as contract conclusion is unnecessary

→ If you are outsourcing work to other institutions or conducting joint research or commissioned research with other institutions, a contract may be required. For outsourcing work to other institutions, please contact the Contract Department. For joint research or commissioned research with other institutions, please consult with the BD Office (email: tmcbd@ncnp.go.jp).

6　研究実施におけるモニタリングの実施に関するデータ・マネジメント室（DM 室）の関与
本研究の実施に際して、
モニタリングを　■実施する予定である　□実施する予定はない
DM 室には　■報告した　□報告予定である　□報告は不要と考えている
→NCNP 内で実施する全ての研究のうちモニタリングを実施する予定の研究については、DM 室にモニタリングの実施予定について必ず報告し、適宜の相談を受けてください。

Monitoring and Data Management:

During the execution of this research, monitoring will be conducted: ■ Monitoring is planned □ Monitoring is not planned

Reporting to the Data Management Office (DM Office): ■ Has been reported □ Reporting is planned □ Reporting is unnecessary

→ For all research conducted within NCNP where monitoring is planned, please report the planned monitoring to the DM Office and seek appropriate consultation.

///////////////**（以下、変更申請の場合のみ記載）**///////////////
///////////////Change Request Section ///////////////

7　研究計画の変更内容

7.1　変更点（変更内容及び変更理由）

Changes to the Research Plan: If there are any changes to the research plan, describe them here.

	変更項目<br>Change Item:	変更内容<br>Change Content	変更理由<br>Reason for Change
□	研究責任者の変更<br>Change of Principal Investigator		
□	研究責任者の所属変更<br>Change of Principal Investigator’s Affiliation		
■	共同研究者の削除・追加<br>（※共同研究者の追加の場合、共同研究者の COI 申告を忘れずにしてください）<br>Deletion/Addition of Co-researchers (If adding co-researchers, ensure COI declaration is submitted)	堀越勝、春口洸希、中嶋愛一郎、杉田創、矢部魁一、加藤典子、中島俊を削除	研究体制の変更のため<br>Change in research member.

		Co-researchers deleted: Masaru Horikoshi, Koki Haruguchi, Aichiro Nakajima, Hajime Sugita, Kaiichi Yabe, Noriko Kato, Toshio Nakashima.	
□	共同研究機関の削除・追加<br>Deletion or addition of collaborative research institutions		
□	依頼審査機関の追加<br>Addition of review institutions		
□	研究期間の延長<br>Extension of the research period		
□	予定症例数の変更<br>Change in the planned number of cases		
□	資金源の変更<br>Change in funding sources		
□	新たな利益相反状態の発生<br>Emergence of new conflict of interest		
□	文書の記載整備<br>Revision of document descriptions		
□	適用を受ける倫理指針の変更<br>Change in applicable ethical guidelines		
□	リクルート方法の変更<br>Change in recruitment methods		
□	組み入れ除外基準の変更<br>Change in inclusion and exclusion criteria		
□	介入方法の変更<br>Change in intervention methods		
□	検査項目の変更<br>Change in examination items		
□	解析方法の変更<br>Change in analysis methods		
□	データ管理方法の変更<br>Change in data management methods		

□	謝礼設定の変更<br>Change in compensation settings		
□	その他<br>Other		

7.2　変更した書式
□申請書（倫理システム入力項目）　□研究計画書　□説明文書　□同意書　□同意撤回書
□フローチャート　□その他（　　　　　　　　）
※書式の修正を行った箇所はアンダーラインを引いてください。

7.2 Modified Forms
□ Application Form (Ethics System Input Items)
□ Research Protocol
□ Information Document
□ Consent Form
□ Consent Withdrawal Form
□ Flowchart
□ Other (____________________)

*Please underline the sections where modifications have been made.

2. 研究の実施体制

2. Research Organization

【国立精神・神経医療研究センターにおける共同研究者の実施体制】

氏名	所属・役職	研究における役割及び責務	倫理講座の受講番号（1 年以内）	利益相反申告状況
◎伊藤正哉	認知行動療法センター・　部長	研究責任者、研究総括、研究デザイン、コーディネート、介入担当者、個人情報管理、研究モニタリング	24-0034	提出済

今村扶美	病院 臨床心理部臨床心理室・室長	介入担当者、病院コーディネート	24-0252	同上
片柳章子	認知行動療法センター・特任研究員	コーディネーター、介入担当者	24-0501	同上
蟹江絢子	認知行動療法センター・客員研究員	介入担当者	24-0389	同上
菊池安希子	認知行動療法センター・客員研究員	介入担当者	24-0494	同上
猪俣珠恵	認知行動療法センター・研究生	コーディネーター、介入担当者	24-0493	同上
高岸百合子	認知行動療法センター・客員研究員	主任研究者補助、介入担当者	24-0477	同上
中島聡美	認知行動療法センター・客員研究員	介入担当者	24-0561	同上
牧野みゆき	認知行動療法センター・研究生	コーディネーター、介入担当者	24-0395	同上
平林直次	病院 第二精神診療部・部長	病院コーディネート	24-0362	同上
宮前光宏	認知行動療法センター・リサーチフェロー	独立評価者	24-0316	同上
山口慶子	認知行動療法センター・客員研究員	独立評価者・介入担当者	24-0497	同上
横山知加	認知行動療法センター・外来研究員	独立評価者・介入担当者	24-0496	同上
田中敏志	認知行動療法センター・研究生	介入担当者	24-0523	同上
金子響介	認知行動療法センター・研究補助員	独立評価者・介入担当者	24-0071	同上
中山千秋	認知行動療法センター・客員研究員	独立評価者・介入担当者	24-0503	同上
三田村康衣	認知行動療法センター・リサーチフェロー	介入担当者、病院コーディネート	24-0492	同上
日吉史一	認知行動療法センター・研究員	介入担当者、病院連携	24-0883	同上

大澤香織	認知行動療法センター・客員研究員	介入担当者	24-0903	同上
圷京子	認知行動療法センター・研究補助員	データマネジメント	24-0681	同上
千葉俊周	認知行動療法センター・客員研究員	データ管理	24-0923	同上
重枝裕子	認知行動療法センター　研究員	独立評価者・介入担当者、コーディネート	24-0504	同上
永江亜紗	認知行動療法センター　研究補助員	独立評価者・介入担当者、コーディネート	24-0788	同上
柳百合子	認知行動療法センター・研究員	独立評価者・介入担当者、コーディネート	24-0991	同上
伊藤愛	認知行動療法センター・<br>併任研究員	介入担当者、病院連携	24-0964	同上
大庭真梨	認知行動療法センター・<br>併任研究員	解析	24-0642	同上

Collaborative Research Organiation at the National Center of Neurology and Psychiatry (NCNP):

Name	Affiliation/Position	Role and Responsibilities in Research	Ethics Course Number (within 1 year)	Conflict of Interest Declaration Status
Masaya Ito	Director, Center for Cognitive Behavioral Therapy	Principal Investigator, Research Coordination, Intervention, Personal Information Management, Research Monitoring	24-0034	Submitted

Name	Affiliation/Position	Role and Responsibilities in Research	Ethics Course Number (within 1 year)	Conflict of Interest Declaration Status
Fumi Imamura	Head, Clinical Psychology Department, NCNP Hospital	Intervention, Hospital Coordination	24-0252	Same as above
Akiko Katayanagi	Researcher, Center for Cognitive Behavioral Therapy	Coordinator, Intervention	24-0501	Same as above
Ayako Kanie	Visiting Researcher, Center for Cognitive Behavioral Therapy	Intervention	24-0389	Same as above
Akiko Kikuchi	Visiting Researcher, Center for Cognitive Behavioral Therapy	Intervention	24-0494	Same as above
Tamaki Inomata	Research Student, Center for Cognitive Behavioral Therapy	Coordinator, Intervention	24-0493	Same as above
Yuriko Takagishi	Visiting Researcher, Center for Cognitive Behavioral Therapy	Assistant Principal Investigator, Intervention	24-0477	Same as above
Satomi Nakajima	Visiting Researcher, Center for Cognitive Behavioral Therapy	Intervention	24-0561	Same as above
Miyuki Makino	Research Student, Center for Cognitive Behavioral Therapy	Coordinator, Intervention	24-0395	Same as above
Naotsugu Hirabayashi	Head, Second Psychiatry Department, NCNP Hospital	Hospital Coordination	24-0362	Same as above
Mitsuhiro Miyamae	Research Fellow, Center for Cognitive Behavioral Therapy	Independent Evaluator	24-0316	Same as above

Name	Affiliation/Position	Role and Responsibilities in Research	Ethics Course Number (within 1 year)	Conflict of Interest Declaration Status
Keiko Yamaguchi	Visiting Researcher, Center for Cognitive Behavioral Therapy	Independent Evaluator, Intervention	24-0497	Same as above
Chika Yokoyama	Visiting Researcher, Center for Cognitive Behavioral Therapy	Independent Evaluator, Intervention	24-0496	Same as above
Toshihisa Tanaka	Research Student, Center for Cognitive Behavioral Therapy	Intervention	24-0523	Same as above
Kyosuke Kaneko	Research Assistant, Center for Cognitive Behavioral Therapy	Independent Evaluator, Intervention	24-0071	Same as above
Chiaki Nakayama	Visiting Researcher, Center for Cognitive Behavioral Therapy	Independent Evaluator, Intervention	24-0503	Same as above
Yasue Mitamura	Research Fellow, Center for Cognitive Behavioral Therapy	Intervention, Hospital Coordination	24-0492	Same as above
Fumiichi Hiyoshi	Researcher, Center for Cognitive Behavioral Therapy	Intervention, Hospital Liaison	24-0883	Same as above
Kaori Osawa	Visiting Researcher, Center for Cognitive Behavioral Therapy	Intervention	24-0903	Same as above
Kyoko Akutsu	Research Assistant, Center for Cognitive Behavioral Therapy	Data Management	24-0681	Same as above
Toshimasa Chiba	Visiting Researcher, Center for Cognitive Behavioral Therapy	Data Management	24-0923	Same as above

Name	Affiliation/Position	Role and Responsibilities in Research	Ethics Course Number (within 1 year)	Conflict of Interest Declaration Status
Yuko Shigeeda	Researcher, Center for Cognitive Behavioral Therapy	Independent Evaluator, Intervention, Coordinator	24-0504	Same as above
Asa Nagae	Research Assistant, Center for Cognitive Behavioral Therapy	Independent Evaluator, Intervention, Coordinator	24-0788	Same as above
Yuriko Yanagi	Researcher, Center for Cognitive Behavioral Therapy	Independent Evaluator, Intervention, Coordinator	24-0991	Same as above
Ai Ito	Adjunct Researcher, Center for Cognitive Behavioral Therapy	Intervention, Hospital Liaison	24-0964	Same as above
Mari Oba	Adjunct Researcher, Center for Cognitive Behavioral Therapy	Analysis	24-0642	Same as above

【国立精神・神経医療研究センターにおける共同研究者以外の研究協力者の実施体制】
Organization of Research Collaborators Outside of the National Center of Neurology and Psychiatry (NCNP):
NONE

【共同研究機関における実施体制】
Organization at Collaborative Research Institutions:
NONE

【共同研究機関ではなく、既存試料・情報のやり取りのみを行う機関】
Institutions Involved Only in the Exchange of Existing Samples/Information (Not Collaborative Research Institutions):
NONE

【効果安全性評価委員会（設置する場合は、構成員を記載）】
Safety and Efficacy Evaluation Committee (If Established, List Members):

所属・職名 Affiliation, Title	氏名 Name	専門 Expertize
Japan Medical University, Professor	Toshiaki Nomura	Clinical research
Ono Institute, Director	Yutaka Ono	Psychiatry, Cognitive Behavioral Therapy

【モニタリング・監査（侵襲・介入を伴う研究の場合には必要）】
Monitoring and Auditing (Required for Research Involving Invasiveness/Intervention):

所属 Affiliation	氏名 Name	研究における役割及び責務 Role and Responsibilities in Research
認知行動療法センター National Center for Cognitive-Behavior Therapy and Research	伊藤正哉 Masaya Ito	モニタリング責任者（研究代表者） Monitoring Supervisor (Principal Investigator)

3. 研究の背景、科学的合理性の根拠及び社会的意義
3. Background of the Research, Scientific Rationale, and Social Significance

Research Question, justification of undertaking the trials (summary of relevant studies)

現在、我が国では心的外傷後ストレス障害（Posttraumatic Stress Disorder; 以下、PTSD）を患った人々へのケアが不足しており、深刻な問題となっている。生死の危険や重傷を負うようなトラウマティックな状況に接する機会は稀ではない。例えば、近年では東日本大震災、広島土砂災害、御岳山噴火に代表される自然災害、交通事故、犯罪被害（暴行被害、性被害、ドメスティック・バイオレンス（DV）、各種のハラスメント）、虐待、身体的暴力を伴う、あるいは人としての尊厳を著しく害するようないじめ、自死、職業上で体験する惨禍や高負荷業務（鉄道自殺、犯罪現場への立会、遺体処理、医療事故や突然死）等が挙げられる。

Currently, there is a significant lack of care for individuals suffering from Post-Traumatic Stress Disorder (PTSD) in Japan, which is a serious issue. Opportunities to encounter traumatic situations involving life-threatening events or severe injuries are not rare. For example, in recent years, natural disasters such as the Great East Japan Earthquake, the Hiroshima landslide, and the Mount Ontake eruption, traffic accidents, crime victimization (assault, sexual assault, domestic violence (DV), various types of harassment), abuse, physical violence, severe bullying that significantly damages one's dignity as a person, suicide, and high-stress occupational experiences (e.g., witnessing train suicides, handling crime scenes, dealing with medical accidents or sudden deaths) are cited as instances of trauma.

2013 年の刑法犯罪の被害者数（死亡・負傷）は 33,450 名（821・32629 人）、強姦・強制わいせつ・公然わいせつの被害者数はそれぞれ 1,410 人、7,672 人、1,232 人、配偶者による傷害・暴行の検挙件数は 20,444、22,717 件と報告されている(警察庁, 2014)。PTSD とは、このような危機的状況に遭遇した人に特有の精神疾患であり、再体験症状、回避、覚醒亢進、認知や気分の変化を主症状とする(American Psychiatric Association, 2013)。すなわち、苦痛な状況が再度起こっているかのような心身の反応が継続して本人を苦しめ、感情的な麻痺や、心身

が過敏で警戒している状態が慢性化し、実生活に支障を来す病態を指す。疫学調査によれば、我が国の PTSD の 1 年間の時点有病率は 0.4％であり(Kawakami, Tsuchiya, Umeda, Koenen, & Kessler, 2014)、単純計算すれば毎年約 51 万人が PTSD に苦しむと推定される。そうした患者の多くは世界的な標準治療とされる適切な心理的・医療的なケアを受けていないのが現状である。

In 2013, the number of victims (deaths and injuries) of criminal offenses under the Penal Code was reported as 33,450 (821 deaths and 32,629 injuries), while the number of victims of rape, forcible indecency, and public indecency were 1,410, 7,672, and 1,232, respectively. Furthermore, the number of arrests for injuries and assaults by spouses were 20,444 and 22,717, respectively (National Police Agency, 2014). PTSD is a mental disorder specific to individuals who encounter such critical situations, characterized by re-experiencing symptoms, avoidance, hyperarousal, and changes in cognition and mood (American Psychiatric Association, 2013). That is, it refers to a condition in which the physical and mental reactions as if the distressing situation were recurring continue to torment the individual, leading to emotional numbness and chronic hypervigilance, which significantly disrupts daily life. Epidemiological surveys indicate that the 12-month prevalence rate of PTSD in Japan is 0.4% (Kawakami, Tsuchiya, Umeda, Koenen, & Kessler, 2014), which suggests that approximately 510,000 people suffer from PTSD annually. However, many of these patients do not receive the appropriate psychological and medical care, which is considered standard treatment globally.

世界的に見ると、PTSD 治療の第一選択は、トラウマに焦点を当てた認知行動療法（Cognitive Behavior Therapy; CBT）である。これは、米国医療品質管理局(Jonas et al., 2013)、国際トラウマティック・ストレス学会(Foa, Keane, Friedman, & Cohen, 2008)、コクラン共同計画(Bisson, Roberts, Andrew, Cooper, & Lewis, 2013)など、様々な国際ガイドラインで指摘されている。CBT のなかでも、認知処理療法（CPT）の効果サイズ（待機群、minimum attention、通常治療などで定義される対照群と比較した群間効果サイズ）は g = 1.96 と非常に高い。薬物療法についての臨床試験の対照群はプラセボであり、また、精神療法にはより大きな出版バイアスが指摘されているために一概には比較できないものの、最も効果の高い薬物療法である SSRI の効果サイズは g = 0.48 と報告されている(Watts et al., 2013)。

Globally, the first-line treatment for PTSD is trauma-focused Cognitive Behavioral Therapy (CBT). This has been pointed out in various international guidelines, including those from the U.S. Agency for Healthcare Research and Quality (Jonas et al., 2013), the International Society for Traumatic Stress Studies (Foa, Keane, Friedman, & Cohen, 2008), and the Cochrane Collaboration (Bisson, Roberts, Andrew, Cooper, & Lewis, 2013). Among CBT methods, Cognitive Processing Therapy (CPT) has a very high effect size (g = 1.96) when compared with control groups (e.g., waiting list, minimum attention, Treatment-As-Usual). In contrast, although comparisons with pharmacotherapy trials are not straightforward due to the placebo-controlled nature of these trials and the larger publication bias in psychotherapy studies, the effect size of the most effective pharmacotherapy, SSRIs, is reported to be g = 0.48 (Watts et al., 2013).

トラウマに焦点を当てた認知行動療法とは、認知行動療法の考え方に基づき明確な実施手順が示された精神療法を指し、CPTや持続エクスポージャー療法がこれに当たる。我が国では持続エクスポージャー療法の臨床試験が実施され、その有効性の一端が示唆されつつある(Asukai, Saito, Tsuruta, Kishimoto, & Nishikawa, 2010)。一方、認知処理療法は全世界的にみてもここ15年ほどで急速に研究成果が集積されている新しい治療法である。エビデンスのあるPTSD治療として、米国退役軍人局において最も普及しているのがCPTである。米国では研究が発展し、現在では個々の患者に最適なCPTの実施法を同定するために、約26億円を投じて400名規模の臨床試験が進行している。

Trauma-focused Cognitive Behavioral Therapy refers to a psychotherapy with clearly defined implementation procedures based on CBT principles, with CPT and Prolonged Exposure Therapy being examples. In Japan, clinical trials of Prolonged Exposure Therapy have been conducted, and some efficacy has been suggested (Asukai, Saito, Tsuruta, Kishimoto, & Nishikawa, 2010). On the other hand, Cognitive Processing Therapy is a newer treatment method that has rapidly accumulated research results worldwide over the past 15 years. As evidence-based PTSD treatment, CPT is the most widely used therapy at the U.S. Department of Veterans Affairs. In the U.S., research is advanced, and currently, a large-scale clinical trial involving about 400 participants and costing approximately 2.6 billion yen is underway to identify the optimal method of CPT implementation for individual patients.

こうした背景を踏まえ、我々の研究チームは9年を掛けてCPTの日本への導入を進めてきた。過去3年の研究では臨床研究の実施体制を整え、個人CPTを17例、集団CPTを7例実施した。CPTおよび臨床試験の実施可能性が確認され、より厳格なランダム化比較試験に着手する段階に至った。

In light of this background, our research team has been working for nine years to introduce CPT to Japan. Over the past three years, we have established the infrastructure for conducting clinical research and have implemented individual CPT for 17 cases and group CPT for seven cases. The feasibility of conducting CPT and clinical trials has been confirmed, and we are now ready to embark on a more rigorous randomized controlled trial.

Choice of comparators

現在の日本の医療現場において、心的外傷後ストレス障害に対して広く実施されている治療は、精神科医による薬物療法、生活指導を中心とした臨床管理、支持的カウンセリングであると考えられる。治療資源が充実している施設においては、臨床心理士などによる支持的カウンセリングを実施しているところもあると考えられる。欧米のエビデンスや治療ガイドラインを参考にすれば、持続エクスポージャー療法、認知処理療法、眼球運動による脱感作および再処理法などの治療選択肢が推奨されるものの、現時点で我が国の医療現場において検

証されたエビデンスは乏しく、また、一般の医療現場において使用されている治療ではないと考えられる。そこで、本試験の対照群は、我が国における一般的な通常治療を継続する“通常治療群”とする。

Currently, in Japan’s healthcare settings, the common treatments for PTSD are pharmacotherapy by psychiatrists, clinical management centered on lifestyle guidance, and supportive counseling. In facilities with abundant treatment resources, it is likely that supportive counseling by clinical psychologists is also implemented. Although treatment options such as Prolonged Exposure Therapy, Cognitive Processing Therapy, and Eye Movement Desensitization and Reprocessing (EMDR) are recommended based on Western evidence and treatment guidelines, the evidence verified in Japan’s medical settings is still limited, and these treatments are not widely used in general medical settings. Therefore, in this trial, the control group will continue to receive the standard treatment commonly provided in Japan, referred to as the "Treatment-As-Usual Group."

4. 研究の目的及び意義

4. Research Objectives and Significance

Objectives

Research hypothesis

心的外傷後ストレス障害患者の、PTSD臨床診断面接尺度（Clinician Administered PTSD Scale for DSM-5; CAPS-5）で測定される心的外傷後ストレス症状の軽減において、通常治療に認知処理療法を加えることは、通常治療単独よりも有効である。

Objectives:

Research Hypothesis:

Adding Cognitive Processing Therapy (CPT) to Treatment-As-Usual for patients with Post-Traumatic Stress Disorder (PTSD) will be more effective than Treatment-As-Usual alone in reducing PTSD symptoms as measured by the Clinician-Administered PTSD Scale for DSM-5 (CAPS-5).

Study objectives

Primary objective

本臨床試験は心的外傷後ストレス障害を対象として、通常治療に認知処理療法を併用する介入群と、通常治療のみの対照群について、臨床的有効性としての主要評価項目を17週のCAPS-5に設定し、介入群の対照群に対する優越性を検証する。また、安全性は有害事象を指標として比較検討する。

Study Objectives:

Primary Objective:

This clinical trial targets patients with PTSD and aims to verify the superiority of an intervention group receiving CPT in addition to Treatment-As-Usual, compared to a control group receiving Treatment-As-Usual only. The primary efficacy endpoint is the CAPS-5 score at 17 weeks, with safety evaluated by comparing adverse events between groups.

PICO にて本研究の仮説を定式化すると、以下のようになる。
Participants: 心的外傷後ストレス障害患者が
Intervention: 通常治療に加えて認知処理療法を実施すると、
Comparison: 通常治療のみに比して、
Outcome: CAPS-5 においてより顕著な症状の改善を示す。
Time: 症状の評価時期は、登録より 17 週後である。

Here's the formulation of the hypothesis for the study using the PICO framework:

PICO Hypothesis Formulation:

- **Participants:** Patients with post-traumatic stress disorder (PTSD)
- **Intervention:** Implementation of Cognitive Processing Therapy (CPT) in addition to usual treatment
- **Comparison:** Compared to usual treatment alone
- **Outcome:** Demonstrates a more significant improvement in symptoms as measured by the CAPS-5
- **Time:** The assessment of symptoms will be at 17 weeks after enrollment

Secondary objectives
・17 週時点での患者による自己報告の心的外傷後ストレス症状（PTSD Checklist for DSM-5）について、介入群の対照群に対する優越性を検証する。
・17 週時点での治療反応割合について、介入群の対照群に対する優越性を検証する。
・介入群および対照群における有害事象の発生状況を比較する。

Secondary Objectives:

- To verify the superiority of the intervention group over the control group in terms of self-reported PTSD symptoms at 17 weeks using the PTSD Checklist for DSM-5 (PCL-5).
- To verify the superiority of the intervention group over the control group in terms of the treatment response rate at 17 weeks.
- To compare the occurrence of adverse events between the intervention and control groups.

5. 研究の方法及び期間

5. Research Methods and Period

Trial design

SPINET は、評価者盲検、並行群間、単施設、ランダム化、優越性検証比較試験としてデザインされている。ランダム化は、トラウマ体験（単回性 vs. 持続性）を層別因子とした最小化法を用い、割付比は 1:1 である。

Trial Design:

SPINET is designed as a blinded-evaluator, parallel-group, single-center, randomized superiority trial. Randomization will be stratified by the type of trauma experience (single vs. multiple/ongoing) using a minimization method with a 1:1 allocation ratio.

【設定根拠】

認知処理療法は治療者と被験者に対して盲検化するのが不可能である。ただし、独立評価者に対しては盲検化が可能であることから、評価者バイアスを排除するために評価者盲検とした。実施施設は、国立精神・神経医療研究センターの単施設である。通常治療群と、通常治療に加え CPT を実施する群を比較する並行群間試験であり、前者に対する後者の優越性を検証する。割り付け比は１：１である。トラウマ体験が一回のみの場合（単回性、例：事故、事件、災害などの一度きりの被害）と、長い経過を有する場合（持続性、例：虐待やドメスティックバイオレンスなど、長期間及び/または複数にわたる被害）では治療反応が異なる可能性があるために、トラウマ体験の特徴（単回性 vs. 持続性）を層別因子とした。

Justification of the Design:

It is impossible to blind both the therapist and the subject to the treatment in Cognitive Processing Therapy. However, it is possible to blind the independent evaluators, thus eliminating evaluator bias. The study will be conducted at a single facility, the National Center of Neurology and Psychiatry (NCNP), comparing a group receiving Treatment-As-Usual plus CPT with a group receiving Treatment-As-Usual only. The allocation ratio is 1:1. The type of trauma experience (single vs. multiple/ongoing) is considered a stratification factor because treatment responses may differ depending on the trauma experience.

Study setting

本研究は、国立精神・神経医療研究センター（National Center of Neurology and Psychiatry; NCNP）病院（日本国、東京郊外）にて実施される。当病院は、併設されている研究機関とともに、精神疾患、神経疾患、筋疾患及び発達障害の克服を目指した研究開発を行い、その成果をもとに、高度先駆的医療を提供している。

Study Setting:

This study will be conducted at the NCNP hospital, located in the suburbs of Tokyo, Japan. The hospital, together with the attached research institute, engages in research and development aimed at overcoming mental, neurological, muscular, and developmental disorders, and provides advanced medical care based on its research findings.

Recruitment

Recruitment:

(1) リクルート戦略

国立精神・神経医療研究センター病院の医師や外部の関連医療機関に本研究を告知し、認知処理療法によるPTSD治療を希望する患者の紹介を主治医より受ける。外部の医療機関に主治医をもつ患者の場合には、本研究の選択基準を伝えるとともに、PC-PTSDで陽性となるか、PCL-5で38点以上の場合に本研究に該当する可能性が高い旨[1]を伝え、NCNP病院への初診受診を促す。NCNP病院の医師（とくに初診医）に対しては、PTSDへの認知行動療法のために当院初診を希望する患者が紹介される可能性を周知するとともに、そのような患者がいた場合には、精神リハビリテーション部の認知行動療法インテークに予約を入れていただくよう伝える。このようにして、NCNP病院内の医師より紹介を受けるとともに、病院の臨床心理室の認知行動療法プログラムと連携し、適切と思われる患者がいる場合には紹介が得られるようにする。リクルートが思わしくない場合、国立精神・神経医療研究センター病院の近縁にあるクリニック等に対して本研究を告知し、適切と思われる患者の紹介を受ける。

(1)Recruitment Strategy:

The study will be advertised to physicians at the NCNP hospital and external affiliated medical institutions to receive referrals from primary physicians for patients who wish to undergo PTSD treatment using CPT. If the patient has a primary physician at an external medical institution, the study's selection criteria will be communicated along with the information that patients who test positive on the PC-PTSD or score 38 or higher on the PCL-5 are likely to be eligible for this study. The patient will be encouraged to visit the NCNP hospital for an initial consultation. The physicians at NCNP (especially those responsible for initial consultations) will be informed that patients seeking CBT for PTSD may be referred to the hospital. If such patients are found, they will be instructed to schedule an intake for CBT at the Department of Psychiatric Rehabilitation. In this way, patients will be referred by NCNP hospital doctors, and when appropriate, patients will also be referred through collaboration with the hospital's Clinical Psychology Department CBT program. If recruitment is not satisfactory, the study will be announced to clinics near the NCNP hospital, and appropriate patients will be referred.

(2) 試験実施予定期間

被験者の登録予定期間

2016年4月～2023年8月

試験実施予定期間：

2016年4月～2024年4月

ただし、試験が中止・終了されれば、被験者の登録を終了する。

(2) Planned Period for Patient Enrollment:

Planned patient enrollment period:

April 2016 to August 2023.

Planned study period:

April 2016 to April 2024.

If the study is suspended or terminated, patient enrollment will be discontinued.

(3) 患者リクルート見積もり　これまで、2016 年 4 月から 2021 年 2 月までの 58 ヶ月において 41 例の登録を行った。毎月換算だと 0.7 例の登録である。ここから、目標症例数 58 例（残り 17 例）を登録するには、あと 24 ヶ月のリクルート期間（2023 年 2 月まで）を要すると見積もられる。若干の遅れを想定して、登録予定期間は 2023 年 8 月とした。最後の登録例の追跡評価は、2024 年 4 月となる予定である。

(3) **Estimated Recruitment Rate:**

From April 2016 to February 2021, over 58 months, 41 patients were enrolled, resulting in a monthly enrollment rate of 0.7 patients. Based on this, it is estimated that an additional 24 months will be required to enroll the remaining 17 patients to reach the target of 58 patients, setting the recruitment deadline to February 2023. Accounting for potential delays, the enrollment period is extended to August 2023, with the final follow-up assessment planned for April 2024.

（1）研究実施期間

研究実施許可受領後から 2026 年 3 月 31 日まで

（研究対象者登録締切日：2026 年 3 月 31 日、ただし、試験が中止・終了されれば、被験者の登録を終了する。）

(1) Research Implementation Period

From the receipt of research implementation approval until March 31, 2026

(Deadline for subject registration: March 31, 2026. However, subject registration will be terminated if the trial is discontinued or completed.)

（2）研究の種類・デザイン

【介入研究】

評価者盲検、並行群間、単施設、ランダム化、優越性検証比較試験

(2) Research Type and Design:

Intervention Study:

Evaluator-blinded, parallel-group, single-center, randomized superiority trial.

（3）予定する研究対象者数

Sample size

介入群（通常治療と認知処理療法の併用群）29 例

対照群（通常治療）29 例

計 58 例

(3) Planned Number of Research Participants:

Intervention Group (Treatment-As-Usual + Cognitive Processing Therapy): 29 cases

Control Group (Treatment-As-Usual): 29 cases

Total: 58 cases

附属研究（脳画像研究）：通常治療＋認知処理療法実施群 100 例（上記ランダム化比較試験と重複する症例もある）

上記を総計すると 100 例

Ancillary Study (Brain Imaging Research): Treatment-As-Usual + Cognitive Processing Therapy: 100 cases (some of which overlap with the randomized controlled trial)

Including the above, the total number of cases is 100.

【設定根拠】

治療終結時の PTSD 症状（CAPS）を主要評価項目とした、通常治療や待機群と対照したランダム化比較試験による CPT の効果サイズは、Hedge's g = 1.40 [95%CI 0.85–1.95] (*k* = 4, *N* = 299)[2]と報告されている。本研究では線形混合モデルにより主要評価項目を検討する。線形混合モデルの例数設計のための公式である Diggle (2002)に基づくと、本研究の主要評価項目である 17 週時点での CAPS-5 得点における介入群と対照群の平均値の差をα=0.05、検定力=0.9、標準効果サイズ=1.40 [95%CI 0.85–1.95]、測定時点を 3 時点 (pre, middle post)、共分散構造を 1 次の自己回帰構造 (AR(1))、自己相関の範囲を 0.2~0.8 と想定した場合に、必要症例数の範囲は片群 3-24 名となる。最終的に、CPT-C のドロップアウト率 22% [3]分の人数(5 名)を、既出の必要症例数範囲の上限 24 名に加え、29 名を片群の必要例数と設定した。

本研究では、臨床試験として 58 例の登録が完了するまでは臨床試験としてのリクルートを継続し、そのようにしてリクルートした参加者に対してはランダム割付の手続きへ進む。58 例の登録後に研究紹介された場合には、附属研究を含め目標症例 100 例に達するまで、すべての参加者を「通常治療＋認知行動療法群」に割付する。ただし、この参加者は附属研究のための割付であり、本臨床試験の Primary Outcome Paper のデータとしては使用しない。

Rationale for Setting the Sample Size:

The effect size of Cognitive Processing Therapy (CPT) for PTSD symptoms (CAPS) compared to Treatment-As-Usual or a waiting list control group in randomized controlled trials is reported as Hedge's g = 1.40 [95% CI 0.85–1.95] (k = 4, N = 299). In this study, the primary endpoint will be analyzed using a linear mixed model. Based on the formula for sample size calculation using linear mixed models by Diggle (2002), assuming the difference in mean CAPS-5 scores between the intervention and control groups at 17 weeks, with α=0.05, power=0.9, standard effect size=1.40 [95% CI 0.85–1.95], three measurement points (pre, middle, post), and a first-order autoregressive

covariance structure (AR(1)), with a correlation range of 0.2 to 0.8, the required sample size ranges from 3 to 24 cases per group. Considering a dropout rate of 22% in CPT-C, five additional cases were added to the upper limit of the required sample size range, resulting in a required sample size of 29 cases per group.

In this study, recruitment for the clinical trial will continue until the target of 58 cases is reached. After 58 cases are registered, if further participants are referred, they will be allocated to the "Treatment-As-Usual + Cognitive Behavioral Therapy Group" as part of the additional study, until the target of 100 cases is reached. However, data from these participants will not be used in the primary outcome paper for this clinical trial.

（4）研究のアウトライン

(4) Outline of the Research:

Participant timeline

(1)試験のタイムライン

本研究への参加の前提として、患者が NCNP 病院か外部の医療機関に通院し主治医を持っていることが必要である。外部機関に主治医がいる場合には、その主治医の判断により、PTSD への認知処理療法を希望する旨とともに NCNP 病院へと患者を紹介してもらう。NCNP 病院で初診を受け、担当医が決まった場合には、その担当医が認知行動療法の実施可否を確認する。NCNP 担当医が認知行動療法を推奨する場合には、その担当医が NCNP 病院精神リハビリテーション部の認知行動療法インテーク枠に予約を入れる。NCNP 病院認知行動療法インテーク医が認知行動療法のためのインテークを行った後、臨床心理室でのカンファレンスにより、研究紹介への可否が判断される。なお、これらのプロセスにおいて、PCL-5 において 38 点以上を研究紹介の目安とする。

Participant Timeline:

(1) Trial Timeline:

Participation in this study requires that patients are receiving outpatient care at the NCNP hospital or an external medical institution and have a primary physician. If the patient has a primary physician at an external institution, the primary physician will decide whether to refer the patient to the NCNP hospital for PTSD treatment using CPT. After the patient receives an initial consultation at the NCNP hospital and a primary physician is assigned, the physician will determine whether CBT is appropriate. If CBT is recommended, the primary physician will schedule an intake at the Department of Psychiatric Rehabilitation at the NCNP hospital. The CBT intake physician at NCNP will conduct the intake, and eligibility for study referral will be determined during a conference at the Clinical Psychology Department. The PCL-5 score of 38 or higher will serve as a criterion for study referral.

紹介された患者には、SPINET 研究の説明を行い、同意を取得する。説明と同意の手続きを経て、ベースライン評価により選択基準を満たした参加者は、介入群あるいは対照群にランダムに割付けられる。介入期間は 16 週（Week 0–16）である。評価は、中間評価（Week 8）と介入後評価（Week 17）、追跡評価（Week 34）を行う。

Patients referred to SPINET will be provided with an explanation of the study, and informed consent will be obtained. After obtaining consent, baseline assessments will be conducted to confirm eligibility, and participants will be randomly assigned to the intervention or control group. The

intervention period will last 16 weeks (Week 0-16). Assessments will be conducted at mid-treatment (Week 8), post-treatment (Week 17), and follow-up (Week 34).

以下、フローを示す。

1. 外部医療機関に主治医がいる場合には、その主治医が NCNP 病院へ患者紹介
2. NCNP 病院初診
3. NCNP 担当医（初診医のこともあれば、そうでない場合もある）が CBT へ紹介。

※NCNP 病院に主治医がいる場合は、その主治医が CBT へ紹介。以降の流れは同じ。

4. CBT インテーク医がインテーク実施
5. NCNP 病院臨床心理室カンファレンスで研究紹介の検討
6. SPINET への紹介
7. 説明と同意
8. 症状評価
9. 評価者会議にて登録可否を最終決定
10. 割付・CPT インテーク
11. 介入開始
12. 中間評価（8 週）
13. 介入後評価（17 週）
14. 追跡評価（34 週）

Below is the flow of the study:

1. If the patient has a primary physician at an external institution, the primary physician refers the patient to the NCNP hospital.
2. Initial consultation at the NCNP hospital.
3. The NCNP primary physician (either the initial consultant or another) refers the patient to CBT.
4. If the patient has a primary physician at NCNP, that physician refers the patient to CBT.
5. The CBT intake physician conducts the intake.
6. Eligibility for study referral is determined during the Clinical Psychology Department conference.
7. Referral to SPINET.
8. Explanation and consent.
9. Symptom evaluation.
10. Final registration decision by the evaluation committee.
11. Randomization and CPT intake.
12. Intervention begins.
13. Mid-treatment evaluation (Week 8).
14. Post-treatment evaluation (Week 17).
15. Follow-up evaluation (Week 34).

(2) 被験者の試験参加予定期間

組入れ期間; -6–0 週間、介入期間; 1–16 週、最終評価; 17 週、追跡期間 18–34 週、計 34 週間

CONSORT ダイアグラムによる患者の流れを Figure1 に、SPIRIT にて推奨されているテンプレートを用いたタイムラインを Figure 2 に示す

(2) Planned Participation Period for Participants:

- Enrollment period: -6 to 0 weeks.
- Intervention period: 1 to 16 weeks.
- Final evaluation: Week 17.
- Follow-up period: 18 to 34 weeks.
- Total: 34 weeks.

A CONSORT diagram showing the flow of patients and a timeline template recommended by SPIRIT will be presented in Figures 1 and 2, respectively.

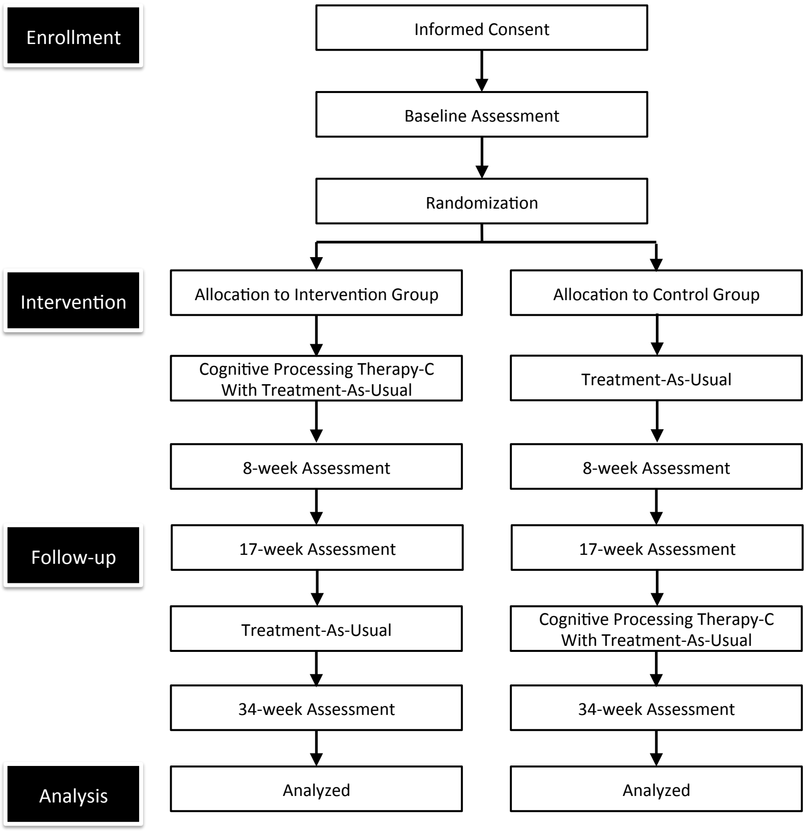

Figure 1. CONSORT Diagram

					ENROLLMENT			INTERVENTION																POST	FU
TIME POINT(Week)					-2	-1	0	1	2	3	4	5	6	7	8	9	10	11	12	13	14	15	16	17	34
Visit					V2	V3	V4	V5	V6	V7	V8	V9	V10	V11	V12	V13	V14	V15	V16	V17	V18	V19	V20	V21	V22
（Burden for patient(minutes　））																									
**ENROLLMENT**																									
		Informed Concent		(50)	PI/C																				
		Randomization		(5)			PI/C																		
		Intake		(30)			Th[a)]																		
**INTERVENTION**								Cognitive Processing Therapy–Cognitive with Treatment-As-Usual<br>Treatment-As-Usual																	TAU<br>TAU+CPT
**ASSESSMENTS**																									
IE	Primary Outcome	PTSD Severity	CAPS-5	(60)		IE									IE									IE	IE
	Other Outcome	Diagnosis	MINI	(20)		IE																		IE	IE
	Blinding		IEKNO	-											IE									IE	IE
Pt	Other Outcomes	PTSD Severity	PCL-5 plus	(5)		Pt		PtC	PtC	PtC	PtC	PtC	PtC	PtC	Pt	PtC	PtC	PtC	PtC	PtC	PtC	PtC	PtC	Pt	Pt
		Depression	PHQ-9	(2)		Pt		PtC	PtC	PtC	PtC	PtC	PtC	PtC	Pt	PtC	PtC	PtC	PtC	PtC	PtC	PtC	PtC	Pt	Pt
		Suicidal Ideation	SIDAS	(1)		Pt		PtC	PtC	PtC	PtC	PtC	PtC	PtC	Pt	PtC	PtC	PtC	PtC	PtC	PtC	PtC	PtC	Pt	Pt
		Quality of Life	EQ-5D–5L	(2)		Pt									Pt									Pt	Pt
		Functioninng	SDS	(2)		Pt									Pt									Pt	Pt
	Treatment Mechanism	Trauma related cognition	PMBS	(4)			Pt								Pt									Pt	Pt
			TRGI	(4)			Pt								Pt									Pt	Pt
		Emotion Regulation Questionnaire	ERQ	(3)			Pt								Pt									Pt	Pt
	Process Measure	Working Alliance	SRS	(1)				Pt<br>Th					Pt<br>Th					Pt<br>Th							
Th	Safety	Adverse Event	AE	(3)				Th	Th	Th	Th	Th	Th	Th	Th	Th	Th	Th	Th	Th	Th	Th	Th	IE	IE
	Adherence	Homework Compliance	HC	(3)					Th	Th	Th	Th	Th	Th	Th	Th	Th	Th	Th	Th	Th	Th	Th		
		Treatment Adherence	TAS	-				Third Person (20% of the sessions)																	
Biological evaluation			MRI	(40)			X																	X	X
Burden for Participants(minutes)					(50)	(92)	(86)	(9)	(8)	(8)	(8)	(8)	(9)	(8)	(83)	(8)	(8)	(9)	(8)	(8)	(8)	(8)	(8)	(143)	(143)

Figure 2. Overall description of the measures and its timepoints.
PI: Primary Investigator, C: Coordinator, IE: Independent Evaluator, Pt: Participant, PtC: Participant during CPT treatment, Th: Therapist, SV: Supervisor
PCL-5 plus has three additional items for dissociation.
MRI will be conducted at before and after the CPT intervention.

（5）研究に用いる医薬品・医療機器、治療法等の情報
(5) Information on Pharmaceuticals, Medical Devices, and Treatments Used in the Research

Interventions

Interventions for each group

被験治療：通常治療に認知処理療法を併用した治療

対照治療：通常治療

Interventions for Each Group:

Intervention Group: Treatment-As-Usual combined with Cognitive Processing Therapy (CPT)

Control Group: Treatment-As-Usual only

(1) 認知処理療法（Cognitive Processing Therapy-Cognitive; CPT）

(1) Cognitive Processing Therapy-Cognitive(CPT)

治療構造：CPT の実施マニュアル[4]に基づき、CPT のマテリアルマニュアル[5]を使用して実施される。一人のセラピストとの対面もしくはオンラインの個人療法であり、週に一回、約 50 分の面接を実施する。被験者に対して、治療において学んだスキルを日常生活において復習したり練習したりする宿題が出される。

Treatment Structure: CPT will be implemented according to the CPT manual, using materials specified in the CPT materials manual. It will be conducted as individual therapy, either face-to-face or online, with a single therapist, involving approximately 50-minute sessions once a week. Participants will be given homework to review and practice the skills learned in therapy during their daily lives.

治療スケジュール：被験者に CPT マニュアルに基づく CPT を、16 週間の実施期間に 12–16 回実施する。CPT は全 12 回で構成されるが、介入期間中に 16 回まで回数を増やすことができる。なお、共同研究者である CPT 実施者とスーパーバイザーとの間で該当被験者に追加セッションを行うことにより CPT の終結が円滑にできると判断された場合、研究責任者が追加セッションの可否を判断する。ただし、その場合であっても 17 週時点での評価を主要評価項目とする。また、CPT 実施の補助のために陪席者を治療に置くことがある。陪席者は共同研究者か研究協力者であり、本研究のスーパーバイザーのもと訓練を受けている者に限る。

Treatment Schedule: Participants will receive 12 to 16 sessions of CPT over 16 weeks, based on the CPT manual. Although CPT consists of 12 sessions, the number of sessions can be increased to 16 during the intervention period if necessary. If the CPT therapist and the supervisor deem additional sessions helpful for completing CPT smoothly, the principal investigator will decide whether additional sessions are allowed. However, the primary evaluation will be based on the 17-week point regardless. A facilitator, who is a co-researcher or research collaborator trained under the study's supervisor, may accompany the therapy for support.

治療内容：治療は、トラウマティックな出来事とその後の経過において、患者が抱くようになった特定の認知（スタックポイント）が、PTSD 症状を作り維持させているという概念化のもとに、スタックポイントの同定と修正を目指す。初回ではこのような治療原理を患者に心理教育し、治療への動機付けを高める。セッション 2 では、出来事がなぜ起こったか、出来事によってどのように自分の人生が変化したかを筆記してきてもらい、これをセッション内外で読み上げることで、自然な感情を感じるとともに、スタックポイントを同定する。スタックポイントは複数であることが多いため、スタックポイント・ログに随時追加し、のちの再構成で用いる。セッション 3 から ABC 用紙を使って、患者が自らの体験を俯瞰してモニタリングし、状況・思考・感情を区別できるように練習する。セッション 5 では考え直し用紙、セッション 6 では問題ある思考パターン用紙を導入し、徐々に自らのスタックポイントへの考え直しに取り組む。以上までの CPT 治療の前半は、出来事がなぜ起こったか、自分のせいで起こったのだという自責感や罪悪感に焦点を当てて取り組む。セッション 7 では、これまでの用紙を統合した信念を考え直す用紙を導入し、こ

れを用いる練習を行う。セッション8から12においては、5つのテーマ（安全、信頼、力とコントロール、価値、親密さ）のそれぞれに焦点を当てながら、スタックポイントの考え直しに取り組む。セッション10では、コンプリメントを与え・受けるという宿題と、自分にとって楽しい活動に取り組むという宿題も出される。最終セッションでは、出来事の意味を宿題として筆記し直し、セッション内で読み上げることで、治療を振り返るとともに残された課題について確認する。

Treatment Content: The therapy is based on the concept that specific cognitions (stuck points) that patients develop in response to traumatic events and their aftermath create and maintain PTSD symptoms. The goal is to identify and modify these stuck points. The initial session involves psychoeducation on these therapeutic principles to increase motivation. In session 2, the patient writes about why the event occurred and how it has changed their life, reads it aloud in and out of the session, and identifies stuck points. Stuck points are often multiple, so they are added to a log to be used in later reconstruction. From session 3, the patient uses an ABC worksheet to monitor and distinguish situations, thoughts, and emotions. In session 5, the patient is introduced to a "reconsideration" worksheet, and in session 6, a worksheet for problematic thinking patterns is introduced, gradually working on reconsidering their stuck points. The first half of CPT focuses on guilt or self-blame for why the event occurred. In session 7, the patient integrates the worksheets into a reconsideration of beliefs and practices using them. In sessions 8 to 12, the patient works on reconsidering stuck points with a focus on five themes: safety, trust, power/control, esteem, and intimacy. In session 10, the patient is assigned homework to give and receive compliments and to engage in enjoyable activities. The final session involves rewriting the meaning of the event as homework, which is read aloud in the session to reflect on the treatment and confirm any remaining issues.

CPT担当者：臨床心理士あるいは医師の資格を持ち、CPTマニュアルに準拠した臨床訓練を十分に受けた共同研究者がCPTを実施する。治療者となるためには、14時間以上のCPT研修参加と2例の治療陪席経験もしくは治療録音記録の視聴、最低1例の訓練事例の経験を前提とする。

CPT Therapist: CPT will be implemented by co-researchers who are licensed clinical psychologists or physicians and have received sufficient clinical training according to the CPT manual. To qualify as a therapist, they must have participated in at least 14 hours of CPT training, have experience in accompanying at least two cases during treatment or listening to recorded sessions, and have treated at least one training case.

（2）通常治療：

本試験の対照群は、我が国における一般的な通常治療を継続する“通常治療群”とする。本試験は、国立精神・神経医療研究センター病院を含む何らかの医療機関に主治医を持つ患者を対象としており、対照群に割り付けられた場合には、その主治医（精神科医）による臨床管理、非系統的な支持療法、薬物療法が通常治療となると考えられる。日本ト

ラウマティックストレス学会は、トラウマを体験した患者への対応や薬物療法についてのガイドラインを出版している[6]。これはプライマリケア医に向けたガイドラインであるものの、精神科医療の場面でも参照しうる日本における唯一のガイドラインである。当ガイドラインによれば、トラウマ体験をした患者への対応として、トラウマ体験を共感的に、丁寧に聞くこと（強引に聞き出さない）や患者を非難したり被害を軽視したりしないことが記されている。その上で、PTSD についての疾病教育のほか、呼吸法、筋弛緩法、自律訓練など、患者自身で症状に対してコントロール感を持てるような対処法を伝えることが推奨されている。また、現実的な問題に対して優先順位を整理し、必要な相談機関を紹介することが推奨されている。薬物療法としては、セロトニン再取り込み阻害薬のうち、米国の食品医薬品局で認可されているパロキセチンとセルトラリンを一剤選択肢、少量から投与し、有害事象発現を見ながら、投与可能な最大量まで増量し、寛解すれば少なくとも 1 年間投薬を継続する維持療法を行うことが再発防止の上で推奨されている。なお、同ガイドラインにおいては、抗精神病薬のオランザピン、クエチアピンの有効性が紹介されており、ベンゾジアゼピン系抗不安薬の長期連用を推奨されていない。我が国では、パロキセチン（パキシル）について PTSD の効能・効果があると厚生労働省が承認している。

(2) **Treatment-As-Usual:**

The control group in this study will continue to receive Treatment-As-Usual commonly provided in Japan's medical institutions, referred to as the "Treatment-As-Usual Group." Participants assigned to the control group are expected to receive clinical management, non-structured supportive therapy, and pharmacotherapy from their primary psychiatrist. The Japanese Society for Traumatic Stress Studies has published guidelines for responding to trauma patients and pharmacotherapy, which are available to primary care physicians and can also be referenced in psychiatric settings. These guidelines recommend that healthcare providers listen empathetically and carefully to patients' traumatic experiences without forcing them to disclose details and not to minimize or criticize the trauma. Additionally, the guidelines suggest providing disease education on PTSD, teaching patients coping techniques such as breathing exercises, muscle relaxation, and autogenic training to help them gain a sense of control over their symptoms, and referring them to appropriate consultation services for practical problems. Pharmacotherapy recommendations include selecting one of the SSRIs (paroxetine or sertraline) approved by the U.S. Food and Drug Administration, starting with a low dose, increasing it to the maximum tolerable dose while monitoring for adverse events, and maintaining the medication for at least one year if remission is achieved. Moreover, the guidelines introduce the effectiveness of antipsychotics such as olanzapine and quetiapine and discourage the long-term use of benzodiazepine anxiolytics.

倫理的な観点から、本研究のために通常治療を不合理なまでに制限することはないが、主治医にはなるべく薬物療法を安定化してもらうことと、他の系統的な精神療法や電気けいれん療法を実施しないことを予め伝える。通常治療の内容（頻度、時間、薬物療法の種

類、臨床管理・指導の内容）については、ベーライン時に聴取し、その後、Visit ごとに頻度・時間、および内容の変更について患者に確認し、CRF に記録する。

From an ethical standpoint, Treatment-As-Usual will not be unreasonably restricted for this study. However, primary physicians will be asked to stabilize pharmacotherapy as much as possible and to refrain from conducting other structured psychotherapies or electroconvulsive therapy. The content of Treatment-As-Usual (frequency, duration, type of pharmacotherapy, clinical management) will be recorded at baseline and confirmed with the patient at each visit, with any changes documented in the Case Report Form (CRF).

Specific considerations for conducting online session　新型コロナウイルス感染症等の感染予防のために、「オンライン診療の適切な実施に関する指針（厚生労働省平成 30 年３月、令和元年 7 月一部改定）」「医療情報システムの安全管理に関するガイドライン第 5 版　(厚生労働省 平成 29 年 5 月）」を参考として、以下の条件のもとにオンラインでの実施を可とする。

Specific Considerations for Conducting Online Sessions:

To prevent the spread of COVID-19 and other infectious diseases, online sessions can be conducted under the following conditions, in accordance with the "Guidelines for the Proper Implementation of Online Medical Consultations" (Ministry of Health, Labor and Welfare, March 2018, partially revised in July 2019) and the "Guidelines for the Safe Management of Medical Information Systems, 5th Edition" (Ministry of Health, Labor and Welfare, May 2017).

(1) オンラインセッションの定義・手段・内容

①定義：ここでは「情報通信機器を活用したリアルタイムの面接」を指す。
②手段：web 会議システム等を用いた映像および音声による面接を想定する。
③内容：認知処理療法の治療面接および中間（8 週）以降に予定されている症状評価面接をオンラインセッションとして実施することを想定する。

(1) Definition, Means, and Content of Online Sessions:

1. Definition: "Real-time interviews utilizing information and communication devices."
2. Means: Video and audio interviews using web conferencing systems.
3. Content: Cognitive Processing Therapy sessions and symptom evaluation interviews scheduled after mid-treatment (Week 8) will be conducted as online sessions.

(2) オンラインセッション実施の要件

オンラインセッションを安心・安全に実施するために、以下の要件をすべて満たす必要がある。

①研究参加者自身が、感染予防の観点からオンライン実施を求めている
②感染対策の観点から、対面での実施が望ましくない社会状況下にある

③主治医の許可が得られている
④対面での緊急対応が必要となる急病急変の履歴が過去１年間ない（例：予定外の診察）
⑤オンライン実施に要する情報通信機器の準備や通信に係る費用を研究参加者自身が負担することに同意している
⑥使用する情報通信機器に関して、参加者が自らの責任で必要なアプリケーションのインストールやセキュリティ対策を実施することに同意している
⑦危機的な状況になったときのための安全計画（例：少なくとも一人の緊急連絡先、主治医および最寄りの救急医療機関の連絡先の共有）を事前に作成することに同意している
⑧急病急変により即座の対応が必要になったときに、必要性が認められれば、研究スタッフから緊急連絡先や医療機関への情報提供を行うことに予め同意している
⑨研究参加者が自宅に、気が散ることのない静かでプライベートな空間を用意することができる
⑩研究スタッフが主治医との診察を勧めた場合に、それに応じることに研究参加者が予め同意している
⑪研究スタッフが、その専門家としての観点から、オンラインセッションを継続することが適切ではないと判断し、オンラインセッションが中断となる場合があることに研究参加者が予め同意している
⑫研究で用いるアカウント等の情報を、緊急連絡先等の研究に関わりのない第三者に提供しないことに研究参加者が同意している
⑬オンライン実施に関する説明と同意の手続きを別途実施し、説明文書に記す事項に同意が得られている

(2) Requirements for Conducting Online Sessions: Online sessions can only be conducted safely and securely if all of the following conditions are met:

1. The research participant requests online sessions due to infection prevention concerns.
2. Face-to-face sessions are not desirable due to societal conditions related to infection control.
3. The primary physician's permission is obtained.
4. The participant has not experienced any acute illnesses or unexpected medical conditions in the past year that required emergency face-to-face consultations.
5. The participant agrees to bear the costs associated with the preparation of information and communication devices and the communication required for online sessions.
6. The participant agrees to take responsibility for installing the necessary applications and implementing security measures on the communication devices used for online sessions.
7. The participant agrees to create a safety plan (e.g., at least one emergency contact, sharing contact information of the primary physician and the nearest emergency medical facility) in case of a critical situation.

8. The participant agrees that, if necessary, the research staff may provide information to the emergency contact or medical institution in case of acute illness or other emergencies.
9. The participant can prepare a quiet, private space at home free from distractions for conducting the online sessions.
10. The participant agrees to consult with their primary physician if the research staff recommends doing so.
11. The participant agrees in advance that the online session may be interrupted if the research staff, in their professional judgment, determines that continuing the session is not appropriate.
12. The participant agrees not to share information about the study accounts with third parties unrelated to the study, such as emergency contacts.
13. A separate consent process will be conducted for the online sessions, and the participant's agreement to the details described in the consent document will be obtained.

(3) オンラインセッション実施の手順

①対面での実施と同様に、予め日時を決めて行う
②オンライン実施時には、研究スタッフが認知行動療法センターに待機し、緊急時の対応で電子カルテへの記録ができる体制をとる
③研究参加者は、自宅の、気が散ることのない静かでプライベートな空間にて実施する
④対応が必要となる万一の場合に備え、平日の午前中に実施することを原則とする
⑤可能であれば、別室において同居人に待機してもらい、緊急時に対応をサポートしてもらう
⑥予約時刻に、研究スタッフ側からオンラインセッションを開始する
⑦最初に本人確認を行う
⑧毎回のオンライン実施の冒頭に、研究参加者の精神状態と、通信状況からオンラインでの実施可能性を確認する
⑨技術的に問題が生じた場合には、予め確認しておいた研究参加者の電話番号に研究スタッフ側が電話し、状況を確認する
⑩急病急変の事態が生じた場合は、予め同意していた安全計画に基づき、緊急連絡先への連絡や、かかりつけもしくは救急医療機関への受診をしていただく。その際、必要性が認められれば、研究スタッフから緊急連絡先や医療機関への情報提供を行う

(3) Procedures for Conducting Online Sessions:

1. The session date and time will be scheduled in advance, just as for face-to-face sessions.
2. During online sessions, research staff will remain on standby at the Center for Cognitive Behavioral Therapy to ensure that records can be entered into electronic medical records if an emergency arises.
3. The participant will conduct the online session from a quiet, private space at home free from distractions.

4. Online sessions will, in principle, be conducted on weekday mornings to ensure that support can be provided in case of emergencies.
5. If possible, a co-resident should wait in a separate room during the session to assist in an emergency.
6. The research staff will initiate the online session at the scheduled time.
7. Identity verification will be conducted at the beginning of the session.
8. At the start of each online session, the research staff will assess the participant's mental state and the feasibility of conducting the session based on the communication conditions.
9. If technical issues arise, the research staff will call the participant at the pre-confirmed phone number to check the situation.
10. In the event of an acute illness or emergency, the participant will seek treatment at the designated emergency contact or primary care physician based on the agreed-upon safety plan. If necessary, the research staff will provide information to the emergency contact or medical institution.

Strategy for improve adherence and procedures for monitoring adherence

(1)スーパービジョン

CPT の実施にあたっては、被験者の同意のもと、CPT の面接内容をビデオ及び IC レコーダーで録画・録音する。これはスーパーバイザーによる CPT 実施者のスーパービジョンや訓練に使用される。スーパービジョンでは、CPT の臨床研究のために米国で作成された遵守尺度に基づき遵守度及び質の評価を行い、CPT の適正性を担保する。スーパービジョンには研究責任者、CPT 実施者以外にも、研究責任者が認めた研究関係者も陪席することができる。スーパービジョンに参加する全ての者は、面接内容に関し職務規範にて守秘義務が課せられる。なお、面接の録画・録音データは、記憶媒体への保存時は、パスワードをかけ、個人情報管理者のもと厳重に管理される。録画・録音データは、研究チーム内の第三者による治療遵守評価のため、研究終了時まで保存され、その後、完全に消去される。本研究のスーパーバイザーは下記の通りであり、全員が Patricia A. Resick 博士による Consultation Workshop を 2 度受講し、ケースコンサルテーションのための訓練を 48 時間受けている。可能な場合、一部の事例については CPT 熟練治療者（開発者の Resick 博士など）によるコンサルテーションを受ける。

伊藤正哉, PhD. 臨床心理士

蟹江絢子, Ph.D. 医師

Strategy for improve adherence and procedures for monitoring adherence

(1)Supervision:

- With the participant's consent, the contents of CPT sessions will be recorded on video and audio using a video camera and IC recorder. These recordings will be used for supervision and training by the supervisor. The supervision will evaluate adherence and quality based on the adherence scales

created for clinical research in the U.S., ensuring the appropriateness of CPT. In addition to the principal investigator and the CPT implementer, other research team members approved by the principal investigator may also participate in the supervision. All participants in the supervision will be bound by confidentiality obligations regarding the session contents according to their professional code of conduct. The video and audio recordings will be stored on a secure medium with a password and strictly managed by the personal information manager. These recordings will be retained until the study is completed for evaluation by third-party reviewers and will be completely deleted thereafter. The supervisors in this study are as follows, all of whom have attended Dr. Patricia A. Resick's Consultation Workshop twice and received 48 hours of training in case consultation. Where possible, consultation for some cases may be provided by skilled CPT therapists (e.g., Dr. Resick, the developer of CPT).

Masaya Ito, Ph.D., Clinical Psychologist
Ayako Kanie, Ph.D., Physician

(2) CPT 遵守のモニタリング

各事例についてランダムに抽出した 5 分の 1 回程度のセッションについて、研究チーム内の第三者（2 名以上）が遵守尺度（Therapist Adherence Rating Scale）により評価を行う。信頼性検討のために、級内相関係数を算出する。

(2) Monitoring CPT Adherence:

For each case, about one in five sessions will be randomly selected for evaluation by two or more third-party members of the research team using the Therapist Adherence Rating Scale. Intraclass correlation coefficients will be calculated to assess reliability.

(3) 通常治療のモニタリング

通常治療に関しては、中間評価において薬物療法等の通常治療の変更について患者に尋ねるとともに、可能な事例については、随時カルテの記載を参照し、CRF に記録する。

(3) Monitoring Treatment-As-Usual:

Regarding Treatment-As-Usual, patients will be asked about any changes in pharmacotherapy or other aspects of Treatment-As-Usual during the mid-term evaluation. When possible, the content will be confirmed by reviewing the medical records and recorded in the CRF.

（6）試験薬の用法・用量、投与方法又は試験機器の適用方法

該当なし。

(6) Administration and Dosage of Investigational Drugs or Use of Study Equipment:

Not applicable.

（7）併用薬・併用療法についての規定

併用禁止療法：下記の介入は、介入期間中は禁止とする。

他の構造化された精神療法（認知行動療法、EMDR、精神力動療法など）
電気けいれん療法
【設定根拠】
有効性の評価上重大な影響を及ぼすと考えられるため。

併用制限薬：
認知行動療法との併用により有害事象の発生確率を高める薬物は報告されていないため、特定の制限は設けない。

(7) Provisions for Concomitant Medications and Therapies:

Prohibited Concomitant Therapies: The following interventions are prohibited during the intervention period:

- Other structured psychotherapies (Cognitive Behavioral Therapy, EMDR, psychodynamic therapy, etc.)
- Electroconvulsive therapy

Rationale for the Prohibition: These interventions are expected to have a significant impact on the evaluation of effectiveness.

Concomitant Medications: No specific restrictions will be imposed since no medications have been reported to increase the likelihood of adverse events in combination with Cognitive Behavioral Therapy.

（8）評価項目、評価方法

(8) Assessment Items and Evaluation Methods:

Outcomes

(1) 主要評価項目
ベースライン、8 週、17 週で測定される、17 週にかけての CAPS-5 で測定される心的外傷後ストレス症状

Specific measurement variable; CAPS-5
Participant-level analysis metric; Slope over the period of 17 week
Method of aggregation; Mean
Specific measurement time point of interest of analysis; 17 weeks

Outcomes:

Primary Outcome:

- The primary outcome is the change in PTSD symptoms measured by the CAPS-5 over 17 weeks, with assessments conducted at baseline, Week 8, and Week 17.
  - **Specific measurement variable:** CAPS-5
  - **Participant-level analysis metric:** Slope over the period of 17 weeks
  - **Method of aggregation:** Mean

- **Specific measurement time point of interest for analysis:** 17 weeks

【設定根拠】

CAPS-5 は PTSD の重症度を測定するゴールドスタンダードである。被験治療である認知処理療法は 12 回のセッションから構成され、16 週の期間に実施される。CAPS-5 は過去 1 ヶ月の症状を尋ねるために、毎週の CPT 実施が可能で 12 週で介入が終了すれば、ちょうど治療終了後 1 ヶ月の時点が 17 週となる。ただし、介入期間は 16 週であるため、16 週まで介入が続いていることも想定される。その場合であっても、17 週を主要評価項目の測定時点とする。

Rationale for Outcome Selection:

CAPS-5 is the gold standard for assessing PTSD severity. The experimental treatment, CPT, consists of 12 sessions conducted over 16 weeks. CAPS-5 evaluates symptoms over the past month, so the 17-week time point is appropriate for assessing symptoms one month after the completion of the 12-week CPT intervention. Even if the intervention continues up to 16 weeks, the 17-week time point will be used as the primary outcome measurement.

(2) 副次的評価項目

(2) Secondary Outcomes:

有効性評価：

1. 17 週の自覚的心的外傷後ストレス症状の重症度（PTSD Check List for DSM-5; PCL-5）
2. 17 週の治療反応割合（様々な指標を報告）

Efficacy Evaluation:

1. Self-reported PTSD symptom severity at 17 weeks (PCL-5)
2. Treatment response rate at 17 weeks (reported using various indicators)

安全性評価：

1. 有害事象の発生（口渇、便秘、排尿障害、視力調節障害、起立性低血圧、眠気、倦怠感、不眠、不安・焦燥、落ち込み・意欲低下、食欲不振、体重増加、体重減少、性欲低下、動悸、ふるえ、発汗、頭痛、ふらつき、その他）

Safety Evaluation:

1. Occurrence of adverse events such as dry mouth, constipation, urinary retention, visual accommodation disorder, orthostatic hypotension, drowsiness, fatigue, insomnia, anxiety, depression, loss of appetite, weight gain, weight loss, decreased libido, palpitations, tremors, sweating, headache, dizziness, etc.

【設定根拠】

副次評価項目として、自覚的心的外傷後ストレス症状を測定する PCL-5 を用いる。PCL-5 は DSM-5 の診断基準に合致した項目からなる自己記入式尺度である。治療反応割合についてのゴールドスタンダードは存在しない。そこで、Loerincet al. [7]が推奨する 5 項目を考慮する。すなわち、 (1) Clinically significant change index を算出し、(2) ITT 解析を行い、[8] 独立評価者による評価を用い、(4) 多面的な指標を利用し、(5) モダリティの異なる指標を用いる。本臨床試験で言えば、(1) Clinically significant change index は臨床試験終了時に CAPS-5 や PCL-5 の臨床群及び健常群のデータが公表されていれば算出可能である、(2) ITT 解析は可能である、[8] CAPS-5 は独立評価である、(4) および(5) CAPS-5、PCL-5、CAPS-5 内に含まれる Clinical Global Impression 評定といった多面的かつモダリティの異なる評価を利用できる。そこで、本研究では、Clinically significant change index (利用可能なデータがある場合)、Reliable change index、CAPS-5 内の CGI-I 評価、CAPS-5 による 30%以上の得点減少、CAPS-5 及び PCL-5 によるカットオフ値以下、CAPS-5 および PCL-5 による診断の喪失そしてこれらの組み合わせについて、論文公表時に、治療反応割合として最も信頼性が高いとされる指標を公表する。もし治療反応割合についてコンセンサスが認められない場合は、全ての指標を報告する。

さらに、治療の安全性を評価するために有害事象の発生有無についても副次評価項目とした。認知処理療法は侵襲的な介入を伴わず、本研究の介入からは健康への有害事象は想定されない。ただし、被験治療（認知処理療法）は、患者がそれまで回避してきたトラウマの記憶や考えに敢えて取り組む治療である。その過程で、不安に関連する身体症状（動悸、ふるえ、発汗、頭痛、ふらつき等）や精神症状（不安、焦燥）が一時的に悪化することもある。これらについて治療期間を通して評価する。

Rationale for Secondary Outcome Selection:

PCL-5, a self-report measure that aligns with DSM-5 diagnostic criteria, will be used as a secondary outcome to measure PTSD symptoms. The gold standard for treatment response is not established. Therefore, the five indicators recommended by Loerinc et al. (e.g., Clinically significant change index, ITT analysis, independent evaluator assessment) will be considered in this study. Safety will be evaluated based on the occurrence of adverse events, with CPT expected to temporarily exacerbate anxiety-related symptoms as patients confront trauma-related memories and thoughts.

(4) その他の主な評価項目

(4) Other Key Outcome Measures:

その他の有効性評価：

自記式質問票による患者評価 – 他の成果指標：

1. 自覚的うつ症状の重症度 (Patient Health Questionnaire-9; PHQ-9)
2. 自覚的生活の質の程度(EQ-5D-5L)
3. 自覚的機能障害の程度(Sheehan Disability Scale; SDS)

4. 自覚的解離症状（CAPS-5、PCL-5 に追加した項目）
5. 自殺念慮（Suicidal Ideation Attributes Scale; SIDAS）

Other Efficacy Evaluations:

- Patient evaluations through self-report questionnaires, including:
    1. Severity of depressive symptoms (PHQ-9)
    2. Quality of life (EQ-5D-5L)
    3. Functional impairment (Sheehan Disability Scale; SDS)
    4. Dissociative symptoms (additional items to CAPS-5 and PCL-5)
    5. Suicidal ideation (Suicidal Ideation Attributes Scale; SIDAS)

治療メカニズムやプロセスに関する評価：

自記式質問票による患者評価 – 治療メカニズムの評価：

6. 心的外傷後不適応的信念（Posttraumatic Maladaptive Belief Scale; PMBS）
7. トラウマ関連の自責感（Trauma-Related Guilt Inventory; TRGI）
8. 感情調整（Emotion Regulation Questionnaire; ERQ）

Evaluation of Treatment Mechanisms and Processes:

Patient evaluation of treatment mechanisms through self-report questionnaires, including:

6. Post-traumatic maladaptive beliefs (Posttraumatic Maladaptive Belief Scale; PMBS)
7. Trauma-related guilt (Trauma-Related Guilt Inventory; TRGI)
8. Emotional regulation (Emotion Regulation Questionnaire; ERQ)

治療プロセスの評価：

9. 治療同盟（Session Rating Scale; SRS）—治療者・患者評価
10. 宿題遵守—治療者評価
11. 治療遵守（Treatment Adherence and Competence Protocol; TACP） –第三者評価

Treatment Process Evaluation:

9. Evaluation of the therapeutic alliance using the Session Rating Scale (SRS)—assessed by both therapist and patient
10. Homework adherence—assessed by the therapist
11. Treatment adherence (Therapist Adherence and Competence Protocol; TACP)—assessed by third-party reviewers

独立評定者の盲検化チェック：

12. 独立評価者のための治療知識用紙（Independent Evaluator Knowledge of Outcome; IEKNO）

Blindness Check for Independent Evaluators:

12. Independent Evaluator Knowledge of Outcome (IEKNO) will be used to check the effectiveness of blinding.

生物学的側面の変容に関する評価：

脳画像データによる評価：

13. 3D-T1 強調像
14. T2 強調像
15. 高解像度 T2 強調像
16. fluid attenuated inversion recovery（FLAIR）
17. arterial spin labeling（ASL）
18. 拡散テンソル像（DTI）

Biological Outcome Measures:

- Brain imaging data, including:
  13. 3D-T1-weighted images
  14. T2-weighted images
  15. High-resolution T2-weighted images
  16. Fluid-attenuated inversion recovery (FLAIR) images
  17. Arterial spin labeling (ASL)
  18. Diffusion tensor imaging (DTI)

【設定根拠】

患者本人の主観的改善度も重要な指標であるために、患者自身の評定を評価項目とする。自己記入式尺度として、心的外傷後ストレス症状（PCL-5）、うつ（PHQ-9）、生活の質（EQ-5D-5L）、機能障害（SDS）、解離症状（PCL-5 追加項目）、自殺念慮（SIDAS）といった多面的なアウトカム評価を行う。

本研究で取り上げる認知処理療法は、トラウマに関連する認知を再構成することによって、症状の緩和を図る。そのため、トラウマに関連した認知においても実際に変化が起こっているかを検討することは重要である。そこで、トラウマ後の不適応的な信念を測定する PMBS、自責感を測定する TRGI、認知再評価及び感情表出抑制による感情調整を測定する ERQ によりこれらの変化を検討する。

精神療法の効果は、治療への信頼や期待、介入する治療者との関係性や、介入の適切な実施に依存する部分がある。そこで、治療者−患者関係の質を評価するセッション評価尺度（SRS）、宿題遵守、治療遵守尺度（TACP）を用いて治療プロセスを評価する。宿題遵守・治療遵守の評価は、介入群のみにおいて実施する。SRS は治療者と患者が評価をし、宿題遵守は治療者のみが評価する。TACP は第三者による評価であり、各事例につき、ランダムに 5 分の 1 のセッションを評価する。

独立評価者の独立性が担保されているかを検討するため、IEKNO を用いて盲検化の確認をする。
精神療法が、患者自身の主観的報告のみならず、より客観的な生物学的指標（脳の構造や機能、ネットワーク）にも変容を促すことが示されている。そこで本研究では、3D-T1 強調像、T2 強調像、高解像度 T2 強調像、fluid attenuated inversion recovery（FLAIR）、arterial spin labeling（ASL）、拡散テンソル像（DTI）を撮像し、介入前後の脳構造および脳機能を評価する。なお、以下の除外基準を満たす患者については、脳画像データの測定は行わない。

Rationale for Setting

Since the subjective improvement level of patients is an important indicator, the patients' self-assessment will be used as evaluation items. Various outcome measures will be used, including: Post-Traumatic Stress Symptoms (PCL-5), Depression (PHQ-9), Quality of Life (EQ-5D-5L), Functional Impairment (SDS), Dissociative Symptoms (Additional Items in PCL-5), and Suicidal Ideation (SIDAS).

Cognitive Processing Therapy (CPT), which is the focus of this study, aims to alleviate symptoms by restructuring trauma-related cognition. Therefore, it is important to examine whether actual changes occur in trauma-related cognition. For this purpose, changes will be assessed using:

- **Post-Traumatic Belief Scale (PMBS)** to measure maladaptive beliefs following trauma
- **Trauma-Related Guilt Inventory (TRGI)** to measure guilt
- **Emotion Regulation Questionnaire (ERQ)** to assess emotion regulation through cognitive reappraisal and suppression of emotional expression

The effectiveness of psychotherapy depends on factors such as trust and expectations regarding the treatment, the relationship with the therapist, and the proper implementation of the intervention. To evaluate the treatment process, the study will use:

- **Session Rating Scale (SRS)** for assessing the quality of the therapist-patient relationship
- **Homework Compliance and Treatment Adherence Scale (TACP)**

Homework compliance and treatment adherence will be assessed only in the intervention group. SRS will be evaluated by both the therapist and the patient, while homework compliance will be assessed only by the therapist. TACP will be evaluated by a third party, with one-fifth of the sessions randomly selected for assessment.

To ensure the independence of evaluators, blinding will be confirmed using the **Independent Evaluation Knowledge and Note Observer (IEKNO)**.

It has been demonstrated that psychotherapy can induce changes not only in patients' subjective reports but also in more objective biological indicators (brain structure, function, and networks). Therefore, this study will use:

- **3D-T1-weighted images**
- **T2-weighted images**
- **High-resolution T2-weighted images**
- **Fluid-Attenuated Inversion Recovery (FLAIR)**
- **Arterial Spin Labeling (ASL)**
- **Diffusion Tensor Imaging (DTI)**

To evaluate brain structure and function before and after the intervention. Patients who meet the following exclusion criteria will not have brain imaging data measured.

【脳画像データ測定に関する除外基準】
1. 心臓ペースメーカーなど、体内に金属製の埋め込み物がある者
2. 閉所恐怖の症状などを有する者
3. 妊娠中および妊娠の可能性がある者
4. 課題の遂行が困難なほどの精神疾患、もしくは重篤な身体疾患に罹患している者、計算能力などの一般的な認知機能に支障をきたしている者
5. その他、研究者が不適当と判断した者

Exclusion Criteria for Brain Imaging Data:

1. Patients with metal implants such as pacemakers
2. Patients with symptoms of claustrophobia
3. Pregnant patients or those who may be pregnant
4. Patients with severe psychiatric or physical disorders or cognitive impairment affecting the execution of tasks
5. Patients deemed inappropriate by the researcher

（9）観察及び検査項目

(9)　Data Collection and Assessment Items

Data collection methods

Plans for assessment and collection of trial data and description of study instruments

(1) 面接評価の手法と評価者

本研究では、被験者への CPT 担当者ではない独立した評価者が評価を実施する。独立評価者は、患者の割付を知りうるあらゆる情報や打ち合わせへの接触が禁じられる。独立評価者は中間と介入後評価後に、独立評価者のための治療知識用紙（IEKNO）に回答する。

(1)Data Collection Methods and evaluators:
The research will employ structured interviews and evaluations conducted by independent assessors who are not involved in providing the Cognitive Processing Therapy (CPT) to the participants. These assessors are prohibited from accessing any information or discussions that could reveal the participants' group assignment. Independent evaluators will also complete the Independent Evaluator Knowledge of Outcome (IEKNO) form after mid-treatment and post-treatment assessments.

評価者：
介入に対して盲検化された者を独立評価者とする。評価者には、CAPS-5 について合計 4 時間以上の研修と、CAPS あるいは CAPS-5 について 3 例以上の実施経験もしくはそれと同等の経験が求められる。この評価者によって評価された結果を主要評価項目として使用する。

Evaluators:
Independent assessors will conduct the evaluations, and the primary outcome will be based on their assessments. Evaluators must undergo a minimum of 4 hours of training in the Clinician-Administered PTSD Scale for DSM-5 (CAPS-5) and have experience conducting at least three assessments or equivalent experience. The results of these evaluations will be used as the primary outcome measure.

測定方法：
所定の用紙を用いて実施する。評価はすべて IC レコーダーにより録音され、上記の盲検化された独立評価者とは別の第三者の評価者による評価を通した信頼性検討に用いる。信頼性は、全評価の 20%（全症例を実施した場合は合計 174 評価中の 35 評価となる）について、級内相関係数を算出して検討する。

Measurement Method:
All assessments will be conducted using the designated forms. The evaluations will be recorded on an IC recorder and used to assess reliability by a third-party evaluator who is blinded to the participants' group assignments. Reliability will be assessed by calculating the intraclass correlation coefficient (ICC) on 20% of all assessments (35 out of 174 total evaluations if all cases are completed).

(2) 観察および検査項目
(2) Observation and Testing Items:

1. 患者背景の基礎情報
   治療開始前に、次の項目について調査を行う。
   担当治療者名、性別、生年月日、婚姻状況、年収、就学・就労状況、病名（主治医による DSM-5 診断）、主訴、登録以前までの治療歴（心理相談含む）、精神科既往歴、教育歴、家族歴、飲酒・喫煙
2. 宿題遵守：認知処理療法では、治療の一環として宿題の実施遵守を評価する。
3. 併用治療の遵守状況：Visit 毎に患者に確認するとともに、可能な場合はカルテ記載を随時確認することにより、薬物療法の安定性および併用禁止治療の有無を確認する。

4. 心的外傷後ストレス障害の診断及び重症度：CAPS、PCL-5 plus
5. 精神疾患の有無：MINI（除外基準、併存疾患の確認）、主治医による診断
6. 抑うつ症状：PHQ-9
7. 生活の質および機能障害： EQ-5D-5L、SDS
8. 治療メカニズム：PMBS、TRGI、ERQ
9. 治療プロセス：SRS、HC、TACP
10. 有害事象の確認：Visit 毎に口頭および所定の用紙にて確認する。
11. 脳画像データ：3D-T1 強調像、T2 強調像、高解像度 T2 強調像、FLAIR、ASL、DTI

1. **Basic Participant Information:** Before starting the treatment, the following participant information will be collected:Name of the treating physician, gender, date of birth, marital status, annual income, education/employment status, diagnosis (DSM-5 diagnosis by the primary physician), chief complaints, previous treatment history (including psychological counseling), psychiatric history, educational background, family history, drinking/smoking habits.
2. **Homework Adherence:** Adherence to homework assignments will be evaluated as part of the CPT process.
3. **Compliance with Concomitant Treatments:** At each visit, patients will be asked to confirm their compliance with pharmacotherapy and other treatments. When possible, medical records will also be reviewed to assess the stability of pharmacotherapy and the absence of prohibited concomitant treatments.
4. **PTSD Diagnosis and Severity:** Diagnosis and severity of PTSD will be assessed using CAPS-5 and PCL-5 plus additional measures.
5. **Presence of Other Psychiatric Disorders:** The Mini International Neuropsychiatric Interview (MINI) will be used to assess the presence of other psychiatric disorders, verify exclusion criteria, and confirm comorbidities.
6. **Depressive Symptoms:** Depressive symptoms will be assessed using the Patient Health Questionnaire-9 (PHQ-9).
7. **Quality of Life and Functional Impairment:** Quality of life will be assessed using the EQ-5D-5L, and functional impairment will be evaluated using the Sheehan Disability Scale (SDS).
8. **Treatment Mechanisms:** The Posttraumatic Maladaptive Belief Scale (PMBS), Trauma-Related Guilt Inventory (TRGI), and Emotion Regulation Questionnaire (ERQ) will be used to assess changes in treatment mechanisms.
9. **Treatment Process:** The therapeutic alliance will be evaluated using the Session Rating Scale (SRS) from both the therapist's and the participant's perspectives. Homework adherence will be assessed by the therapist, and treatment adherence will be evaluated by third-party reviewers using the Therapist Adherence and Competence Protocol (TACP).
10. **Adverse Events:** Adverse events will be checked at each visit, both orally and using the designated forms.
11. **Brain Imaging Data:** Imaging data will be collected, including 3D-T1-weighted images, T2-weighted images, high-resolution T2-weighted images, fluid-attenuated inversion recovery (FLAIR) images, arterial spin labeling (ASL), and diffusion tensor imaging (DTI).

(3) 調査スケジュール

(3) Assessment Schedule:

上記の項目は調査スケジュール表（Figure 2）に基づき、試験施設にて検査する。調査結果を CRF に記載する。規定された観察日（検査日）のずれの許容範囲は±14 日とする。

The above items will be assessed according to the assessment schedule table (Figure 2) at the study site. The results of the assessments will be recorded in the Case Report Form (CRF). The allowable range for deviations in the scheduled observation (assessment) dates is ±14 days.

（10）実施する検査について

(10) Procedures for Conducting Tests

測度の説明

Description of measurements

評価者評価

Assessment by independent evaluators

1. PTSD 診断及び重症度（Clinician Administered PTSD Scale for DSM-5 ; CAPS-5）：

　CAPS-5 は、心的外傷後ストレス障害の DSM-5 診断および症状評価のための構造化面接法であり、PTSD 評価のゴールドスタンダードである[9]。CAPS は 30 項目から構成され、バージョンによって PTSD の現在診断（過去 1 ヶ月）と生涯診断、過去 1 週間の PTSD 症状を評価できる。内容としては、PTSD 症状の 20 項目のほかに、発症時期と症状継続期間、主観的苦痛、社会・職業機能への症状の影響、前回の CAPS 施行からの症状改善、回答の妥当性、全般的 PTSD 重症度、解離サブタイプ（離人感・現実感消失）を評価する。CAPS は、聴取する期間に応じて 3 つのバージョンがある（過去 1 週間、過去 1 ヶ月、最も症状が重かった時期）。本研究では、診断および重症度の双方を評価する必要があるために、過去 1 ヶ月版を用いる。PTSD 症状は、20 項目について 0–4 の 5 件法（0; なし、1; 軽度・閾値下、2; 中等度・閾値、3; 重度・顕著に悪化、4; 極重度・不能状態）で回答する。得点範囲は 0–80 点であり、得点が高いほど PTSD 症状が重度であることを示す。また、症状クラスター（再体験、回避、認知と気分の否定的変化、過覚醒、解離）ごとに得点化を行う。PTSD 診断は、各症状の項目が 2 以上と評価されれば、症状ありとみなされる。一部の項目（認知と気分の否定的変化と過覚醒について問う項目 9 および 11–20）については、トラウマ体験との関連性の評価も求められる。これらの評価および基準 G/F の評価について、DSM-5 診断基準に基づいて、診断を行う（B・C 基準それぞれで一つ以上、D・E 基準それぞれで二つ以上の該当、基準 F：一ヶ月以上の症状持続、基準 G：項目 23–25 で 2 点以上で評価される臨床的に有意な苦痛や機能障害の存在）。全施行時間は 45–60 分である。DSM-5 の出版に合わせて改定されたため、現在（2015 年 11 月 11 日）まで信頼性と妥当性の報告はない。なお、トラウマティックな出来事の確認として、CAPS-5 と併用して用いられるライフイベンツチェクリスト（Life Events Checklist; LEC）を用いる。

1. PTSD Diagnosis and Severity (Clinician-Administered PTSD Scale for DSM-5; CAPS-5):

CAPS-5 is a structured interview used for diagnosing and evaluating the symptoms of Post-Traumatic Stress Disorder (PTSD) based on the DSM-5 criteria, and it is considered the gold standard for PTSD assessment. CAPS consists of 30 items and, depending on the version, can evaluate the current PTSD diagnosis (within the past month), lifetime diagnosis, and PTSD symptoms over the past week. In addition to the 20 items assessing PTSD symptoms, the CAPS

also evaluates the onset and duration of symptoms, subjective distress, the impact of symptoms on social and occupational functioning, symptom improvement since the last CAPS administration, the validity of responses, overall PTSD severity, and the dissociative subtype (depersonalization and derealization).
CAPS has three versions tailored to different timeframes for evaluation (the past week, the past month, and the worst period of symptoms). For this study, the version evaluating symptoms over the past month will be used, as both diagnosis and severity need to be assessed. PTSD symptoms are rated on a 5-point scale (0–4) where 0 indicates "absent," 1 "mild/subthreshold," 2 "moderate/threshold," 3 "severe/markedly elevated," and 4 "extreme/incapacitating." The total score ranges from 0 to 80, with higher scores indicating more severe PTSD symptoms. Scores are also calculated for each symptom cluster (re-experiencing, avoidance, negative alterations in cognition and mood, hyperarousal, dissociation).
A PTSD diagnosis is made if a score of 2 or higher is given on the relevant symptom items. For some items (items 9 and 11–20, which assess negative alterations in cognition and mood, and hyperarousal), the evaluation must also consider the relationship to the traumatic experience. Diagnosis is based on the DSM-5 criteria, requiring at least one symptom from criteria B and C, at least two symptoms from criteria D and E, persistence of symptoms for more than one month (criterion F), and the presence of clinically significant distress or functional impairment, as indicated by a score of 2 or more on items 23–25 (criterion G). The total administration time for the CAPS-5 is 45–60 minutes. Since CAPS-5 was revised in conjunction with the publication of DSM-5, reliability and validity reports are not yet available as of November 11, 2015. Additionally, the Life Events Checklist (LEC) is used in conjunction with CAPS-5 to confirm traumatic events.

2. 精神疾患の診断（Mini International Neuropsychiatric Interview adapted to derive DSM-5 diagnosis; MINI）：
   MINI は、DSM-5 の主要な精神疾患や臨床状態を診断するための簡易構造化面接法である。本研究では、DSM-5 診断を評価するために最新版の MINI 7.0.0 を使用する。評価項目は、抑うつエピソード、うつ病/大うつ病性障害、自殺念慮、自傷及び自殺行動、自殺行動障害、躁病エピソード、軽躁病エピソード、双極 I 型障害、双極 II 型障害、双極性障害、特定不能のもの、精神病性の特徴を伴う双極 I 型障害、パニック症/パニック障害、広場恐怖症、社交不安症/社会不安障害（社交恐怖）、強迫症/強迫性障害、心的外傷後ストレス障害、アルコール使用障害、物質使用障害（非アルコール）、精神病性障害、精神病性の特徴を伴う気分障害、神経性やせ症/神経性無食欲症、神経性過食症/神経性大食症、過食性障害、全般性不安症/全般性不安障害、医学的、器質的および薬物関連の病因を除外、反社会性パーソナリティ障害であり、各診断基準について「はい」「いいえ」で回答する。短時間で施行可能である（18.7±11.6 分、中央値 15 分）[10]。旧版については、日本版についても信頼性と妥当性が確認されている[11]。妥当性については、Structured Clinical Interview for Diagnostic and Statistical Manual-III-R-patient version との基準関連妥当性が示されており（Kappa > .49）、信頼性については高い評者間一致度が報告されている（Kappa > .72）。

2. Diagnosis of Mental Disorders (Mini International Neuropsychiatric Interview adapted to derive DSM-5 diagnosis; MINI):
The MINI is a brief structured interview used to diagnose major mental disorders and clinical conditions according to DSM-5 criteria. In this study, the latest version, MINI 7.0.0, will be used to evaluate DSM-5 diagnoses. The assessment includes items for depressive episodes, depression/major depressive disorder, suicidal ideation, self-harm and suicidal behaviors, suicide

behavior disorder, manic episodes, hypomanic episodes, bipolar I disorder, bipolar II disorder, unspecified bipolar disorder, bipolar I disorder with psychotic features, panic disorder, agoraphobia, social anxiety disorder/social phobia, obsessive-compulsive disorder, post-traumatic stress disorder, alcohol use disorder, substance use disorder (non-alcohol), psychotic disorders, mood disorders with psychotic features, anorexia nervosa, bulimia nervosa, binge eating disorder, generalized anxiety disorder, exclusion of medical, organic, and substance-related causes, and antisocial personality disorder. Each diagnostic criterion is answered with a "yes" or "no." The interview can be administered in a short amount of time (18.7±11.6 minutes, median 15 minutes).
For the previous version, reliability and validity have been confirmed for the Japanese version as well. The validity of the MINI is demonstrated by its criterion-related validity with the Structured Clinical Interview for DSM-III-R-patient version (Kappa > .49), and it has a high inter-rater reliability (Kappa > .72).

3. 盲検化の評価（Independent Evaluator Knowledge of Outcome; IEKNO）：
　本尺度は、パニック障害に対する大規模な臨床研究[12]や、統一プロトコルの RCT（Farchione et al., 2012）において利用された尺度である[13]。3 項目からなり、患者がどちらの群に割り付けられたと考えるか、それについてどの程度の自信を持っているか 0 から 8 の Likert で回答を求める。そして、最後の項目では、割付を見抜いたと考えられる場合には、どのような情報からそう考えられるかを尋ねる。本研究では、Blinding Index[14]を算出するために、割付の推測について“介入群”、“対照群”、“わからない”の 3 つから選択する回答方式とする。

3. Evaluation of Blinding (Independent Evaluator Knowledge of Outcome; IEKNO):
This scale has been used in large-scale clinical studies on panic disorder and in randomized controlled trials (RCTs) of unified protocols (Farchione et al., 2012). The scale consists of three items. It asks the evaluator to indicate, on a Likert scale from 0 to 8, which group they believe the patient has been assigned to and how confident they are in that belief. In the final item, if the evaluator believes they have uncovered the group assignment, they are asked to describe the information that led them to that conclusion. In this study, the response format will be modified to allow the evaluator to choose from three options—"intervention group," "control group," or "don't know"—in order to calculate the Blinding Index.

自記式評価
Self-report measures

4. PTSD スクリーニング尺度（PC-PTSD）：
　プライマリ・ケアのための PTSD スクリーニング尺度(DSM-5 版)(PC-PTSD-5)は、PTSD の疑い (probable PTSD)がある人を同定するための 5 項目のスクリーニング尺度である[15]。この尺度では、トラウマティックな出来事を経験したことがあるかを最初に回答してもらう。もし経験がなければ、PC-PTSD-5 得点は 0 点となり、終了となる。日本版は申請者らによって翻訳されているが、信頼性や妥当性に関するデータはない。

4. PTSD Screening Scale (PC-PTSD):
The Primary Care PTSD Screen for DSM-5 (PC-PTSD-5) is a five-item screening tool designed to identify individuals who may have probable PTSD. The scale first asks whether the individual

has experienced a traumatic event. If the individual has not experienced a traumatic event, their PC-PTSD-5 score is 0, and the screening ends. The Japanese version of this scale has been translated by the applicants, but there is currently no data on its reliability or validity.

5. PTSD 症状（PTSD Checklist for DSM-5 ; PCL-5）：

PCL-5 は DSM-5 の PTSD 診断基準に対応した 20 項目から構成される自己記入式尺度である[1]。PCL-5 は、治療中やその後の症状変化のモニタリング、PTSD 患者のスクリーニング、暫定的な PTSD 診断のために利用できる。回答は、0–4 で行う（0; 全くない、1; 少し、2; 中程度、3; かなり、4; 非常に）。得点範囲は 0–80 点であり、得点が高いほど PTSD 症状が重篤であることを示す。回答時間は 5–10 分程度である。信頼性と妥当性については、現在米国で検討中である。暫定的な値ではあるが、38 点以上が PTSD 診断のカットオフ値として推奨されている[1]。日本版は我々の研究チームがバックトランスレーション手続きを経て、前バージョンの PCL-S を改定する形で作成している。本研究では、原版の 20 項目に加えて、罪責感、注意の減退、非現実感、離人感、悪夢を測定する項目を追加し、PCL-5 plus と呼ぶ。2016 年度中には、インターネットを通した調査により、PCL-5 plus の日本人における本尺度の信頼性と妥当性を検討した論文を公表する予定である。

5. PTSD Symptoms (PTSD Checklist for DSM-5; PCL-5):

The PCL-5 is a self-report scale consisting of 20 items that correspond to the DSM-5 diagnostic criteria for PTSD. The PCL-5 can be used to monitor symptom changes during and after treatment, screen for PTSD, and make a provisional PTSD diagnosis. Responses are rated on a scale from 0 to 4 (0: Not at all, 1: A little bit, 2: Moderately, 3: Quite a bit, 4: Extremely). The total score ranges from 0 to 80, with higher scores indicating more severe PTSD symptoms. The questionnaire takes about 5 to 10 minutes to complete. The reliability and validity of the PCL-5 are currently being evaluated in the United States. Although the cutoff score is provisional, a score of 38 or higher is recommended for a PTSD diagnosis. The Japanese version was created by our research team through a back-translation process, revising the previous version, PCL-S. In this study, we use the PCL-5 plus, which includes the original 20 items along with additional items measuring guilt, attentional decline, derealization, depersonalization, and nightmares. We plan to publish a paper during the 2016 fiscal year that examines the reliability and validity of the PCL-5 plus in a Japanese population through an internet-based survey.

6. 抑うつ症状（Patient Health Questionnaire-9 ; PHQ-9）：

PHQ-9 は、過去 2 週間の抑うつ症状の頻度を訪ねる尺度である。0 から 3 で回答される（0.全くない、1.数日、2.半分以上、3.ほとんど毎日）。得点範囲は 0–27 点であり、得点が高いほど抑うつ症状が重篤であることを示す。PHQ-9 は広く疫学調査や臨床研究で使用されているものであり、臨床群および健常者において妥当性が検証されている[16]。日本版においては精神疾患患者 131 名における MINI による診断との一致率を算出しており、大うつ病性障害については Kappa = .79 と報告されている[17]。日本版においては、他にも内科やプライマリケアにおけるカットオフ値を検討する論文が示されており[18] [19, 20]、許容される範囲の感度と特異度を示していることから、妥当性の一部が確認されていると判断できる。本研究では、ベースライン、8 週、17 週、34 週は 2 週間の期間について回答を求め、CPT のための Visit 時での回答の場合には 1 週間の期間について回答を求める。

6. Depressive Symptoms (Patient Health Questionnaire-9; PHQ-9):

The PHQ-9 is a scale that asks about the frequency of depressive symptoms over the past two weeks. Responses are rated on a scale from 0 to 3 (0: Not at all, 1: Several days, 2: More than half the days, 3: Nearly every day). The total score ranges from 0 to 27, with higher scores indicating more severe depressive symptoms. The PHQ-9 is widely used in epidemiological studies and clinical research, and its validity has been confirmed in both clinical populations and healthy individuals. In the Japanese version, the agreement rate with the diagnosis made using the MINI among 131 psychiatric patients was calculated, with a Kappa value of .79 for major depressive disorder. Additionally, several papers have examined the cutoff values for use in internal medicine and primary care settings in Japan, showing acceptable sensitivity and specificity, thereby confirming part of the scale's validity. In this study, participants will be asked to complete the PHQ-9 at baseline, 8 weeks, 17 weeks, and 34 weeks, reflecting the symptoms over the past two weeks. For responses during visits related to Cognitive Processing Therapy (CPT), participants will be asked to reflect on the past week's symptoms.

7. 自殺念慮（Suicidal Ideation Attributes Scale; SIDAS）：
SIDAS は、5 項目からなる自殺念慮の強さを測定する尺度であり、頻度、制御可能性、企図の可能性、苦痛、日常生活への障害について尋ねる。過去一ヶ月について、0-10 の 11 件法で回答するが、項目により評定肢の内容は異なる。頻度で 0 と回答した場合には、他の項目は全て回答をしない。制御可能性のみが逆転項目であり、得点は全項目が足し上げられる。高い得点は強い自殺念慮を意味する。回答時間は 30–60 秒とされる。高い内的整合性（Cronbach α = .91）と、PHQ-9、GAD-7 等との収束的妥当性が確認されている。本尺度は、自殺念慮を測定する様々な尺度の中でも、最も推奨される尺度のひとつとして指摘されている[21]。

7. Suicidal Ideation (Suicidal Ideation Attributes Scale; SIDAS):
The SIDAS is a scale consisting of five items that measure the intensity of suicidal ideation, assessing aspects such as frequency, controllability, likelihood of attempt, distress, and interference with daily life. Participants respond based on their experiences over the past month, using an 11-point scale ranging from 0 to 10, though the specific content of the response options varies depending on the item. If a participant answers 0 for frequency, they do not need to respond to the other items. The controllability item is reverse-scored, and the total score is calculated by summing the scores for all items. A higher score indicates stronger suicidal ideation. The scale takes approximately 30–60 seconds to complete. The SIDAS has been shown to have high internal consistency (Cronbach's α = .91) and demonstrates convergent validity with measures like the PHQ-9 and GAD-7. Among the various scales available for measuring suicidal ideation, the SIDAS is noted as one of the most recommended tools.

8. 生活の質（Euro-Qol; EQ-5D5L）：
EQ-5D-5L は、QOL を測定する選考による尺度である。5 項目（移動の程度、身の回りの管理、ふだんの生活、痛み・不快感、不安・ふさぎ込み）からなり、5 件法で回答する。評定肢は数値によるアンカリングはされておらず、項目ごとに用意された評定肢のそれぞれについている四角のボックスをチェックする形で回答する。また、健康状態全般を Visual Analogue Scale により評価する（0.想像できる最も悪い健康状態、100.想像できる最も良い健康状態）。各国独自に作られたタリフを用いて、効用値を算出する。施行時間 3 分程度である[22, 23]。日本人における標準値[24]、およびタリフ[25]が公表されており、これらの値を用いて、生活の質の程度や医療経済評価について検討する。

8. Quality of Life (Euro-Qol; EQ-5D-5L):

The EQ-5D-5L is a preference-based scale used to measure quality of life (QOL). It consists of five items (mobility, self-care, usual activities, pain/discomfort, anxiety/depression), each of which is answered using a five-level scale. The response options are not numerically anchored; instead, participants check a box corresponding to their level of functioning or discomfort for each item. Additionally, participants evaluate their overall health status using a Visual Analogue Scale (0: worst imaginable health state, 100: best imaginable health state). Utility values are calculated using country-specific tariffs. The assessment takes approximately three minutes to complete. Standard values and tariffs specific to the Japanese population have been published, and these values are used to assess the degree of quality of life and to conduct health economic evaluations.

9.　機能障害（Sheehan Disability Scale; SDS）：　SDS は、三つの領域（職業/学校、社会生活、家族生活）における機能障害を尋ねるものであり、0 から 10 の Visual Analogue Scale である。得点範囲は 0－30 点であり、得点が高いほど機能障害が重篤であることを示す。施行時間は 2 分程度である。様々な Population において信頼性および妥当性が報告されている[26, 27]。日本人患者を対象としたデータにおいては、Cronbach'sα=.84–.87、検査−再検査の総得点における級内相関係数 0.98 と高い信頼性が報告されている。また、妥当性としては Global assessment of functioning との強い負の相関（r = -.83）を報告している。

9. Functional Impairment (Sheehan Disability Scale; SDS):

The SDS is a scale that assesses functional impairment across three domains: work/school, social life, and family life/home responsibilities. Participants rate each domain using a Visual Analogue Scale ranging from 0 to 10. The total score ranges from 0 to 30, with higher scores indicating more severe functional impairment. The scale takes approximately two minutes to complete. Reliability and validity have been reported across various populations. For Japanese patients, the SDS has shown high reliability, with Cronbach's α ranging from .84 to .87, and an intraclass correlation coefficient of 0.98 for total scores on test-retest reliability. Additionally, strong negative correlations with the Global Assessment of Functioning (r = -.83) have been reported, supporting its validity.

10.　心的外傷後不適応的信念（Posttraumatic Maladaptive Beliefs Scale; PMBS）：
PMBS は、トラウマ体験後に抱きやすい認知を測定する尺度である。当尺度は、トラウマ後の認知を測定する様々な既存尺度を概観し、研究でも臨床で利用し易いように考慮した上で作成された尺度である[28]。危害の脅威、自己価値と判断、他者への信頼の 3 項目から構成され、7 件法で回答する（0.全くもって当てはまらない、7.完全に当てはまる）。得点が高さは、不適応的信念の強さを示す。Cronbach's α は全項目で.82、危害の脅威で.76、自己価値と判断で.71、他者への信頼で.71 と報告されている。収束性および弁別的妥当性については、トラウマ後の認知を測定する様々な尺度のほか、PTSD 症状、うつ・不安症状との相関係数の算出により検証されている。日本語版は出版されておらず、当研究チームによりバックトランスレーション手続きを通して日本版を作成する予定である。

10. Posttraumatic Maladaptive Beliefs (Posttraumatic Maladaptive Beliefs Scale; PMBS):

The PMBS is a scale designed to measure cognitions that individuals are prone to develop after experiencing trauma. This scale was created by reviewing various existing scales that measure posttraumatic cognitions and considering ease of use in both research and clinical settings. The PMBS consists of three subscales: threat of harm, self-worth and judgment, and trust in others.

Responses are given on a 7-point Likert scale (0: Not at all, 7: Completely applies). Higher scores indicate stronger maladaptive beliefs. The scale's reliability, as measured by Cronbach's α, is reported as .82 for the overall scale, .76 for the threat of harm, .71 for self-worth and judgment, and .71 for trust in others. Convergent and discriminant validity have been tested by calculating correlation coefficients with various scales that measure posttraumatic cognitions, as well as with PTSD symptoms, and symptoms of depression and anxiety. The Japanese version of the PMBS has not yet been published, but our research team plans to create a Japanese version through a back-translation process.

11. トラウマ関連の罪悪感（Trauma-Related Guilt Inventory; TRGI）：
トラウマ体験後に抱きやすい罪悪感を測定する尺度である[29]。32 項目から構成され、5 件法(1.全く当てはまらない、2.あまりあてはまらない、3.まあまああてはまる、4.だいたいあてはまる、5.非常にあてはまる)で回答を求める。全般的罪悪感、後知恵バイアス、正当化の欠如、悪いことをしたという考え、という 4 下位尺度から構成される。本研究では、認知処理療法における同化に関連する認知に最も関連が強いと考えられる全般的罪悪感と後知恵バイアスを測定する 11 項目のみを使用する。施行時間は 3–6 分程度である。トラウマ関連の罪悪感を測定する尺度は様々なものがあるが、その中でも最も用いられ、心理測定学的データが報告されている尺度が TRGI である[30]。近年の研究では、罪悪感が PTSD 症状に関与していることが検証されつつあり[31]、本研究においても、CPT の有効性を左右する治療メカニズムのひとつとして想定している。日本版は当研究チームが翻訳したものであるが、原著者との連絡が取れないために、バックトランスレーション手続きを経ていない。

11. Trauma-Related Guilt (Trauma-Related Guilt Inventory; TRGI):
The TRGI is a scale designed to measure the guilt that individuals are prone to experience after a traumatic event. It consists of 32 items, with responses given on a 5-point Likert scale (1: Not at all, 2: Slightly, 3: Moderately, 4: Mostly, 5: Extremely). The scale includes four subscales: general guilt, hindsight bias, lack of justification, and the thought of having done something wrong. In this study, only 11 items related to general guilt and hindsight bias, which are considered most closely related to assimilation-related cognitions in Cognitive Processing Therapy, will be used. The administration time is approximately 3–6 minutes. Among various scales used to measure trauma-related guilt, the TRGI is the most widely used and has the most reported psychometric data. Recent research has increasingly confirmed the involvement of guilt in PTSD symptoms, and in this study, it is also considered one of the treatment mechanisms influencing the effectiveness of CPT. The Japanese version was translated by our research team; however, it has not undergone a back-translation process due to the inability to contact the original author.

12. 感情調整（Emotion Regulation Questionnaire; ERQ）：
　本尺度は、感情調整の 2 側面である認知再評価と感情表出抑制の下位尺度を持つ測度であり、10 項目から構成される[32]。信頼性と妥当性は、日本や米国において幅広く検証されている[33]。7 件法により回答を求める（1.全くあてはまらない、2.ほとんどあてはまらない、3.あまりあてはまらない、4.どちらでもない、5.ややあてはまる、6.かなりあてはまる、7.非常にあてはまる）。得点の高さが、それぞれの傾向が強いことを示す。日本版の信頼性として内的整合性と 2 ヶ月間の間隔を置いた検査－再検査信頼性が検討されており、認知再評価では α = .77, r = .61（p < .01）、感情表出抑制は α = .78, r

= .65（$p < .01$）と報告されている。確認的因子分析により、因子的妥当性が確認され、Big Five、怒り、自尊感情、主観的幸福感等との相関関係から、構成概念妥当性が確認されている[33]。

12. Emotion Regulation (Emotion Regulation Questionnaire; ERQ):

This scale is used to measure two aspects of emotion regulation: cognitive reappraisal and expressive suppression. The ERQ consists of 10 items. The reliability and validity of the scale have been widely tested in both Japan and the United States. Participants respond using a 7-point Likert scale (1: Not at all true of me, 2: Slightly true of me, 3: Somewhat true of me, 4: Neither true nor untrue, 5: Moderately true of me, 6: Mostly true of me, 7: Very true of me). Higher scores indicate a stronger tendency towards each aspect of emotion regulation. For the Japanese version, both internal consistency and test-retest reliability over a two-month interval have been examined, with cognitive reappraisal showing $\alpha = .77$ and $r = .61$ ($p < .01$), and expressive suppression showing $\alpha = .78$ and $r = .65$ ($p < .01$). Confirmatory factor analysis has validated the factor structure, and construct validity has been confirmed through correlations with the Big Five personality traits, anger, self-esteem, and subjective well-being.

13. セッション評価（Session Rating Scale; SRS）—治療者・患者評価：

SRS は、治療同盟尺度を参考にした 4 項目からなる尺度である。絆、課題の合意、目標の合意、そして全体的なセッション評価を測定する[34]。全 4 項目の Visual Analogue Scale である。筆者らの知る限り、日本版の信頼性と妥当性の報告はない。原版の信頼性として Cronbach’s alpha = .88、6 回の実施間での検査－再検査信頼性 $r = .64$ が報告されている。また、構成概念妥当性として、Helping Alliance Questionnaire II との中程度の相関（$r = .48$）、第 2/3 セッションで測定された SRS と、治療終結時のアウトカムとの相関（$r = .20, p < .01$）が報告されている。

13. Session Rating (Session Rating Scale; SRS) – Therapist and Patient Assessment:

The SRS is a scale consisting of four items that was developed with reference to therapeutic alliance scales. It measures the bond between therapist and patient, agreement on tasks, agreement on goals, and an overall evaluation of the session. All four items are assessed using a Visual Analogue Scale. To the best of the authors' knowledge, there are no reports on the reliability and validity of the Japanese version. For the original version, reliability has been reported with a Cronbach’s alpha of .88 and a test-retest reliability of $r = .64$ over six administrations. Construct validity is supported by a moderate correlation with the Helping Alliance Questionnaire II ($r = .48$) and a correlation between SRS scores measured in the second or third session and outcomes at the end of treatment ($r = .20$, $p < .01$).

治療者評価

Tharapist Assessment

14. 宿題遵守（Homework Compliance ; HC）：

HC は、認知処理療法の治療の一環で、セッションごとに治療者が尋ねる。前回のセッションで出された宿題について、それぞれ何回・何分実施したか、それがどの程度役に立ったかをセラピストが患者に尋ね、評価してもらう[5]。

14. Homework Compliance (HC):

HC is evaluated as part of Cognitive Processing Therapy (CPT). After each session, the therapist asks the patient how many times and for how many minutes they completed the homework

assigned in the previous session, and how helpful they found it. The therapist then evaluates this information.

15. 有害事象：
所定の項目（口渇、便秘、排尿障害、視力調節障害、起立性低血圧、眠気、倦怠感、不眠、不安・焦燥、落ち込み・意欲低下、食欲不振、体重増加、体重減少、性欲低下、動悸、ふるえ、発汗、頭痛、ふらつき、その他）について、その有無を確認する。CPT 治療者または評価者は所定の用紙を用いて、「前回以来、身体的または精神的な症状で急に悪化したり発生しましたか」と口頭にて尋ね、患者の応答を求める（Solicit 形式であり、Voluntary 形式ではない）。

15. Adverse Events:
The presence or absence of specific adverse events (e.g., dry mouth, constipation, urinary retention, visual accommodation disorder, orthostatic hypotension, drowsiness, fatigue, insomnia, anxiety/agitation, depression/loss of motivation, loss of appetite, weight gain, weight loss, decreased libido, palpitations, tremors, sweating, headache, dizziness, and others) is confirmed. The CPT therapist or evaluator uses a designated form to ask the patient, "Since the last session, have you experienced any sudden worsening or new onset of physical or mental symptoms?" This inquiry is conducted in a solicited manner (i.e., the therapist actively asks) rather than relying on voluntary reporting by the patient.

第三者評価
Third-Party Evaluation

16. 治療遵守とコンピテンス（Therapist Adherence and Competence Protocol; TACP）：
TACP は、認知処理療法の治療遵守とコンピテンスを測定するために開発された尺度である。セラピストが特定の治療的行為を行ったかどうかという治療遵守を「はい」「いいえ」の 2 値で評価し、それらの行動をいかにうまく実施したかを 1–5（乏しい、素晴らしい）あるいは N/A で評価する。各セッション内容に応じて、セッションごとに評価する。また、すべてのセッションにおいて、“不可欠だが CPT 特有ではない要素（ラポールの構築、宿題のふり返り、セッションの構造化と効率的時間配分）”と“制限される要素（アジェンダからの逸脱、マニュアルや治療モデルにない介入の実施、15 分以上の逸脱）”のそれぞれ 3 項目ずつを評価する。必要に応じて、自由記述で追加のコメントを記入する。本試験では、各事例の最大セッション回数である 16 セッションと想定してその 5 分の 1 をランダムに評価する。ランダム化は、True Randomization process (www.random.org)により、研究実施前に評価者の一人が行う。評価は、治療スタッフではないセラピストが実施する。

16. Therapist Adherence and Competence (Therapist Adherence and Competence Protocol; TACP):
The TACP is a scale developed to measure therapist adherence to the Cognitive Processing Therapy (CPT) protocol and their competence in delivering the therapy. Therapist adherence is evaluated using a binary "yes" or "no" response to whether the therapist performed specific therapeutic actions. Competence is rated on a scale from 1 to 5 (poor to excellent) or marked as N/A, depending on how well these actions were executed. The evaluation is conducted for each session according to its content. Additionally, for all sessions, three elements categorized as "essential but not unique to CPT" (such as rapport building, homework review, and session structuring and efficient time management) and three elements categorized as "restricted

elements" (such as deviating from the agenda, performing interventions not in the manual or treatment model, and deviations longer than 15 minutes) are assessed. Additional comments can be provided in free text if necessary.
In this trial, one-fifth of the maximum 16 sessions for each case will be randomly selected for evaluation. The randomization is conducted prior to the study using the True Randomization process (www.random.org) by one of the evaluators. The evaluations are conducted by therapists who are not part of the treatment staff.

脳画像による評価
Brain Imaging Evaluation
16.脳画像データ（3D-T1 強調像、T2 強調像、FLAIR、ASL）
　3D-T1 強調像は、3 次元再構成が可能な連続切片を撮影する超高速撮像法である。T2 強調像および高解像度 T2 強調像は、大脳皮質など一般的な脳の体積を測定するための核磁気共鳴画像である。FLAIR（fluid attenuated inversion recovery）は、水からの信号を抑制した撮像法である。ASL（arterial spin labeling）は、磁気共鳴による脳血流動態を評価する撮像法である。DTI（diffusion tensor imaging；拡散テンソル像） は、水分子の拡散方向や大きさを規定する定数（テンソル）を求めることで神経繊維の走行などを評価できる撮像法である。

16. Brain Imaging Data (3D-T1-weighted images, T2-weighted images, FLAIR, ASL):
3D-T1-weighted imaging is an ultra-fast imaging technique that captures consecutive slices, allowing for three-dimensional reconstruction. T2-weighted images and high-resolution T2-weighted images are nuclear magnetic resonance images used to measure general brain volumes, including the cerebral cortex. FLAIR (fluid-attenuated inversion recovery) is an imaging technique that suppresses signals from fluids. ASL (arterial spin labeling) is an imaging method used to evaluate cerebral blood flow dynamics using magnetic resonance. DTI (diffusion tensor imaging) is an imaging technique that assesses the direction and magnitude of water molecule diffusion, allowing for the evaluation of nerve fiber pathways.

（11）症例登録、割付の方法
(11) Method of Case Registration and Allocation

Allocation
Sequence generation
　順番の作成はコンピュータにより行われる。すなわち、被験者は、慶応義塾大学医学部クリニカルリサーチセンターが開発した、中央登録方式の臨床研究割付登録システムであるコンピュータソフトウエア ARCS（Allocation and Registration Control System）、または NCNP 開発の EDC システムにより、最小化法を用いて１：１の割付比で介入群または対照群にランダムに割り付けられる。この際、トラウマの種類（単回性 vs. 持続性の 2 水準）を層別因子とする。設定を担当する慶應義塾大学担当者により、確率変数を組み込んだ設定とする。

Allocation
Sequence Generation
The sequence is generated by a computer. Specifically, participants are randomly assigned to either the intervention group or the control group with a 1:1 allocation ratio using the

minimization method. This is done through computer software known as ARCS (Allocation and Registration Control System), a central registration clinical research allocation system developed by the Clinical Research Center at Keio University School of Medicine, or through the EDC system developed by NCNP. The type of trauma (single event vs. continuous trauma) is used as a stratification factor. The settings, including probability variables, are configured by personnel at Keio University.

Allocation concealment mechanism

被験者の割付は、インフォームド・コンセントを経て評価面接により全ての包含・除外基準を確認した上で、オンライン上で行う。このように、研究者、評価者、被験者のいずれもランダム化のコードは割付されるまでの経過で知ることは不可能であるため、割付の隠蔽が保証される。

Allocation Concealment Mechanism

The allocation of participants is conducted online after confirming all inclusion and exclusion criteria through an evaluation interview following informed consent. In this way, the randomization code remains unknown to the researchers, evaluators, and participants until the allocation is completed, ensuring that allocation concealment is maintained.

Implementation

1) 順番の作成を含むソフトウエアの設定は、委託先が実施する。
2) 参加者に文書による同意を取得後、包含・除外基準を確認する必要な検査を行う。
3) 研究責任者・CPT担当者・コーディネーターのいずれかがラップトップパソコンを用い、システムにログインし、適格となった患者について割付に必要な項目をすべて入力する。面接室において研究対象者とともに入力内容を確認し、問題がなければ割付を実行する。実行されると、パソコン画面上にランダム化のコードおよび割付番号が表示される。割付群と割付番号は書き換え不能な形でシステム上に記録される。

Implementation

1) The configuration of the software, including sequence generation, is carried out by the contracted party.
2) After obtaining written consent from the participants, necessary tests are conducted to confirm the inclusion and exclusion criteria.
3) The principal investigator, CPT therapist, or coordinator logs into the system using a laptop computer, enters all required items for allocation concerning eligible patients, and confirms the input with the research participant in the interview room. If there are no issues, the allocation is executed. Once executed, the randomization code and allocation number are displayed on the computer screen. The allocation group and allocation number are recorded in the system in a manner that cannot be altered.

Plan to promote participant retention and complete follow-up

割付は参加者立会いのもとに、ラップトップパソコンを用いて行う。割付は研究責任者、コーディネーター、CPT担当者のいずれかが行い、もしも対照群に割付けられた場合には、その心情を共感的にサポートするとともに、通常治療の範疇においてどのような生活の工夫ができるかを話し合う。また、ベースライン・中間評価の結果をサマリーとしてまとめ、個々の参加者に応じたコメントを付して郵送する。この際に、次回の評価の日程を伝える用紙を同封する。

Plan to Promote Participant Retention and Complete Follow-Up

The allocation is conducted using a laptop computer in the presence of the participant. The allocation is performed by the principal investigator, coordinator, or CPT therapist. If the participant is assigned to the control group, the researcher provides empathetic support for their feelings and discusses possible lifestyle adjustments within the scope of Treatment-As-Usual. Additionally, the results of the baseline and mid-term evaluations are summarized and sent to each participant with personalized comments. A sheet with the schedule for the next evaluation is also enclosed in this mailing.

Blinding

Who will be blinded after assignment to interventions

独立評価者と効果安全性委員会委員が割付（割付群・評価時点）について盲険化され、参加者、CPT 治療者は割付の結果を知ることとなる。独立評価者は、スーパービジョンやコーディネートミーティング等、割付が判明しうる可能性のある委員会には一切参加せず、研究チームで共有するメーリングリストにも参加しない。独立評価者には ARCS のアカウントは付与されない。割付番号は割付された群に関わらず通し番号が用いられる。独立評価者に対する盲検化の解除は、最終的な分析データセットが固定されるまでは行わない。また、独立評価者のための治療知識用紙（IEKNO）を用いて、独立評価者は評価実施の際に推測した割付（割付条件と評価時点）を回答する（回答のための選択肢は、「介入群」、「対照群」、「わからない」）。試験終了後、その回答と実際の割付のクロス表を作成し、James's BI および Bang's BI による検討を行う[14]。

Blinding

Who Will Be Blinded After Assignment to Interventions

Independent evaluators and members of the Safety and Efficacy Committee will be blinded to the allocation (allocation group and evaluation time points), while participants and CPT therapists will be aware of the allocation results. Independent evaluators will not participate in any committees where the allocation might be revealed, such as supervision or coordination meetings, and they will not be included in the mailing list shared by the research team. Independent evaluators will not be granted ARCS accounts. The allocation numbers will be continuous regardless of the group to which they are assigned. Blinding for the independent evaluators will not be lifted until the final analysis dataset is fixed. Additionally, during evaluations, independent evaluators will use the treatment knowledge sheet (IEKNO) to record their guesses regarding the allocation (allocation condition and evaluation time point), with options for response being "intervention group," "control group," or "don't know." After the trial concludes, a cross-tabulation of these responses with the actual allocation will be created, and analyses using James's Blinding Index (BI) and Bang's BI will be conducted.

Circumstances under which unbinding is permissible　本研究では、独立評価者、効果安全性委員のみが盲険化されるため、盲険化の解除が必要になる事態は想定されない。

Circumstances Under Which Unblinding Is Permissible

In this study, only the independent evaluators and members of the Safety and Efficacy Committee are blinded, so situations requiring the unblinding of these individuals are not anticipated.

（12）統計解析方法

(12) Statistical Analysis Methods

Data management

研究で得られたデータは、Microsoft Accessにて作成されたデータベースに入力する。各入力フォームには、想定される範囲のデータ（例えば、5件法であれば1-5の範囲）のみしか入力できないよう設定する。データ入力は国立精神・神経医療研究センター認知行動療法センターにおいて、研究協力者および補助者が、研究対象者よりデータを得て可能な限り早く、即時性をもって入力する。Microsoft Accessへの入力はインターネットに接続されていないスタンドアローンのコンピュータを用いて行い、そのデータは強制暗号化機能を持つハードディスク・ドライブ（Network Attached Storage含む）に保存する。このハードディスクのパスワードは、研究責任者が許可した研究協力者および補助者のみが知ることができる。データが入力されたハードディスクおよび紙資料は国立精神・神経医療研究センター認知行動療法センターにおいて、鍵のかかる部屋に保管する。研究終了後5年間はデータを保存する。

Data Management

Data obtained from the study will be entered into a database created using Microsoft Access. Each input f orm will be configured to only allow data within the expected range (e.g., only values between 1 and 5 for a 5-point scale). Data entry will be conducted by research collaborators and assistants at the Center for Cog nitive Behavioral Therapy, National Center of Neurology and Psychiatry (NCNP), as promptly as possible aft er obtaining data from the research participants. The data will be entered into Microsoft Access on a standal one computer that is not connected to the internet. The data will be stored on a hard disk drive (including Network Attached Storage) with enforced encryption capabilities. The password for this hard disk will be kn own only to research collaborators and assistants authorized by the principal investigator. The hard disk cont aining the entered data, as well as any paper records, will be stored in a locked room at the Center for Co gnitive Behavioral Therapy, NCNP. The data will be preserved for five years after the completion of the stu dy.

Statistical methods

解析責任者（大庭真梨）がデータの固定と解析を行う。全ての主要・副次評価項目の解析において、介入群と対照群を比較する。全ての解析で、P値が小数点3までの値で表現され、0.001以下のものはp < .001と記載する。解析ソフトは、SPSS、SAS、Rを用いる予定である。統計的検定を用いる解析では、両側検定として有意水準を5%とする。本研究は主要評価項目の解析のためにデザインされているため、副次評価およびその他の解析（サブグループ解析・調整解析）は探索的なものとなる。

Statistical Methods

The data will be finalized and analyzed by the responsible statistician, Mari Oba. All primary and secondary outcome measures will be compared between the intervention group and the control group. In all analyses, p-values will be reported to three decimal places, with p-values of 0.001 or less reported as p < .001. The analysis will be conducted using SPSS, SAS, and R software. For statistical tests, a two-sided test with a si gnificance level of 5% will be applied. Since this study is designed primarily for the analysis of the primary outcome measures, analyses of secondary outcomes and other analyses (such as subgroup and adjusted anal yses) will be considered exploratory.

Analyses of primary and secondary outcomes

(1)主要評価項目の解析

・評価項目：17週のCAPS-5の得点

従属変数を pre, middle, postの評価項目とし、独立変数の固定効果要因を割付（介入群=0 vs対照群=1）と測定時点 (pre=0, middle=1, post=2)および両要因の交互作用項 (割付け*測定時点)とし、変量効

果要因を反復測定した被験者として、線形混合モデルにより検討する。

Analyses of Primary and Secondary Outcomes

(1) Analysis of the Primary Outcome

- **Outcome Measure:** CAPS-5 score at 17 weeks
  The dependent variable will be the CAPS-5 scores at pre-treatment, mid-treatment, and post-treatme nt time points. The fixed effects in the model will include the allocation (intervention group = 0 v s. control group = 1), the measurement time point (pre = 0, middle = 1, post = 2), and the intera ction term between allocation and measurement time point (allocation * measurement time point). T he subject under repeated measurement will be treated as a random effect factor, and the analysis will be conducted using a linear mixed model.

(2) 副次評価項目の解析

・評価項目：17週時点のPCL-5

主要評価項目と同様に、線形混合モデルによる検討する。

・治療反応割合

介入群と対照群における治療反応割合について、リスク比の95%信頼区間を求める。

・有害事象

有害事象の一覧を作成する。

(2) Analysis of Secondary Outcomes

- **Outcome Measure:** PCL-5 score at 17 weeks
  Similar to the primary outcome analysis, the analysis will be conducted using a linear mixed model.
- **Treatment Response Rate:**
  The treatment response rate between the intervention group and the control group will be analyzed by calculating the 95% confidence interval for the risk ratio.
- **Adverse Events:**
  A list of adverse events will be compiled.

Methods for any additional analyses

(1) 症例の内訳

最大の解析対象集団（FAS; Full Analysis Set）、試験実施計画書に適合した対象集団（PPS; Per Protocol SetまたはCompleter）、中止症例数を群の識別とともに表示する。

(2) 治療の状況

認知処理療法の遵守状況（TACP評価）について集計する。連続値（セッションの全般的コンピテンス）については平均値と標準偏差を示し、二値については割合を示す。

(3) データの要約

連続値として得られる全ての検査項目について、群ごと検査時点ごとに基礎統計量（最大値、中央値、最小値、25%点、75%点、平均値、標準偏差）を算出する。CAPS-5の得点を、群ごとにそれぞれの時点で算出する。各指標は基礎統計量を算出し、個々の被験者の経時推移を図（折れ線グラフ）に示し、経時推移の要約をBox-Whisker plotに示す。

カテゴリーデータとして得られる全ての検査項目について、治療法ごと検査時点ごとにカテゴリーの集計を行う。

(4) 背景因子の解析

ベースラインにおける性別、年齢（18–40歳vs 41–70歳）、トラウマの種類（単回性 vs. 持続性）について群ごとにカテゴリーの集計（頻度あるいは平均値）を行う。頻度データに関してはFisherの正確度検定(exact test), 平均値に関してはt検定を実施する。

(5) その他の主な評価項目(PHQ-9、EQ-5D-5L、SDS、PCL-5 plus)についての解析
主要評価項目と同様に、線形混合モデルによる検討する。
また、各指標の経時推移については各時点の得点をBox-Whisker plotに示す。

(6) 治療期間中に毎週実施する項目についての解析
主要評価項目と同様に、線形混合モデルによる検討する。
また、各指標の経時推移については各時点の得点をBox-Whisker plotに示す。

(7) サブグループ解析
本研究では、サブグループ解析は予定していない。

(8) 調整解析
調整解析として、主要評価項目と副次評価項目の線形混合モデルによる解析モデルに、PTSDの重症度を固定効果に加えたモデルの分析を実施し、トラウマの種類による解析結果の頑健性を検討する。

(9) 脳画像データの解析
解析では、主要評価項目および副次評価項目と脳画像の関連を、多変量解析を用いて求める。解析にはMatlabを用いる予定である。

Methods for Any Additional Analyses

(1) Breakdown of Cases

The maximum analysis population (FAS; Full Analysis Set), the population that conforms to the study protocol (PPS; Per Protocol Set or Completer), and the number of cases discontinued will be displayed along with their group identification.

(2) Treatment Status

The compliance with Cognitive Processing Therapy (CPT) will be aggregated using the TACP evaluatio n. For continuous variables (e.g., overall competence in sessions), the mean and standard deviation will be reported, while for binary variables, the proportions will be presented.

(3) Data Summary

For all test items measured as continuous variables, basic statistics (maximum, median, minimum, 25th percentile, 75th percentile, mean, standard deviation) will be calculated for each group at each time poi nt. The CAPS-5 scores will be calculated at each time point for each group. Basic statistics will be ca lculated for each indicator, and individual participants' time-course changes will be illustrated with line graphs, while the summary of time-course changes will be presented with Box-Whisker plots.
For all test items measured as categorical data, category totals will be calculated for each treatment me thod and at each time point.

(4) Analysis of Background Factors

Baseline characteristics such as gender, age (18–40 years vs. 41–70 years), and type of trauma (single event vs. continuous trauma) will be summarized by category (frequency or mean value) for each grou p. Fisher's exact test will be used for frequency data, and t-tests will be conducted for mean values.

(5) Analysis of Other Main Outcome Measures (PHQ-9, EQ-5D-5L, SDS, PCL-5 plus)

These will be analyzed using a linear mixed model, similar to the primary outcome measures. Addition ally, time-course changes in each indicator will be presented as Box-Whisker plots.

(6) Analysis of Weekly Measures Conducted During the Treatment Period

These will also be analyzed using a linear mixed model, similar to the primary outcome measures. Ti me-course changes in each indicator will be presented as Box-Whisker plots.

(7) Subgroup Analysis

Subgroup analyses are not planned for this study.

(8) Adjusted Analysis

As an adjusted analysis, the linear mixed model for primary and secondary outcome measures will be analyzed with PTSD severity added as a fixed effect to examine the robustness of the results based on the type of trauma.

(9) Analysis of Brain Imaging Data

The relationship between the primary and secondary outcome measures and brain imaging will be explo

red using multivariate analysis. Matlab will be used for this analysis.

Definition of analysis population relating to protocol non-adherence, handling missing data

(1) 有効性および安全性の解析の対象集団

有効性および安全性の主要な解析対象は、ランダム割付けをされた全ての被験者である最大の解析対象集団（FAS; Full Analysis Set）とする。副次的な解析対象集団として試験実施計画書に適合した対象集団（PPS; Per Protocol SetまたはCompleter）とする。試験実施計画書に適合した集団は、試験登録となったものの何らかの理由で中止となった全例とする。

(2) 症例の分類の定義

適格例: 適格基準のすべてを満たし、除外基準のいずれにも該当しない症例
中止症例: 中止基準により試験を中止した症例

(3) 中止症例、欠測値などのデータの取扱い

1) 中止症例のデータの取扱い

・FASについては、中止時期によらず中止時点までに測定された値を評価に用いる。LMMにより欠測値に対応できるものと考えられるが、必要に応じて多重代入法を用いる。

・PPSについては、何らかの理由で中止となったり、中止症例となった場合、解析に含めない。

2) 主要評価項目（CAPS-5）の欠測値の処理

基本的には、線形混合モデルによって解析することで欠損値に対処する。さらに、“best”または”worst”のそれぞれのケースシナリオを仮定した感度分析と、欠損値へのimputation modelを採用した分析を実施する。

3) CAPS-5以外の欠測値

主要評価項目の処理に準じる。

上記に定めていない症例やデータの取り扱いは、研究責任者と解析責任者で協議、決定する。

Definition of Analysis Population Relating to Protocol Non-Adherence, Handling Missing Data

(1) Analysis Population for Efficacy and Safety

The primary analysis population for efficacy and safety is the Full Analysis Set (FAS), which includes all participants who were randomly assigned. The secondary analysis population is the Per Protocol Set (PPS) or Completer, which includes participants who adhered to the study protocol. Those who were registered in the study but discontinued for any reason are considered to be in the PPS.

(2) Definition of Case Classification

Eligible Cases: Cases that meet all inclusion criteria and do not meet any exclusion criteria.
Discontinued Cases: Cases that were discontinued based on the discontinuation criteria.

(3) Handling of Discontinued Cases, Missing Data, and Other Data

1. **Handling Data of Discontinued Cases**

For FAS, values measured up to the point of discontinuation, regardless of the timing of discontinuation, are used in the evaluation. It is assumed that missing values can be addressed by a Linear Mixed Model (LMM); however, multiple imputation methods may be used if necessary.
For PPS, cases that were discontinued for any reason or became discontinued cases are not included in the analysis.

2. **Handling of Missing Values for Primary Outcome (CAPS-5)**

Missing values are primarily addressed by analyzing using a linear mixed model. Additionally, sensitivity analyses assuming both "best" and "worst" case scenarios and analyses adopting an i

mputation model for missing values will be conducted.

3. **Missing Values for Outcomes Other Than CAPS-5**
These will be handled in accordance with the primary outcome methods.
The handling of cases or data not specified above will be discussed and decided by the princi pal investigator and the statictician.

Data monitoring
Composition of data monitoring committee

本試験では、研究責任者自身が、コーディネーター及びデータ管理者と連携して、施設モニタリング及び中央モニタリングを行い、その結果を効果安全性委員に報告する。基本的には、データ入力を佐藤珠恵が行い、随時、圷京子がCRF又は原資料とデータベースに入力されたデータの照合（施設モニタリング）を行う。原資料からCRFへの記入は、ベースライン・中間・介入後評価に関しては独立評価者が、基礎情報やCPTセッション時に行う評価についてはCPT担当者が行う（一部、コーディネーターが補助する）。中央モニタリングは解析担当が行い、半年に一度（10月末日、3月末日）、研究責任者から効果安全性委員（大野裕、野村俊明）に報告書が送られる。中央モニタリングは全症例に対して行い、施設モニタリングは登録された最初の3例を対象に行い、その後、登録第4–29番目の症例に対して、予めランダム抽出しておいた3例を対象に行う。どの事例をランダム抽出したかは、CRF作成者およびデータ入力担当者には伝えないようにする。その後、モニタリングにより問題が認められれば、随時追加のモニタリングを行う。研究責任者及び効果安全性委員会の委員が必要性を指摘した場合には、モニタリング委員会を開催する。また、効果安全性委員は、データ管理者へとデータ公開を求めることができる。データ取得と入力、モニタリングの流れは以下の通りである。

1. 研究候補者に関係するFirst contact（紹介等）：コーディネーターが研究用IDを付与
2. 同意取得後、ベースライン評価：評価結果を独立評価者がCRFに記入
3. 独立評価者作成のCRF及び自記式尺度（原資料）を佐藤がデータベースに入力
4. CPT担当者が基礎情報に関するCRFを記入
5. プロセス指標等、介入群の評価結果を佐藤がデータベースに入力
6. 中間・介入後評価結果を独立評価者がCRFに入力
7. 独立評価者が記入したCRF及び自記式尺度（原資料）を佐藤がデータベースに入力

※　随時、圷がCRF及び自記式尺度と入力されたデータの照合（施設モニタリング）
※　中央モニタリングは竹林が行う
※　半年毎に、研究責任者が効果安全性委員に報告書を送付

Description of interim analyses
(1) 中間解析
本試験では、中間解析を予定していない。

Data Monitoring

Composition of Data Monitoring Committee

In this study, the principal investigator, in collaboration with the coordinator and data manager, will conduct site monitoring and central monitoring, and report the results to the Safety and Efficacy Committee. Data entry will primarily be handled by Tamae Sato, while Kyoko Akutsu will periodically perform reconciliation (site monitoring) between the CRF or source documents and the data entered into the database. The independent evaluators will complete the CRF from the source

documents for baseline, mid-term, and post-intervention evaluations, while the CPT therapist will fill out the CRF for basic information and evaluations conducted during CPT sessions (with some assistance from the coordinator). Central monitoring will be performed by the data statictician, and a report will be sent from the principal investigator to the Safety and Efficacy Committee members (Yutaka Ono and Toshiaki Nomura) twice a year (at the end of October and March). Central monitoring will cover all cases, while site monitoring will focus on the first three registered cases, followed by three randomly selected cases from the 4th to 29th registered cases. The randomly selected cases for monitoring will not be disclosed to the CRF creators or data entry personnel. If issues are identified during monitoring, additional monitoring will be conducted as necessary. A monitoring committee meeting will be held if the principal investigator or members of the Safety and Efficacy Committee deem it necessary. Additionally, the Safety and Efficacy Committee may request data disclosure from the data manager. The data collection, entry, and monitoring process is as follows:

1. First contact related to research candidates (e.g., referrals): The coordinator assigns a research ID.
2. Baseline evaluation after obtaining consent: The independent evaluator records the evaluation results in the CRF.
3. Sato enters the CRF created by the independent evaluator and self-report measures (source documents) into the database.
4. The CPT therapist fills out the CRF related to basic information.
5. Sato enters the evaluation results of process indicators and other measures for the intervention group into the database.
6. The independent evaluator records the mid-term and post-intervention evaluation results in the CRF.
7. Sato enters the CRF and self-report measures (source documents) completed by the independent evaluator into the database.

- Akutsu periodically reconciles the CRF and self-report measures with the entered data (site monitoring).
- Central monitoring is conducted by Takebayashi.
- The principal investigator sends a report to the Safety and Efficacy Committee every six months.

Description of Interim Analyses

(1) Interim Analysis

This study does not plan to conduct any interim analyses.

（13）試料・情報の授受

本研究では、NCNPの外にある機関と試料・情報を授受する予定はない。

(13) Transfer of Samples and Information

In this study, there are no plans to transfer samples or information to institutions outside of the NCNP.

6. 研究対象者の選定方針

6. Policy for Selection of Research Participants

Participants

以下の選択基準の全てを満たし、除外基準のいずれにも該当しない心的外傷後ストレス障害患者を本臨床試験に登録し、割り付けを行う手続きに進む。

Participants

Patients with Post-Traumatic Stress Disorder (PTSD) who meet all of the following inclusion criteria and do not meet any of the exclusion criteria will be registered for this clinical trial and proceed to the allocation process.

（1）選択基準

(a)DSM-5 精神疾患の診断・統計マニュアルによる心的外傷後ストレス障害の診断を満たす（CAPS-5 にて評価）
(b)ベースライン時の年齢が 18 歳以上 70 歳以下
(c)本研究の目的、内容を理解し、自由意思による研究参加の同意を文書で得られる

【設定根拠】
(a) 本研究の対象疾患であるため
(b) 同意能力および当該介入の対象範囲として最大の許容範囲と考えられるため
(c) 研究倫理上で必須要件であるため

(1) Inclusion Criteria

(a) Meets the diagnostic criteria for Post-Traumatic Stress Disorder (PTSD) according to the DSM-5, as evaluated by CAPS-5.
(b) Age between 18 and 70 years at the time of baseline assessment.
(c) Able to understand the purpose and content of the study and provide written consent to participate voluntarily.

[Rationale for Setting]

(a) Because this is the target disease of the study.
(b) Because this is considered the maximum allowable range for the ability to consent and the scope of the intervention.
(c) Because this is an essential requirement from an ethical standpoint in research.

（2）除外基準

(a)ベースライン時に物質使用障害（重度）が認められる者
(b)ベースライン時に躁病エピソードもしくは精神病性障害が認められる者
(c)ベースライン時に著しい希死念慮が認められる者
※a, b, c については、精神疾患簡易構造化面接法（The Mini-International Neuropsychiatric Interview; M.I.N.I.）にて評価する。
(d)ベースライン評価時点において CPT の実施が困難な程度の身体疾患、重度認知機能障害が認められる者
(e)ベースライン評価時点で他の構造化された精神療法を受けている者
(f)その他研究責任者が本研究の対象として不適当と判断した者

【設定根拠】

(a) – (d): 安全性の配慮及び実施可能性の低い対象者の除外、(e, f): 有効性評価

※訓練事例としての組入れ（臨床試験への登録はしないが研究と同様の介入を研究として実施する場合）：包含基準(a)を満たさない場合であっても、包含基準(c)を満たし、除外基準(a) – (d):に該当しない場合、主任研究者は臨床試験で実施される CPT と同一の形式での、訓練事例として CPT 提供を被験者に提案することがある。これは、臨床試験期間が長期にわたることにより、試験開始当初の CPT 実施担当者の異動等により、CPT 担当者の数が限られる事態が想定され、CPT 担当者を新たに育成することが研究として必要となる可能性があるためである。

(2) Exclusion Criteria

(a) Individuals who have a severe substance use disorder at baseline.
(b) Individuals who exhibit a manic episode or psychotic disorder at baseline.
(c) Individuals who exhibit significant suicidal ideation at baseline.
*Items (a), (b), and (c) will be assessed using the Mini-International Neuropsychiatric Interview (M.I.N.I.).
(d) Individuals who have physical diseases or severe cognitive impairments at baseline that would make it difficult to carry out CPT.
(e) Individuals who are receiving other structured psychotherapies at the time of baseline evaluation.
(f) Any other individuals deemed unsuitable for this study by the principal investigator.

[Rationale for Setting]

(a)–(d): To ensure safety and exclude participants for whom the intervention is not feasible.
(e) and (f): To ensure the validity of efficacy evaluation.

*Inclusion as a Training Case (Cases not registered in the clinical trial but receiving the same intervention as the study):*Even if inclusion criterion (a) is not met, the principal investigator may propose the provision of CPT in the same format as the clinical trial as a training case if the individual meets inclusion criterion (c) and does not meet exclusion criteria (a)–(d). This is because it is anticipated that the clinical trial may extend over a long period, and there may be a need to train new CPT providers due to the potential transfer of those responsible for delivering CPT at the beginning of the trial.

7. インフォームド・コンセント等を受ける手続等

7. Procedures for Obtaining Informed Consent and Other Related Processes

1）インフォームド・コンセントの方法とその説明事項

試験の実施に際し、研究責任者または分担者は倫理審査委員会で承認の得られた下記の 1–18 の事項を含む別紙 1 の同意説明文書を被験者に渡し、文書および口頭による十分な説明を行い、被験者の自由意思による試験への参加についての同意を文書で得る。対象者が 20 歳未満の場合には、保護者（代諾者）と本人の双方に対して理解を求め同意を得る。

CRF に同意取得年月日を記載する。

1. 研究の名称、研究実施について所属機関の長の許可を受けている旨
2. 研究機関の名称及び研究責任者の氏名
3. 研究の目的及び意義
4. 研究が実施又は継続されることに同意した場合であっても随時これを撤回できる旨、研究が実施又は継続されることに同意しないこと又は同意を撤回することによって研究対象者等が不利益な取扱いを受けない旨
5. 研究対象者として選定された理由（研究対象となる方）
6. 研究の方法及び期間、参加協力事項
7. 研究対象者に生じる負担並びに予測されるリスク及び利益
8. 個人情報等の取り扱い（匿名化の方法を含む）、情報の保管
9. 情報の廃棄の方法、研究終了後のデータ取扱の方針
10. 研究対象者等の求めに応じて、他の研究対象者等の個人情報等の保護及び当該研究の独創性の確保に支障がない範囲内で研究計画書及び研究の方法に関する資料を入手又は閲覧できる旨並びにその入手又は閲覧の方法、MRI で偶発所見が発見された場合の対応
11. 経済的負担及び謝礼の内容
12. 研究により生じる知的財産権の帰属
13. 健康被害に対する補償の有無及びその内容
14. 研究に関する情報公開の方法

15.　研究の資金源、利益相反
16.　研究対象者等及びその関係者からの相談等への対応、問い合わせ先

1) Method of Informed Consent and Explanation Details

Before conducting the trial, the principal investigator or a designated co-investigator will provide the participants with a consent explanation document (Appendix 1), which includes the following 1–18 items approved by the ethics review committee. The investigator will provide a thorough explanation both in writing and verbally and will obtain the participant's written consent to voluntarily participate in the trial. If the participant is under 20 years old, consent must be obtained from both the participant and their guardian (substitute decision-maker). The date of consent acquisition will be recorded in the CRF.

1. The title of the study and confirmation that permission has been obtained from the head of the affiliated institution to conduct the study.
2. The name of the research institution and the name of the principal investigator.
3. The purpose and significance of the research.
4. The right to withdraw consent at any time, even if consent to participate or continue in the study has been given, and assurance that refusing to participate or withdrawing consent will not result in any disadvantageous treatment for the participant.
5. The reason for selecting the individual as a research participant (those who qualify as research subjects).
6. The methods and duration of the research, and what participation entails.
7. The burdens, potential risks, and benefits to the research participant.
8. Handling of personal information, including methods of anonymization and information storage.
9. Methods of data disposal and the policy on handling data after the study concludes.
10. The participant's right to access or view the research protocol and materials related to the study methods, to the extent that it does not compromise the protection of other participants' personal information or the originality of the research, as well as the procedure for doing so; and the response if incidental findings are discovered during MRI.
11. Financial obligations and the details of any compensation.
12. Ownership of intellectual property rights resulting from the research.
13. The presence or absence of compensation for health-related injuries and the details thereof.
14. The method of public disclosure of information regarding the research.
15. The source of funding for the research and any conflicts of interest.
16. The response to inquiries or consultations from research participants or their associates, and contact information.

また、感染症対策の観点からオンラインでの実施を患者が希望する場合には、オンライン実施に関する追加の説明と同意の手続きを行い、下記の 1–9 に関しての同意を得る。

1 研究の名称、所属機関の長の承認
2 研究機関の名称及び研究責任者
3 新型コロナウイルス感染症等の感染症予防対策としてのオンラインセッション実施の目的
4 オンラインセッションの定義・手段・内容
5 オンラインセッション実施の要件
6 オンラインセッション実施の手順
7 オンラインセッション実施のデメリットおよびリスク、メリット
8 録音・録画について
9 オンライン実施に関する相談等への対応と問い合せ先

Additionally, if the patient wishes to participate online due to concerns about infectious disease prevention, the investigator will provide additional explanations and obtain consent related to online implementation. Consent will be obtained for the following 1–9 items:

1. The title of the study and approval from the head of the affiliated institution.
2. The name of the research institution and the name of the principal investigator.
3. The purpose of conducting online sessions as a measure to prevent infectious diseases, such as COVID-19.
4. The definition, means, and content of the online sessions.
5. The requirements for conducting online sessions.
6. The procedures for conducting online sessions.
7. The disadvantages, risks, and benefits of conducting online sessions.
8. Information about recording (audio or video) of the sessions.
9. The response to inquiries or consultations regarding online implementation and contact information.

8. 試料・情報、個人情報等の取扱い（匿名化する場合の方法、個人情報の安全管理方法など）

8. Handling of Samples, Information, and Personal Information (Methods of Anonymization, Personal Information Security Management, etc.)

(1) 匿名化の方法

評価票の結果等研究で得られた臨床データは、国立研究開発法人国立精神・神経医療研究センター認知行動療法センターにおいて、解析の前に、匿名化担当者が連結可能匿名化して個人情報

管理者の管理のもとで保管される。この際、個人を識別できる情報（氏名、生年月日など）は削除され、研究用 ID が付され、認知行動療法センター内の LAN やインターネットに接続されていないパソコンにて電子ファイル化し、パスワードをかけ、暗号化した上でハードディスクドライブに保存する。解析は、上記の方法で連結可能匿名化されたデータを用いて、大庭真梨が行う。なお、本研究で使用される評価票は氏名を含む被験者の個人情報は記載されず、研究用 ID が使用される。

(1) Method of Anonymization

Clinical data obtained from the study, such as evaluation results, will be anonymized before analysis at the Center for Cognitive Behavioral Therapy, National Center of Neurology and Psychiatry (NCNP). The person in charge of anonymization, will perform linkable anonymization, and the data will be stored under the management of the personal information manager. During this process, personally identifiable information (such as names and dates of birth) will be removed, and the data will be assigned a research ID. The data will be converted into electronic files on a computer that is not connected to the LAN or the internet at the Center for Cognitive Behavioral Therapy. The files will be password-protected, encrypted, and stored on a hard disk drive. The analysis will be conducted by Mari Oba using the linkably anonymized data prepared according to the above method. Additionally, the evaluation forms used in this study will not include any personal information, such as names, and only the research ID will be used.

(2) 試料等の保存の方法

個人情報管理者は、同意書など個人情報を含む研究必須文書等の紙媒体や電子媒体、個人情報と研究用 ID の対応表について、漏洩、盗難、紛失しないように以下の通り管理・保存する。

(2) Method of Storing Samples and Related Materials

The personal information manager will manage and store paper and electronic media containing personal information, such as consent forms and other essential research documents, as well as the correspondence table linking personal information with research IDs, in the following manner to prevent leakage, theft, or loss.

「物理的安全管理」：

物理的安全管理措置は、あらゆる紙資料や電子データ（同意書、対照表、録画と録音のデータ、MRI データ）の取り扱いを想定している。

1. 個人データを取り扱う区域の管理：個人データは国立精神・神経医療研究センター内において、患者から直接取得される（病院内での症状評価面接や自己記入式尺度への回答、認知行動療法セッションの録画録音、MRI 撮像）。この個人データは、国立精神・神経医療研究センター７号館３階の認知行動療法センター内の居室のキャビネットの中に保管する。個人データを保管し扱う区域は国立精神・神経医療研究センター７号館３階の認知行動療法センター内とする。認知行動療法センターへの入室はカードキーで管理されており、カードキーはセンターに雇

用されている者のみが保有する。カードの所持状況は、認知行動療法センター長が帳簿を作り管理する。居室の鍵は、NCNP 全体の鍵管理の方針に従い、使用外の時間は NCNP 防災センターにて保管される。オンライン実施には、セキュリティ対策（ウイルス対策ソフトの実施とソフトウエアのアップデート）が取られている端末を用いる。

2. 機器及び電子媒体等の盗難等の防止：盗難防止のために、上記①の管理に加えて、キャビネットもそれぞれ施錠可能なものを用いる。さらに、キャビネットの鍵は、パスワード認証が必要なキーボックスに保管する。キーボックスは、特殊な方法を用いなければ脱着できないような強度で壁に固定する。
3. 電子媒体等を持ち運ぶ場合の漏えい等の防止：電子媒体は管理区域内のみで利用する。
4. 個人データの削除及び機器、電子媒体等の廃棄：匿名化された検査データなどの紙資料は、すべてただちに個人を特定するような情報（固有名詞など）を削除したかたちで保管する。これらの検査データなどと、ただちに個人が特定できる情報を含む研究データ（同意書、対応表、録音録画データ）は研究終了後 5 年後に個人情報をわからなくして、復元不可能な形で廃棄する。

"Physical Security Management":

The physical security management measures are intended to handle all paper materials and electronic data (consent forms, correspondence tables, recorded and audio data, MRI data).

1. **Management of Areas Handling Personal Data:**
Personal data will be collected directly from patients within the National Center of Neurology and Psychiatry (NCNP) (during in-hospital symptom evaluation interviews, completion of self-report scales, recording of cognitive behavioral therapy sessions, and MRI imaging). This personal data will be stored in cabinets within the offices of the Cognitive Behavioral Therapy Center on the third floor of Building 7 at NCNP. The area where personal data is stored and handled will be within the Cognitive Behavioral Therapy Center on the third floor of Building 7 at NCNP. Access to the Cognitive Behavioral Therapy Center is controlled by card keys, which are issued only to those employed at the center. The status of card key possession is recorded and managed by the director of the Cognitive Behavioral Therapy Center. The keys to the offices are stored at the NCNP Disaster Prevention Center when not in use, in accordance with NCNP's overall key management policy. For online implementations, devices with security measures (such as antivirus software and software updates) will be used.
2. **Prevention of Theft of Equipment and Electronic Media:**
In addition to the management measures mentioned above, lockable cabinets will be used to further prevent theft. The keys to the cabinets will be stored in a key box that requires password authentication. The key box will be securely fixed to the wall in such a way that it cannot be removed without special tools.

3. **Prevention of Leakage When Transporting Electronic Media:**
   Electronic media will only be used within the controlled area.
4. **Deletion of Personal Data and Disposal of Equipment and Electronic Media:**
   Paper materials, such as anonymized test data, will be stored with all immediately identifiable information (such as proper names) removed. After the study concludes, research data containing immediately identifiable information (consent forms, correspondence tables, recorded and audio data) will be anonymized and then disposed of in an irretrievable manner five years after the end of the study.

「技術的安全管理」：
技術的安全管理は電子化されたデータ（録画録音のデータ、MRI データ）の安全管理措置を想定しており、下記のような措置をとる。

1. アクセス制御：研究代表者の伊藤正哉、コーディネート担当の片柳章子・佐藤珠恵・牧野みゆき、MRI 研究担当者の宮前光宏及び蟹江絢子のみが録画及び録音データが保管された HDD にアクセスできる。研究遂行上の必要に応じて、研究代表者が認める者に一時的にアクセスを許可することがある（データ入力やモニタリングなど）が、その場合にはアクセス制御を許可された者の監督下での扱いとする。電子データは強制暗号化とパスワード認証が求められる HDD を用いてデータを保管する。オンライン実施に用いる端末は、オンラインセッションを行う者および研究コーディネーターのみがアクセス可能なものとする。オンライン実施時の録音データは速やかに保存用 HDD に移行し、端末内に保存しない。
2. アクセス者の識別と認証：上記 1 に定めた者のみが HDD に保管されたデータにアクセスでき、認証パスワードを知ることができるようにする。オンライン実施に用いる端末およびネットワークにおいても、アクセス者の認証設定を行う。
3. 外部からの不正アクセス等の防止：録音録画データの再生や MRI データの解析においては、インターネットに接続されない端末もしくは機器を用いる。
4. 情報システムの使用に伴う漏えい等の防止：電子データは外部ネットワークにつながった状態の機器には接続しない。録画された記録の一部については、コンサルテーションや事例検討のために、Dropbox business や Fleekdrive 等のセキュアなクラウドシステム上でデータを共有することがある。共有された録音データは、毎回のコンサルテーションや事例検討後に、確実にデータ消去を行う。

"Technical Security Management":
Technical security management is intended for the secure handling of digitized data (recorded audio/video data, MRI data) and includes the following measures:

1. **Access Control:**
   Only the principal investigator, Masaya Ito, coordination staff Akiko Katayanagi, Tamae Sato, and Miyuki Makino, and MRI research staff Mitsuhiro Miyamae and Ayako Kanie

will have access to the HDD where the recorded audio and video data are stored. If necessary for the execution of the study, the principal investigator may temporarily grant access to other individuals (e.g., for data entry or monitoring), but such access will be under the supervision of those authorized for access control. Electronic data will be stored on an HDD that requires forced encryption and password authentication. The devices used for online implementation will only be accessible to those conducting the online sessions and the research coordinators. Audio recordings made during online sessions will be promptly transferred to the storage HDD and not kept on the device used.

2. **Identification and Authentication of Accessors:**
   Only those specified in point 1 above will be able to access the data stored on the HDD and know the authentication password. Accessor authentication settings will also be applied to the devices and networks used for online implementation.
3. **Prevention of Unauthorized External Access:**
   For the playback of recorded audio/video data and the analysis of MRI data, devices or equipment not connected to the internet will be used.
4. **Prevention of Leakage During Information System Use:**
   Electronic data will not be connected to devices linked to external networks. Some recorded sessions may be shared on secure cloud systems, such as Dropbox Business or Fleekdrive, for consultations or case reviews. Shared audio data will be securely deleted after each consultation or case review.

「人的安全管理」：

1. 雇用契約及び委託契約の締結時における守秘義務規定：当研究は『平成22年規程第４０号保有する個人情報の保護に関する規程』が適用されるNCNP職員もしくは研究生によって実施される。
2. 研究者等に対する教育・訓練の実施：手順書の徹底を図るよう訓練を実施するとともに、NCNPで実施される『平成22年規程第４０号保有する個人情報の保護に関する規程』第８条にて規定されている研修をはじめ、コンプライアンス研修、倫理研修に参加する。
3. オンライン実施に関わる研究スタッフは、用いるシステムに関する基本要件を理解するための学習を行うこととする。研究組織において、オンライン実施に関するe-Learningや講習会に関する情報を常に更新できるよう、情報収集と学習を継続す

"Human Security Management":

1. **Confidentiality Obligations at the Time of Employment and Commission Contracts:**
   This study is conducted by NCNP employees or research students and is subject to the "Regulations for the Protection of Personal Information Held" (Regulation No. 40 of 2010).

2. **Education and Training for Researchers:**
   Training will be provided to ensure adherence to procedures, and researchers will participate in training stipulated by Article 8 of the "Regulations for the Protection of Personal Information Held" (Regulation No. 40 of 2010) conducted by NCNP, as well as in compliance and ethics training.
3. **Training for Research Staff Involved in Online Implementation:**
   Research staff involved in online implementation must undergo training to understand the basic requirements of the systems used. The research organization will continue to collect information and ensure continuous learning to keep updated on e-learning and workshops related to online implementation.

。

(3) 個人情報等の開示
　被験者が希望する場合には、研究において被験者を識別できる個人情報の内容（同意書、録画・録音されたセッション記録）に関する資料も閲覧することができるものとする。研究実施計画書についても、他の研究参加者の個人情報の保護や当該研究の独創性の確保に支障のない範囲内で閲覧することができる。

(3) Disclosure of Personal Information

If participants wish, they may view materials related to the personal information that can identify them in the study (such as consent forms and recorded session records). The research protocol may also be viewed, provided that it does not compromise the protection of other participants' personal information or the originality of the research.

(4) 研究成果の公表における個人情報への配慮
　本研究の結果は被験者を特定できないようにした上で、学術専門誌などにて公表される。

(4) Consideration of Personal Information in the Publication of Research Results

The results of this study will be published in academic journals or other scholarly outlets in a manner that ensures the participants cannot be identified.

9. 研究参加のリスクと研究がもたらすベネフィット

9. Risks and Benefits of Participating in the Research

（1）研究対象者に生じる負担

認知処理療法のために 16 週間に渡って毎週約 50 分の時間がかかるため、参加患者には時間的損失と交通費に伴う経済的負担が生じうる。なお、MRI 撮像に関する費用は研究費から支出するため、被験者の負担は生じない。

(1) Burden on Research Participants

Participants may experience a time burden due to the need to dedicate approximately 50 minutes each week for 16 weeks to Cognitive Processing Therapy (CPT). This participation could also result in

economic burdens related to time loss and transportation costs. However, the costs associated with MRI imaging will be covered by the research budget, so there will be no financial burden on the participants for that aspect.

（2）研究対象者に生じ得る不利益（有害事象不具合等）
本研究参加患者におけるリスクは、一般の外来診療に伴うもの以上は特にないものと考えられる。現在のところ認知処理療法による参加患者への健康被害はほとんどないと思われる。
(2) Potential Disadvantages for Research Participants (Adverse Events, Malfunctions, etc.)
The risks for patients participating in this study are not expected to exceed those associated with general outpatient treatment. Currently, there appears to be minimal health risk to participants undergoing Cognitive Processing Therapy (CPT).

Invasiveness and Harms
1) 本研究における侵襲性についての捉え方
侵襲とは、研究目的で行われる、穿刺、切開、薬物投与、放射線照射、心的外傷に触れる質問等によって被験者に身体又は精神に傷害又は負担が生じることを指す[35, 36]。このうち、傷害又は負担が小さいものは「軽微な侵襲」とされている。本研究では、評価項目において心的外傷について尋ねる質問（CAPS-5, PCL-5 等）が含まれ、被験治療としてトラウマに焦点を当てた認知処理療法（CPT）が含まれる。心的外傷に触れる質問は、PTSD に対する臨床及び研究でゴールドスタンダードとされるものであること、本研究の対象候補として紹介される PTSD 患者は平常的に PTSD 症状に悩まされている状態であることから、これらの質問によって、“確定的に研究対象者の身体又は精神”に“平常時に被る範囲を超える恒常性の変化[36]”を身体又は精神に及ぼすとは考えにくい。また、これらのゴールドスタンダードの評価項目の使用によって、有害事象が生じたという研究知見も見当たらない。一方で、認知処理療法については有害事象の報告がわずかに見られるため、これらを考慮して侵襲性を判断する必要がある。しかし、そうした検討を踏まえても、下記(2)に述べるように、認知処理療法が“確定的”に“平常時に被る範囲を超える恒常性の変化”を身体又は精神に及ぼすとは考えにくい。そのため、本研究は、「軽微な侵襲」を伴う研究として捉えられる。

1) Understanding of Invasiveness in This Study
Invasiveness refers to any action performed for research purposes, such as punctures, incisions, drug administration, radiation exposure, or questions that touch on psychological trauma, which may cause physical or mental harm or burden to the participants【35, 36】. Among these, actions that cause minor harm or burden are considered "minimal invasiveness." In this study, the evaluation items include questions about psychological trauma (e.g., CAPS-5, PCL-5), and the therapeutic intervention includes Cognitive Processing Therapy (CPT) focused on trauma. Questions addressing psychological trauma are considered the gold standard in both clinical and

research contexts for PTSD. The PTSD patients who are potential candidates for this study are typically already struggling with PTSD symptoms regularly. Therefore, it is unlikely that these questions will "definitively cause changes in homeostasis that exceed the range typically experienced by the body or mind" in these participants. Additionally, no research findings suggest that the use of these gold-standard evaluation items has led to adverse events. On the other hand, there are a few reports of adverse events associated with Cognitive Processing Therapy, so these should be considered when assessing invasiveness. However, even with these considerations, as described in section (2) below, it is unlikely that CPT will "definitively cause changes in homeostasis that exceed the range typically experienced by the body or mind." Therefore, this study is considered to involve "minimal invasiveness."

また、脳画像データの測定に関して、MRI 装置では、強力な磁石の力とラジオで用いられるものと同様の電波を利用して、非侵襲的に人間の脳を画像化し、その形態や機能を測定することができるものであり、撮像に用いられる磁場や電波の強さは世界的な基準に基づき、通常臨床で用いられる、人体組織に対して悪影響のない範囲の磁場と電波を使用する。よって、この研究に参加することによって、健康被害等の危険が生じる可能性はきわめて低いと考えられ、また、研究終了後に時間が経過してから自覚症状が現れることも原則的にない。しかしながら、万が一に起こり得る危険としては、一般の MRI 検査における安全性と同一である。つまり、MRI 室内に磁性体を持ち込むことにより、その持ち込まれた物体が強い磁気によって急激に動き、患者の身体に危害を与える可能性、誤って持ち込んだもの（時計、金属、キャッシュカードなど）が破損する危険性、身体に施された入れ墨や化粧品に含まれる磁性体が熱を帯びやけどをする可能性、あるいは体内の磁性体（残存している弾丸、旧式の整形外科的固定具や脳動脈瘤クリップなど）が強い磁気によって動き、身体を傷つける可能性等がある。また、MRI による強い磁気やパルス波の妊娠中または妊娠の可能性のある女性に対する影響については安全の確証がないため、そのような可能性のある女性に対しては施行することができない。撮像において考えられる不快な状態は、機器の中に動かずに仰臥位になっている、ということそのものによる疲労感や、MRI 撮像に伴う頭皮への神経刺激、閉所に入ることによって引き起こされる圧迫感などがあるが、いずれもチェックリストの確認と、注意深い事前確認により防止が可能なものである。

Additionally, regarding the measurement of brain imaging data, the MRI device uses powerful magnets and radio waves similar to those used in radios to non-invasively image the human brain and measure its structure and function. The strength of the magnetic field and radio waves used in imaging is based on global standards, employing levels that are commonly used in clinical settings and are harmless to human tissues. Therefore, the likelihood of health risks or other dangers arising from participation in this study is considered extremely low, and it is generally unlikely that any symptoms would appear after the study has concluded. However, potential risks, although very rare,

are the same as those associated with standard MRI examinations. These risks include the possibility that bringing magnetic objects into the MRI room could cause them to move rapidly due to the strong magnetic field, potentially causing harm to the patient. There is also a risk of damage to items accidentally brought into the MRI room (such as watches, metal objects, or credit cards). Additionally, magnetic materials in tattoos or cosmetics might heat up and cause burns, or magnetic materials within the body (such as residual bullets, old orthopedic implants, or brain aneurysm clips) might move and cause injury due to the strong magnetic field. Moreover, the effects of strong magnetic fields and pulse waves generated by MRI on pregnant women or those who might be pregnant are not guaranteed to be safe, so MRI cannot be performed on women with such potential conditions. Potential discomforts during imaging include fatigue from lying still in a supine position inside the machine, nerve stimulation to the scalp during MRI imaging, and a feeling of claustrophobia from being in a confined space. However, all these discomforts can be prevented through careful checklist verification and prior careful examination.

2) 有害事象発生時の被験者への対応

上述の認識に基づき、本研究では患者の Visit ごとに、有害事象全体の評価の他に、自殺念慮のリスクに特化して SIDAS により評価する。有害事象が発現した場合、CPT 担当者はその内容と重篤性を評価する。CPT 担当者は出来る範囲で必要な対応を施し、研究責任者と相談の上で必要と判断された場合には、NCNP での担当医（もしくは主治医）または外部医療機関での主治医と相談し、必要な処置を施すよう求め、（CPT を中止した場合も含めて）経過を充分観察することとする。CPT 担当者は有害事象の内容、発現日・消失日、程度、処置、転帰、重篤性評価、治療との関連性等をカルテおよび CRF に記載する。また、有害事象に対する治療が必要となった場合には、担当医または主治医と相談の上、CPT 担当者、担当医、または主治医が被験者にその旨を伝える。他の緊急な対応を要する事象の発生を含め、有害事象発生時には Figure 3 に従って対応を行う。

また、脳画像データの測定に関して、MRI 撮像中に気分が悪くなったり、なんらかの不都合が生じたりした際には、いつでも撮像を中止することが可能である旨を事前に患者に伝える。そして、上記のような訴えが患者からあった場合は、患者と相談の上、必要に応じて撮像を中止し、Figure 3 に従って対応を行う（この場合、Figure 内の「CPT 担当者」は「MRI 実施者」となる）。

2) Response to Participants in the Event of Adverse Events

Based on the above understanding, in this study, the overall assessment of adverse events will be conducted at each patient visit, with a specific focus on evaluating the risk of suicidal ideation using the SIDAS. If an adverse event occurs, the CPT therapist will assess its nature and severity. The therapist will provide necessary interventions to the best of their ability and, if deemed necessary after consulting with the principal investigator, will consult with the attending physician at NCNP (or the primary care physician) or an external medical institution's primary care physician to ensure

the necessary measures are taken. The patient's progress will be carefully monitored, even if CPT is discontinued. The CPT therapist will document the details of the adverse event, including the onset and resolution dates, severity, treatment, outcome, severity assessment, and its relation to the therapy in the medical record and CRF. If treatment for the adverse event is necessary, the CPT therapist, in consultation with the attending or primary care physician, will inform the participant. Any response to adverse events, including the occurrence of other emergencies, will follow the procedures outlined in Figure 3.

Additionally, regarding the measurement of brain imaging data, patients will be informed beforehand that they can stop the MRI scan at any time if they feel unwell or encounter any issues during the imaging process. If such complaints are made by the patient, the imaging will be stopped as necessary after consulting with the patient, and the response will follow the procedures outlined in Figure 3 (in this case, "CPT therapist" in the figure would refer to the "MRI operator").

オンライン実施時に有害事象が発生した場合も、上述と同様の対応を行う。オンライン実施時に緊急対応が必要な事象が発生した場合には、事前に計画しておいた緊急連絡先への連絡、主治医もしくは救急医療機関への受診に関して研究スタッフと研究参加者が相談し、最も適切と考えられる対応を行い、経過を充分観察することとする。

In the event of an adverse event occurring during online implementation, the same response as described above will be taken. If an emergency requiring immediate response occurs during online implementation, the research staff and the participant will consult and contact the pre-planned emergency contact, and arrange for the participant to see their primary care physician or visit an emergency medical facility. The most appropriate action will be taken, and the participant's progress will be carefully monitored.

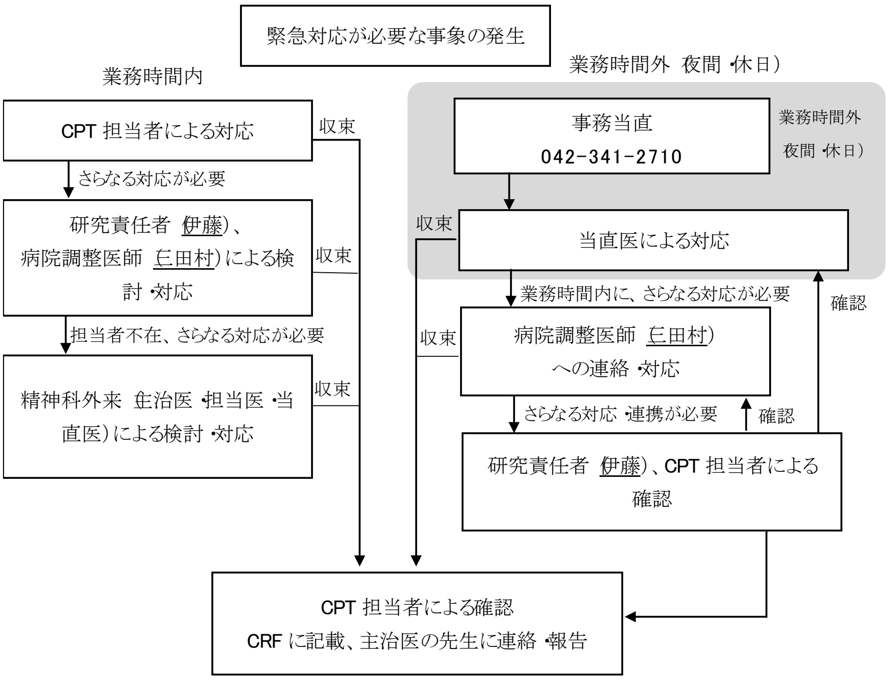

Figure 3. 有害事象発生時の対応

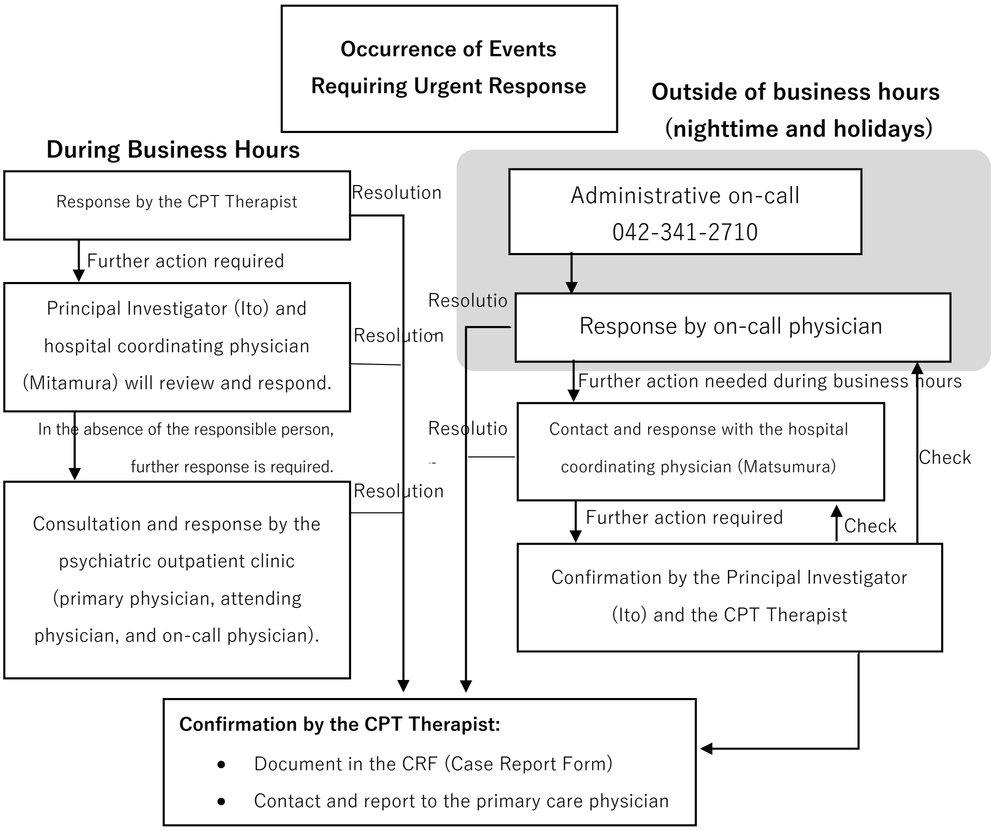

Figure 3. Response to Adverse Events

3) 有害事象の重篤性の評価

有害事象の用語には、Common Terminology Criteria for Adverse Events (CTCAE) Version 4.0[37]、MedDRA/J（Medical Dictionary for Regulatory Activities/J:ICH 国際医薬用語集日本語版）に対応している有害事象共通用語規準 v4.0 日本語版 JCOG 版[38]を用い、有害事象の詳細は CRF の備考欄に記入する。

程度については、JCOG に従い、以下のように定義する。

Grade 1 軽症；症状がない、または軽度の症状がある；臨床所見または検査所見のみ；治療を要さない

Grade 2 中等症；最小限／局所的／非侵襲的治療を要する；年齢相応の身の回り以外の日常生活動作の制限

Grade 3 重症または医学的に重大であるが、ただちに生命を脅かすものではない；入院または入院期間の延長を要する；活動不能／動作不能；身の回りの日常生活動作の制限

Grade 4 生命を脅かす；緊急処置を要する

Grade 5 有害事象による死亡

3) Assessment of Adverse Event Severity

The terminology for adverse events will follow the Common Terminology Criteria for Adverse Events (CTCAE) Version 4.0【37】and the Japanese version of the MedDRA (Medical Dictionary for Regulatory Activities): MedDRA/J (ICH International Medical Terminology Japanese Edition) as per the JCOG (Japan Clinical Oncology Group) version of the Common Terminology Criteria for Adverse Events v4.0【38】. The details of adverse events will be recorded in the remarks column of the CRF.

The severity levels will be defined according to JCOG as follows:

Grade 1 (Mild): No symptoms or mild symptoms; only clinical or diagnostic observations; no treatment required.

Grade 2 (Moderate): Minimal, local, or non-invasive treatment required; limitation of age-appropriate instrumental activities of daily living.

Grade 3 (Severe or medically significant but not immediately life-threatening): Requires hospitalization or prolongation of hospitalization; disabling; limitation of self-care activities of daily living.

Grade 4 (Life-threatening): Requires urgent intervention.

Grade 5 (Death related to adverse event): Death due to an adverse event.

4) その他の有害事象とその報告
その他の有害事象については、「12. Outcomes (2). 安全性評価」に記載した項目（口渇、便秘、排尿障害、視力調節障害、起立性低血圧、眠気、倦怠感、不眠、不安・焦燥、落ち込み・意欲低下、食欲不振、体重増加、体重減少、性欲低下、動悸、ふるえ、発汗、頭痛、ふらつき、その他）を確認し、該当するものがあれば CRF に記載する。重篤でない有害事象については、前年度の発生状況について、次年度の 6 月までに倫理委員会に報告する。

4) Other Adverse Events and Their Reporting

For other adverse events, the items listed in "12. Outcomes (2). Safety Evaluation" (such as dry mouth, constipation, urinary retention, visual accommodation disorder, orthostatic hypotension, drowsiness, fatigue, insomnia, anxiety/agitation, depression/loss of motivation, loss of appetite, weight gain, weight loss, decreased libido, palpitations, tremors, sweating, headache, dizziness, and others) will be checked. If any of these are applicable, they will be recorded in the CRF. Non-serious adverse events from the previous year will be reported to the ethics committee by June of the following year.

＊オンラインでの実施に関する不利益・リスクとその対策

・オンライン実施に急病急変の事態が生じた場合には、来院時に予約外での診察を行うような対面での即時的な対応が実施できない。万が一、対応が必要な場合には、予め同意していた安全計画に基づき、医療機関を受診し、その後の経過について報告を求める。

・オンライン実施にかかる通信料などの費用負担が生じる。

・端末機器などの設定の労力や費用負担が生じる。設定の手続きに関しては研究スタッフがオンライン実施に関するＩＣ取得時などに支援する。

・通信状況等、技術的な障害によってスムーズにやりとりができない場合がある。通信が途切れた場合には、予め用意しておいた連絡先に電話をして、問題解決を行う。

・対面に比べて、より積極的な協力が必要となる場合がある。

・家庭でのプライバシー環境の整備が必要となる（病院で実施するのと同じように、静かでプライバシーの守られる、物理的に遮断された空間を準備する必要がある）。オンライン実施に関するＩＣ時に、適切な環境が確保できるかを確認し、それが困難な場合には、どのようにオンライン実施が可能となるかを研究スタッフと研究参加者とで相談する。

・情報漏洩や不正アクセス等のセキュリティリスクを完全に排除できない。対策は、研究チームとしても厚生労働省のガイドラインや、国立精神・神経医療研究センターの情報セキュリティ規程に則り、細心の注意を払って実施する。

Disadvantages/Risks Related to Online Implementation and Their Countermeasures

- In the event of a sudden illness or emergency during online implementation, immediate in-person responses such as unscheduled visits to the clinic cannot be provided. If such a situation requires attention, the participant will be asked to visit a medical institution based on a pre-agreed safety

plan and report on their progress afterward.

- There may be costs associated with communication fees for online implementation.
- Participants may incur the labor and costs associated with setting up devices. Research staff will assist with the setup process during the informed consent (IC) process related to online implemen tation.
- There may be instances where smooth communication is disrupted due to technical issues such as poor internet connection. In the event of a communication breakdown, participants should contact the pre-arranged emergency contact to resolve the issue.
- More proactive cooperation from the participant may be required compared to in-person sessions.
- It is necessary to establish a private environment at home (similar to the privacy ensured in a ho spital setting, a quiet, private, and physically isolated space should be prepared). During the IC p rocess for online implementation, research staff will confirm whether a suitable environment can be secured. If this is difficult, research staff and participants will discuss how online implementati on can be made possible.
- Security risks, such as information leaks and unauthorized access, cannot be completely eliminate d. The research team will take measures in strict accordance with the guidelines of the Ministry of Health, Labour and Welfare and the information security regulations of the National Center of Neurology and Psychiatry, taking utmost care in implementation.

(3) リスクを最小化する方法

参加患者の安全性を確保するための事項

参加患者の安全性を確保するため、スクリーニング時の状態に関する情報を十分に収集する。一方、治療実施期間においては、参加患者の自覚症状、他覚所見、その他臨床情報等の確認を行うこととする。また、治療実施中に担当治療者が切迫した自殺念慮など参加患者の安全性を確保するのに重大な情報を得た場合、その旨本人の了解が得られなくても主治医に報告することがあることを、あらかじめ参加患者から同意を得るものとする。また、脳画像データの測定に関しては、「ヒト脳画像の非侵襲的研究」の倫理問題等に関する指針（日本神経科学会「ヒト脳機能の非侵襲的研究」に関する倫理小委員会）に従うものとする。

(3) Methods to Minimize Risks

Ensuring the Safety of Participating Patients

To ensure the safety of participating patients, comprehensive information will be collected about the patient's condition during the screening process. During the treatment period, the patient's subjective symptoms, objective findings, and other clinical information will be regularly confirmed.
Additionally, if the therapist obtains critical information during treatment, such as imminent suicidal ideation, that is crucial for ensuring the patient's safety, it will be reported to the primary care physician even without the patient's consent. Prior consent for this reporting will be obtained from

the participant. For the measurement of brain imaging data, the study will adhere to the guidelines on ethical issues related to "Non-invasive Research on Human Brain Imaging" (Subcommittee on Ethics of "Non-invasive Research on Human Brain Function" of the Japan Neuroscience Society).

（4）予想されるベネフィットと（1）（2）（3）を踏まえた総合評価

わが国において PTSD に対する認知処理療法実施者が限られている現状のなか、その療法を受けられるというメリットがある。研究期間中は、種々の病状評価アンケートや評価面接が行われるため、精緻な症状評価が受けられるというメリットもある。

以上を踏まえた総合評価として、負担やリスク、それに対する対策に比したベネフィットが極めて小さいとは言えず、患者自身の同意が得られた場合には、研究への参加が許容されうるものと判断する。

＊オンラインでの実施に関する利益

・来院のための交通経路、来院中の感染（閉鎖空間での 1 時間程度のセッションの実施）など、病院に来院して対面で研究参加をする場合に比べて、感染への可能性を大幅に下げることができると考えられる

・交通費など、来院にかかる時間や費用が削減される

(4) Anticipated Benefits and Overall Evaluation Based on (1), (2), and (3)

Given the current situation in Japan, where there are limited practitioners of Cognitive Processing Therapy (CPT) for PTSD, one significant benefit is the opportunity to receive this therapy. During the research period, participants will undergo various symptom assessment questionnaires and evaluation interviews, providing the benefit of receiving a detailed symptom evaluation.

Considering the overall evaluation based on the above, the benefits are not considered negligible when compared to the burdens and risks, along with the countermeasures in place. Therefore, if the patient gives informed consent, participation in the study is deemed permissible.

Benefits of Online Implementation

Compared to participating in the study face-to-face at the hospital, online implementation is expected to significantly reduce the risk of infection, as it avoids the need for transportation to the hospital and conducting sessions in a closed space for about an hour.

It also reduces the time and costs associated with transportation, such as travel expenses.

（5）個々の研究対象者における中止基準

(5) Discontinuation Criteria for Individual Research Participants

Criteria for discontinuing or modifying allocated interventions

(1) 介入中止基準

個々の症例が以下のいずれかの中止基準に該当する場合、その症例の試験治療を中止する。中止の日付・時期(治療期間・追跡調査期間)、中止の理由、経過をカルテならびに CRF に明記するとともに、中止時点で主要評価項目と副次評価項目に関する評価を行い有効性・安全性の評価を行う。有害事象発生により中止した場合は、研究責任者は担当治療者、スーパーバイザー、主治医、NCNP 担当医と状況を検討し、介入中止になっ

た理由に応じて適切な対応を決定し、可能なかぎり原状に回復するまでフォローする。中止後も可能なかぎり 17 週の時点での評価を行う。中止基準に従って CPT を中止した場合、治療後時点（17 週）に予定されている検査を可能な限り行う。インフォームド・コンセントの際にも、介入が中止した場合も 17 週の評価は可能な限りの協力を依頼することを被験者に確認する。

1) 被験者から試験参加の辞退の申し出や同意の撤回があった場合
2) 被験者との連絡が不通となり、1 ヶ月以上経過した場合
3) 重篤な有害事象により試験治療の継続が困難な場合
4) 試験全体が中止された場合
5) その他の理由により、研究責任者、担当治療者、スーパーバイザーが試験を中止することが適当と判断した場合

【設定根拠】
1): GCP に従う、3): 安全性確保のため、2)、4)、5) : 倫理的観点から

(2) 後観察中止基準
上記中止基準に準ずる。

(1) Criteria for Discontinuing Interventions

If an individual case meets any of the following criteria for discontinuation, the trial intervention for that case will be stopped. The date and time of discontinuation (during the treatment period or follow-up period), reasons for discontinuation, and subsequent progress will be clearly documented in the medical record and CRF. An evaluation related to the primary and secondary outcome measures will be conducted at the time of discontinuation to assess efficacy and safety. If the discontinuation is due to the occurrence of an adverse event, the principal investigator will discuss the situation with the responsible therapist, supervisor, primary care physician, and the NCNP physician, decide on appropriate actions based on the reason for discontinuation, and follow up until the condition is restored as much as possible. Even after discontinuation, an evaluation at the 17-week point will be conducted as much as possible. If CPT is discontinued according to the discontinuation criteria, the assessments scheduled for the post-treatment period (17 weeks) will be performed as much as possible. During the informed consent process, participants will be informed that, even if the intervention is discontinued, their cooperation in the 17-week evaluation will be requested as much as possible.

1. If the participant requests to withdraw from the study or revokes consent.
2. If contact with the participant is lost for more than one month.
3. If severe adverse events make it difficult to continue the trial treatment.
4. If the entire trial is discontinued.
5. If, for any other reason, the principal investigator, responsible therapist, or supervisor deems it appropriate to discontinue the trial.

[Rationale for Setting] 1): In accordance with GCP; 3): For safety assurance; 2), 4), 5): For ethical reasons.

(2) Criteria for Discontinuing Follow-Up

The criteria for discontinuing follow-up are the same as those mentioned above.

（6）研究全体の中止基準

試験の中止、中断

倫理委員会により中止の勧告あるいは指示があった場合は、試験を中止する。研究責任者および効果安全性評価委員で構成する委員会は、以下の事項に該当する場合は試験実施継続の可否を検討する。

1)　治療の品質、安全性、有効性に関する重大な情報が得られたとき。
2)　倫理委員会により実施計画等の変更の指示があり、これを受入れることが困難と判断されたとき。

試験の中止または中断を決定した時は、速やかに倫理委員会にその理由とともに文書で報告する。試験中止の決定を行った後、関係医師および心理士等に速やかに伝達し、中止後の処理にあたるものとする。

試験の終了

試験の終了時には、研究責任者は、速やかに試験終了報告書を倫理委員会に提出する。

(6) Criteria for Discontinuing the Entire Study

Discontinuation or Suspension of the Trial

The trial will be discontinued if the ethics committee recommends or instructs its termination. The committee, composed of the principal investigator and the Safety and Efficacy Evaluation Committee, will review the continuation of the trial if any of the following situations occur:

1. Significant information concerning the quality, safety, or efficacy of the treatment is obtained.
2. The ethics committee instructs changes to the implementation plan, and it is determined that accepting these changes would be difficult.

If a decision to discontinue or suspend the trial is made, the reason for this decision will be promptly reported to the ethics committee in writing. After the decision to discontinue the trial, the relevant physicians, psychologists, and other involved personnel will be promptly informed, and appropriate measures will be taken following the discontinuation.

Completion of the Trial

Upon completion of the trial, the principal investigator will promptly submit a trial completion report to the ethics committee.

10．研究に用いられる情報に係る資料の保管及び廃棄の方法

本研究によっていかなる結果が得られても、その結果は被験者を特定できないようにした上で、学術専門誌などにて公表される。Primary Outcome Paper は主任研究者（伊藤）が草稿を執筆し、共著者の確認・修正を全著者が納得するまで繰り返す。最終稿を、次項の authorship の配慮を踏まえた上で、研究終了後可能な限り迅速に公表する。二次解析などの他の論文は、Primary Outcome Paper が公表された後に公表することとする。

10. Methods for Storing and Disposing of Materials Related to Information Used in the Research

Regardless of the results obtained from this study, they will be published in academic journals in a manner that ensures participants cannot be identified. The primary outcome paper will be drafted by the principal investigator (Ito), and the draft will be repeatedly reviewed and revised by co-authors until all authors are satisfied. The final version will be published as quickly as possible after the study is completed, taking into account the considerations related to authorship mentioned in the next section. Other papers based on secondary analyses will be published after the primary outcome paper has been made public.

11．研究機関の長への報告内容及び方法

研究機関の長（理事長）への報告については下記の通りとする。

（1）年 1 回、研究実施状況について倫理委員会を通して報告する。
（2）重篤な有害事象が発生した場合は、速やかに倫理委員会を通して報告する。
（3）本研究に関係する有効性・安全性に関する重要な情報が得られた場合は、研究責任者の見解を記載し、理事長に報告し、研究継続の適否について倫理委員会の審査を受ける。
（4）研究の終了時（中止または中断の場合を含む）には、理事長に報告する。

11. Content and Method of Reporting to the Head of the Research Institution

Reports to the head of the research institution (President) will be made as follows:

1. An annual report on the status of the research implementation will be submitted through the ethics committee.
2. If a serious adverse event occurs, it will be promptly reported through the ethics committee.
3. If important information related to the efficacy or safety of the research is obtained, the principal investigator's opinion will be included in the report to the President, and the ethics committee will review the appropriateness of continuing the research.
4. Upon completion of the research (including in cases of discontinuation or suspension), a report will be submitted to the President.

12. 研究に係る資金と利益相反に関する状況

本研究は、日本学術振興会科学研究費補助金　基盤研究（A）「心的外傷後ストレス障害に対する認知処理療法の有効性及び臨床展開（研究代表者：堀越勝・分担研究者：伊藤正哉、研究課題番号：15H01979）」、基盤研究（B）「トラウマ関連障害への認知処理療法の均てん化のための包括研究（研究代表者：堀越勝・分担研究者：伊藤正哉、研究課題番号：19H01767）」、基盤研究（B）「トラウマ関連障害への認知処理療法の有効性及び作用機序の検証と適用拡大（研究代表者：堀越勝・分担研究者：伊藤正哉、研究課題番号：22H01097）」、および、独立行政法人日本医療研究開発機構　障害者対策総合研究開発事業「新たな認知行動療法プログラムの開発と普及に関する研究（研究分担者：伊藤正哉、課題番号：16769055）」、の助成を受けて実施される。本研究のデザイン、管理、分析、報告はこれらの研究費提供機関とは完全に独立してなされる。

12. Funding and Conflict of Interest Related to the Research

This study is funded by the following grants:

- Japan Society for the Promotion of Science (JSPS) Grant-in-Aid for Scientific Research (A) "Efficacy and Clinical Deployment of Cognitive Processing Therapy for Post-Traumatic Stress Disorder" (Principal Investigator: Katsumi Horikoshi; Co-Investigator: Masaya Ito; Project Number: 15H01979).
- JSPS Grant-in-Aid for Scientific Research (B) "Comprehensive Research for the Dissemination of Cognitive Processing Therapy for Trauma-Related Disorders" (Principal Investigator: Katsumi Horikoshi; Co-Investigator: Masaya Ito; Project Number: 19H01767).
- JSPS Grant-in-Aid for Scientific Research (B) "Verification of Efficacy and Mechanisms of Action of Cognitive Processing Therapy for Trauma-Related Disorders and Expansion of Its Application" (Principal Investigator: Katsumi Horikoshi; Co-Investigator: Masaya Ito; Project Number: 22H01097).
- Japan Agency for Medical Research and Development (AMED) Comprehensive Research on Disability Measures "Research on the Development and Dissemination of a New Cognitive Behavioral Therapy Program" (Co-Investigator: Masaya Ito; Project Number: 16769055).

The design, management, analysis, and reporting of this research will be conducted entirely independently of these funding agencies.

13. 研究に関する情報公開の方法

Dissemination policy

Plans to communicate trial results to participants, healthcare professionals, the public groups

(1) 公共的・学術的な場での結果の公表

本研究によっていかなる結果が得られても、その結果は被験者を特定できないようにした上で、学術専門誌などにて公表される。Primary Outcome Paper は主任研究者（伊藤）が草稿を執筆し、共著者の確認・修正を全著者が納得するまで繰り返す。最終稿を、次項の authorship の配慮を踏まえた上で、研究終了後可能な限り迅速に公表する。二次解析などの他の論文は、Primary Outcome Paper が公表された後に公表することとする。

(2)被験者への結果説明

被験者から希望があれば、平易な表現で本試験結果をまとめた報告書を作成し、希望する被験者には本試験の結果を通知する。

(3)Plans for granting public access to the full protocol, participant-level dataset, and statistical code

本プロトコルは、国立精神・神経医療研究センター認知行動療法センターのホームページ上および、Primary Outcome Paper が公表される学術雑誌の Website に掲載する予定である。また、本プロトコルを要約した英語論文は BMJ Open にて公表済である[39]。

データセットおよび統計コードの公表は、2021 年 2 月 26 日現在は予定していない。しかし、Primary Outcome Paper が公表される学術雑誌の規定または推奨に従い、しかるべき公開場所があると判断された場合、本試験の共同研究者と協議の上で公開を判断する。

13. Method of Dissemination of Research Information

Dissemination Policy

Plans to communicate trial results to participants, healthcare professionals, and public groups

1. **Public and Academic Disclosure of Results**
   Regardless of the outcomes obtained from this research, the results will be published in academic journals in a manner that ensures participants cannot be identified. The primary outcome paper will be drafted by the principal investigator (Ito), and the draft will be repeatedly reviewed and revised by co-authors until all authors are satisfied. The final version will be published as quickly as possible after the study is completed, considering authorship considerations mentioned in the next section. Other papers based on secondary analyses will be published after the primary outcome paper has been made public.
2. **Explanation of Results to Participants**
   If participants wish, a report summarizing the results of this trial in plain language will be prepared and provided to those participants who request it.
3. **Plans for Granting Public Access to the Full Protocol, Participant-Level Dataset, and Statistical Code**
   This protocol is planned to be published on the website of the Cognitive Behavioral Therapy Center of the National Center of Neurology and Psychiatry, as well as on the website of the academic journal where the primary outcome paper will be published. Additionally, an English summary of

this protocol has already been published in BMJ Open【39】.

As of February 26, 2021, there are no plans to publish the dataset or statistical code. However, in accordance with the regulations or recommendations of the academic journal where the primary outcome paper is published, if it is deemed appropriate to make the data publicly available, this will be decided in consultation with the co-researchers of this trial.

14. 研究対象者等及びその関係者からの相談等への対応

＜研究事務局＞
〒187-8551　東京都小平市小川東町四丁目 1 番 1 号
国立研究開発法人国立精神・神経医療研究センター 認知行動療法センター
電話番号：042-341-271２(内線 3605 または 3606)
対応時間：月・水・金（9 : 30−17 : 00）
主任研究者：認知行動療法センター室長　伊藤正哉
＜苦情等の窓口に関する連絡先＞
〒187-8551　東京都小平市小川東町四丁目 1 番 1 号
国立研究開発国立精神・神経医療研究センター倫理委員会事務局
e-mail:rinri-jimu@ncnp.go.jp

14. Response to Inquiries and Consultations from Research Participants and Their Related Parties

<Research Office>
〒187-8551
4-1-1 Ogawa Higashi-cho, Kodaira City, Tokyo
National Center of Neurology and Psychiatry, Cognitive Behavioral Therapy Center
Phone number: 042-341-2712 (Extension 3605 or 3606)
Office hours: Monday, Wednesday, Friday (9:30 AM - 5:00 PM)
Principal Investigator: Masaya Ito, Director, Cognitive Behavioral Therapy Center

<Contact for Complaints>
〒187-8551
4-1-1 Ogawa Higashi-cho, Kodaira City, Tokyo
Ethics Committee Office, National Center of Neurology and Psychiatry
E-mail: rinri-jimu@ncnp.go.jp

15. 研究対象者等に経済的負担又は謝礼がある場合には、その旨及びその内容

本研究への参加により、通院回数及び検査数が増加するため、研究対象者への費用負担が発生する。

15. Financial Burden or Compensation for Research Participants

Participation in this study may result in increased visits to the clinic and additional tests, which will impose a financial burden on the research participants.

16. 侵襲（軽微な侵襲を除く。）を伴う研究において重篤な有害事象が発生した際の対応

重篤な有害事象とは以下のいずれかの定義に該当する好ましくない事象とする（『人を対象とする医学系研究に関する倫理指針　文部科学省　厚生労働省　平成 26 年』に準じる）。

1) 死に至るもの
2) 生命を脅かすもの
3) 治療のための入院又は入院期間の延長が必要となるもの
4) 永続的又は顕著な障害・機能不全に陥るもの子孫に先天異常を来すもの

報告の対象となる有害事象は、試験期間中の全ての重篤な有害事象、試験治療終了（中止）後に CPT に特有の治療内容との関連性が強く疑われる重篤な有害事象とする。重篤な有害事象が発生した場合、『国立研究開発法人 国立精神・神経医療研究センター 人を対象とする医学系研究に関する業務手順書 第 1 版 平成 27 年 4 月 1 日』に基づき対応する。同手順書では、重篤な有害事象が発生した場合には、倫理委員会作成の報告システムを用いて、速やかに理事長への報告するよう記されている。重篤な有害事象が発生しても、直ちに試験を中止することはなく、各事例の治療継続可否については臨床的意義を最優先して判断する。

16. Response to Severe Adverse Events in Research Involving Invasiveness (excluding minor invasiveness)

Severe adverse events are defined as undesirable events that fall into one of the following categories, according to the "Ethical Guidelines for Medical Research Involving Human Subjects" (Ministry of Education, Culture, Sports, Science and Technology, Ministry of Health, Labour and Welfare, 2014):

1. Fatal
2. Life-threatening
3. Requiring hospitalization or an extension of hospitalization for treatment
4. Resulting in persistent or significant disability or dysfunction
5. Causing congenital abnormalities in offspring

Adverse events that need to be reported include all severe adverse events occurring during the study period and severe adverse events suspected to be strongly related to CPT (Cognitive Processing

Therapy) after the end (or termination) of the trial. In the event of a severe adverse event, the response should follow the "Operational Manual for Medical Research Involving Human Subjects, Version 1, April 1, 2015" by the National Center of Neurology and Psychiatry. This manual states that severe adverse events must be reported to the President promptly using the reporting system created by the ethics committee. Even if a severe adverse event occurs, the study will not be immediately terminated; rather, the continuation of treatment for each case will be determined based on clinical significance as a priority.

17. 当該研究によって生じた健康被害に対する補償の有無及びその内容

健康被害への補償

本試験参加終了後においても、本試験に関する疑問等を被験者が抱いた場合には、研究事務局への連絡ができるものとする。万が一、本研究の介入期間中に健康被害が生じた場合、適切な医療等の対応が行われるように図る。本研究では、研究に伴う健康被害のリスクは、一般の外来診療に伴うもの以上は特にないものと考えられるため、健康被害に対する医療も通常の診療と同様に、被験者の保険診療内で行う。なお、この本研究への参加に起因した健康被害が生じた場合、補償金、医療費・医療手当等の補償は行われないことを、あらかじめ被験者から同意を得るものとする。また、脳画像データの測定に関しては、適切な医療等が行われるように図る。

17. Compensation for Health Damage Caused by the Study and Its Details

Compensation for Health Damage:

After the completion of participation in this study, participants should be able to contact the research office if they have any questions or concerns regarding the study. In the unlikely event that health damage occurs during the intervention period of the study, appropriate medical care will be arranged. Since the risks associated with health damage in this study are considered to be no greater than those associated with routine outpatient care, medical care for health damage will be provided within the participant's insurance coverage, similar to regular medical care. It should be noted that if health damage occurs as a result of participation in this study, compensation in the form of monetary compensation, medical expenses, or medical allowances will not be provided. Participants will be required to give their consent to this in advance. Additionally, appropriate medical measures will be taken in relation to the measurement of brain imaging data.

18. 研究対象者への研究実施後における医療の提供に関する対応

本試験参加終了後においても、本試験に関する疑問等を被験者が抱いた場合には、研究事務局への連絡ができるものとする。

18. Provision of Medical Care to Study Participants After Study Completion

After the completion of participation in this study, if participants have any questions or concerns regarding the study, they should be able to contact the research office.

19.　研究対象者に係る研究結果（偶発的所見を含む。）の取扱い

脳画像データ測定によって偶発的所見が得られた場合の対応

　あらかじめ被験者には、脳画像データの測定によって、腫瘍や脳血管障害等、あるいは他の疾病などの偶発所見が発見される場合があることを本人に了承していただいた上で MRI 撮像を行う。本研究での MRI 撮像方法は診断が目的でないため、臨床的な診断精度はないことや、あくまで通知の内容は診断ではなく腫瘍等が発見される可能性があることを示しており、その後の専門的な検査等で異常がない可能性もあることを、事前に被験者に確認し同意をいただく。そして、偶発所見が発見された場合には、必要に応じ、センター内の専門家（神経内科・放射線科など）に一応のコンサルトをした上で、被験者に偶発所見の内容を通知する。そして、必要に応じて適切な医療機関の受診を勧めるが、その後に診療を受けるかどうかは参加者の選択と責任にゆだねられ、その後の検査等による結果の如何については一切の責任を負えないこと、その後の専門家での診療にかかる諸費用に対しての補償は行えないことを、あらかじめ了承していただいた上で同意を得るものとする。

19. Handling of Research Results (Including Incidental Findings) Related to Study Participants

Response to Incidental Findings from Brain Imaging Data:

Before conducting MRI imaging, participants will be informed and consent to the possibility that incidental findings, such as tumors, cerebrovascular disorders, or other diseases, may be discovered through brain imaging. Since the MRI imaging in this study is not intended for diagnostic purposes, it does not have clinical diagnostic accuracy. Participants will be informed that the notification is not a diagnosis but indicates the possibility of finding tumors or other abnormalities, and that there may be no abnormalities upon further specialized examination. Participants must provide their consent after being fully informed.

If incidental findings are discovered, participants will be notified of the findings after consultation with specialists within the center (such as neurology or radiology) as needed. Participants will be advised to seek appropriate medical care if necessary, but the decision to receive further medical care will be left to the participant's choice and responsibility. The study will not assume any responsibility for the results of further examinations or cover any costs related to subsequent specialist care. Participants must provide their consent with this understanding in advance.

20.　委託する業務内容及び委託先の監督方法

該当なし

20. Outsourced Tasks and Supervision Methods

Not applicable

21. 試料及び情報の二次利用とそれに伴う他機関への提供の可能性

個人が特定できない形式で保存された本研究データのうち、論文公表後に、公共的データベースに登録された場合や他の研究者等の専門家および機関あるいは委員会から要請を受けた場合等があれば、その一部のデータについては本研究以外の研究に使用される可能性がある。ここで想定される二次利用としては、論文の正確性を確認するための追試的な解析結果の報告や、メタアナリシス等のさまざまな研究結果をまとめるための研究が想定される。その他の、本研究の目的以外のデータの二次利用を行う場合には、その時点での最新版の倫理指針に則った研究として倫理審査委員会の承認があることを前提とする。

21. Secondary Use of Samples and Information and the Possibility of Providing Them to Other Institutions

The data from this study, stored in a form that does not identify individuals, may be used in other research if, after publication of the study, the data is registered in public databases or if requests are received from other researchers, experts, institutions, or committees. Potential secondary uses include follow-up analyses to verify the accuracy of the published results or research that aggregates various study results, such as meta-analyses. Any secondary use of data for purposes other than those of this study will require approval from the ethics review committee in accordance with the most current ethical guidelines at that time.

22. モニタリング及び監査

（1）モニタリング

モニタリングの実施にあたっては別途定めるモニタリング計画書に従って行う。

（2）監査

本試験についての監査は予定していない。

22. Monitoring and Auditing

(1) **Monitoring** Monitoring will be conducted according to a separately established monitoring plan.

(2) **Auditing** No audits are planned for this study.

23. 用語の解説

該当なし

23. Glossary of Terms

Not applicable

24. 参考文献リスト、研究に関係する指針・ガイドライン

24. Reference List and Relevant Guidelines

1. Weathers FW, Litz BT, Keane TM, Palmieri PA, Marx BP, Schnurr PP. The PTSD Checklist for DSM-5 (PCL-5). 2013.
2. Jonas DE, Cusack K, Forneris CA, Wilkins TM, Sonis J, Middleton JC, et al. AHRQ Comparative Effectiveness Reviews. Psychological and Pharmacological Treatments for Adults With Posttraumatic Stress Disorder (PTSD). Rockville (MD): Agency for Healthcare Research and Quality (US); 2013.
3. Resick PA, Galovski TE, O'Brien Uhlmansiek M, Scher CD, Clum GA, Young-Xu Y. A randomized clinical trial to dismantle components of cognitive processing therapy for posttraumatic stress disorder in female victims of interpersonal violence. Journal of consulting and clinical psychology. 2008 Apr;76(2):243-58.
4. Resick PA, Monson CM, Chard KM. Cognitiveprocessingtherapy:Veteran/militaryversion:Therapist’smanual. Washington, DC:: Department of Veterans Affairs.; 2014.
5. Resick PA, Monson CM, Chard KM. Cognitive processing therapy: Veteran/military version: Therapist and patient materials manual. Washington, DC: Department of Veterans Affairs.; 2014.
6. Japanese Society for Traumatic Stress Studies. Pharmacotherapy of PTSD guideline for primary care; 1st edition. 2013.
7. Loerinc AG, Meuret AE, Twohig MP, Rosenfield D, Bluett EJ, Craske MG. Response rates for CBT for anxiety disorders: Need for standardized criteria. Clinical psychology review. 2015 Aug 14;42:72-82.
8. Prigerson HG, Frank E, Kasl SV, Reynolds CF, 3rd, Anderson B, Zubenko GS, et al. Complicated grief and bereavement-related depression as distinct disorders: preliminary empirical validation in elderly bereaved spouses. Am J Psychiatry. 1995 Jan;152(1):22-30.
9. Weathers FW, Blake DD, Schnurr PP, Kaloupek DG, Marx BP, Keane TM. The Clinician-Administered PTSD Scale for DSM-5 (CAPS-5). 2013.
10. Sheehan DV, Lecrubier Y, Sheehan KH, Amorim P, Janavs J, Weiller E, et al. The Mini-International Neuropsychiatric Interview (M.I.N.I.): the development and validation of a structured diagnostic psychiatric interview for DSM-IV and ICD-10. The Journal of clinical psychiatry. 1998;59 Suppl 20:22–33;quiz 34-57.
11. Otsubo T, Tanaka K, Koda R, Shinoda J, Sano N, Tanaka S, et al. Reliability and validity of Japanese version of the Mini-International Neuropsychiatric Interview. Psychiatry and clinical neurosciences. 2005 Oct;59(5):517-26.

12. Barlow DH, Gorman JM, Shear MK, Woods SW. Cognitive-behavioral therapy, imipramine, or their combination for panic disorder: A randomized controlled trial. Jama. 2000;283(19):2529-36.

13. Roll D, Ray SE, Marcus SM, Passarelli V, Money R, Barlow DH, et al. Independent evaluator knowledge of treatment in a multicenter comparative treatment study of panic disorder. Neuropsychopharmacology : official publication of the American College of Neuropsychopharmacology. 2004 Mar;29(3):612-8.

14. Bang H, Ni L, Davis CE. Assessment of blinding in clinical trials. Controlled clinical trials. 2004 Apr;25(2):143-56.

15. Prins A, Bovin MJ, Smolenski DJ, Marx BP, Kimerling R, Jenkins-Guarnieri MA, et al. The Primary Care PTSD Screen for DSM-5 (PC-PTSD-5): Development and Evaluation Within a Veteran Primary Care Sample. J Gen Intern Med. 2016 Oct;31(10):1206-11.

16. Kroenke K, Spitzer RL. The PHQ-9: a new depression diagnostic and severity measure. Psychiatric Annals. 2002;32(9):1-7.

17. Muramatsu K, Miyaoka H, Kamijima K, Muramatsu Y, Yoshida M, Otsubo T, et al. The patient health questionnaire, Japanese version: validity according to the mini-international neuropsychiatric interview-plus. Psychol Rep. 2007 Dec;101(3 Pt 1):952-60.

18. Inagaki M, Ohtsuki T, Yonemoto N, Kawashima Y, Saitoh A, Oikawa Y, et al. Validity of the Patient Health Questionnaire (PHQ)-9 and PHQ-2 in general internal medicine primary care at a Japanese rural hospital: a cross-sectional study. General hospital psychiatry. 2013 Nov-Dec;35(6):592-7.

19. Inoue T, Tanaka T, Nakagawa S, Nakato Y, Kameyama R, Boku S, et al. Utility and limitations of PHQ-9 in a clinic specializing in psychiatric care. BMC Psychiatry. 2012;12(1):73.

20. Suzuki K, Kumei S, Ohhira M, Nozu T, Okumura T. Screening for Major Depressive Disorder with the Patient Health Questionnaire (PHQ-9 and PHQ-2) in an Outpatient Clinic Staffed by Primary Care Physicians in Japan: A Case Control Study. PloS one. 2015 03/19
10/30/received
01/09/accepted;10(3):e0119147.

21. Batterham PJ, Ftanou M, Pirkis J, Brewer JL, Mackinnon AJ, Beautrais A, et al. A systematic review and evaluation of measures for suicidal ideation and behaviors in population-based research. Psychol Assess. 2015 Jun;27(2):501-12.

22. EuroQol 開発委員会 日. 日本語版 Euro-Qol の開発. 医療と社会. 1998 1998;8:109-23.

23. Rabin R, Charro Fd. EQ-SD: a measure of health status from the EuroQol Group. Annals of medicine. 2001;33(5):337–43.

24. Shiroiwa T, Fukuda T, Ikeda S, Igarashi A, Noto S, Saito S, et al. Japanese population norms for preference-based measures: EQ-5D-3L, EQ-5D-5L, and SF-6D. Quality of life research : an international journal of quality of life aspects of treatment, care and rehabilitation. 2015 Aug 25.

25. Ikeda S, Shiroiwa T, Igarashi A, Noto S, Fukuda T, Saito S, et al. Developing a Japanese version of the EQ-5D-5L value set. Journal of the National Institute of Public Health. 2015 2015/02;64:47-55.

26. Sheehan DV, Harnett-Sheehan K, Raj BA. The measurement of disability. International clinical psychopharmacology. 1996 Jun;11 Suppl 3:89-95.

27. 吉田卓史. Sheehan Disability Scale (SDISS) 日本語版の作成と信頼性および妥当性の検討. 臨床精神薬理. 2004 2004;7:1645-53.
28. Vogt DS, Shipherd JC, Resick PA. Posttraumatic maladaptive beliefs scale: evolution of the personal beliefs and reactions scale. Assessment. 2012 Sep;19(3):308-17.
29. Kubany ES, Haynes SN, Abueg FR, Manke FP, Brennan JM, Stahura C. Development and validation of the Trauma-Related Guilt Inventory (TRGI). Psychological Assessment. 1996;8(4):428.
30. Pugh LR, Taylor PJ, Berry K. The role of guilt in the development of post-traumatic stress disorder: A systematic review. Journal of affective disorders. 2015 Aug 15;182:138-50.
31. Browne KC, Trim RS, Myers US, Norman SB. Trauma-related guilt: conceptual development and relationship with posttraumatic stress and depressive symptoms. Journal of traumatic stress. 2015 Apr;28(2):134-41.
32. Gross JJ, John OP. Individual differences in two emotion regulation processes: implications for affect, relationships, and well-being. Journal of Personality and Social Psychology. 2003;85(2):348-62.
33. Yoshizu J, Sekiguchi R, Amemiya T. Development of a Japanese version of Emotion Regulation Questionnaire. Japanese Journal of Research on Emotions. 2013 2013;20(2):56-62.
34. Duncan BL, Miller S, Sparks JA, Claud DA, Reynolds LR, Brown J, et al. The Session Rating Scale: Preliminary psychometric properties of a "working" alliance measure. Journal of Brief Therapy. 2003;3(1):3-12.
35. Ministry of Education C, Sports, Science and Technology,, Ministry of Health LaW. Ethical Guidelines for Medical and Health Research Involving Human Subjects. 2014.
36. Ministry of Education C, Sports, Science and Technology, Ministry of Health LaW. Guidance; Ethical Guidelines for Medical and Health Research Involving Human Subjects. 2015.
37. US Department of Health and Human Services. Common Terminology Criteria for Adverse Events (CTCAE) Version 4.0. In: National Insititutes of Health NCI, editor. 2009.
38. Japan Clinical Oncology Group. Common Terminology Criteria for Adverse Events (CTCAE) Version 4.0. 2009.
39. Ito M, Horikoshi M, Resick PA, Katayanagi A, Miyamae M, Takagishi Y, et al. Study protocol for a randomised controlled trial of cognitive processing therapy for post-traumatic stress disorder among Japanese patients: the Safety, Power, Intimacy, Esteem, Trust (SPINET) study. BMJ Open. 2017;7(6).
