# Supplementary material for: Cognitive Processing Therapy for Posttraumatic Stress Disorder in Japan: A Randomized Clinical Trial
Source: JAMA Netw Open. 2025 Feb 5;8(2):e2458059. doi: 10.1001/jamanetworkopen.2024.58059 (PMC11800015; doi:10.1001/jamanetworkopen.2024.58059)
Supplement: Supplement 2. — eMethods. Sample Size Estimation eTable 1. Baseline Trauma Characteristics eResults. Blinding Success for Random Allocation eTable 2. Secondary Outcomes (Dichotomous Variables) eTable 3. Sensitivity Analyses Using a Stratified Variable as a Covariate eTable 4. Sensitivity Analyses Among the Per-Protocol Sample eTable 5. Differences Between the 17- and 34-Week Time Points in the CPT-TAU Group eTable 6. Summary of Independent Evaluator-Rated Adverse Events [file jamanetwopen-e2458059-s002.pdf]

Supplementary Online Content

Ito M, Katayanagi A, Miyamae M, et al. Cognitive processing therapy for posttraumatic stress disorder in Japan: a randomized clinical trial. *JAMA Netw Open*. 2025;8(2):e2458059. doi:10.1001/jamanetworkopen.2024.58059

eMethods. Sample Size Estimation
eTable 1. Baseline Trauma Characteristics
eResults. Blinding Success for Random Allocation
eTable 2. Secondary Outcomes (Dichotomous Variables)
eTable 3. Sensitivity Analyses Using a Stratified Variable as a Covariate
eTable 4. Sensitivity Analyses Among the Per-Protocol Sample
eTable 5. Differences Between the 17- and 34-Week Time Points in the CPT-TAU Group
eTable 6. Summary of Independent Evaluator-Rated Adverse Events

This supplementary material has been provided by the authors to give readers additional information about their work.

© 2025 Ito M et al. *JAMA Network Open*.

eMethods. Sample Size Estimation

Meta-analyses have reported that the effect size in Hedges' g was 1.40 (95% CI 0.85 to 1.95, k = 4, n = 299) for CPT versus control groups (e.g., TAU, waitlist) for post-intervention CAPS scores 72. We planned an LMM to test the primary hypothesis (i.e., that the combination of CPT and TAU would be superior to TAU only at the 17-week assessment). According to Diggle's formula for estimating the LMM sample size 73, a minimal sample size of between 3–24 participants in each group is required when assuming the following: alpha = 0.05 (two-sided); power = 0.9; standardized mean difference = 1.40 (95% CI 0.85 to 1.95); three assessment time points (pre, middle, post); autoregressive covariance structure and within-subjects correlation of 0.2–0.8. Considering the predicted proportion of drop-outs of 22% 25, we selected a conservative sample size of 29 for each arm.

© 2025 Ito M et al. *JAMA Network Open*.

eTable 1. Baseline Trauma Characteristics

	Total (*N* = 60)		CPT-TAU (*N* = 29)		WL-TAU (*N* = 31)	
**Index event**						
Natural disaster	2	(3.3)	2	(6.9)	0	(0.0)
Transportation accident	4	(6.7)	1	(3.4)	3	(9.7)
Physical assault	17	(28.3)	8	(27.6)	9	(29.0)
Assault with a weapon	4	(6.7)	2	(6.9)	2	(6.5)
Sexual assault (e.g., rape, attempted rape, made to perform any type of sexual act through force or threat of harm)	17	(28.3)	8	(27.6)	9	(29.0)
Other unwanted or uncomfortable sexual experience	8	(13.3)	5	(17.2)	3	(9.7)
Life-threatening illness or injury	1	(1.7)	0	(0.0)	1	(3.2)
Severe human suffering	1	(1.7)	0	(0.0)	1	(3.2)
Sudden violent death	5	(8.3)	3	(10.3)	2	(6.5)
Any other very stressful event or experience	1	(1.7)	0	(0.0)	1	(3.2)
**Time with PTSD (months)**	146.95	(126.2)	150.55	(134.0)	143.58	(118.4)
**Time since index event (months)**	189.51	(132.9)	204.03	(121.7)	175.93	(141.3)
**How the traumatic events were experienced**						
Happened to me	58	(96.7)	28	(96.6)	30	(96.8)
Witnessed it	29	(48.3)	12	(41.4)	17	(54.8)
Learned about it	23	(38.3)	9	(31.0)	14	(45.2)
Part of my job	4	(6.7)	2	(6.9)	2	(6.5)
**Reported history of childhood abuse**						
Physical abuse	26	(43.3)	13	(44.8)	13	(41.9)
Sexual abuse	14	(23.3)	9	(31.0)	5	(16.1)
Neglect	16	(26.7)	10	(34.5)	6	(19.4)
Psychological abuse	27	(45.0)	14	(48.3)	13	(41.9)

© 2025 Ito M et al. *JAMA Network Open*.

eResults. Blinding Success for Random Allocation

The independent evaluators accurately guessed the treatment assignment in 30 out of 49 cases at 8 weeks (61% correct, four missing), 32 out of 44 cases at 17 weeks (73% correct, six missing), and 32 of 43 cases at 34 weeks (74% correct, four missing). Bang's Index values for CPT-TAU were .167, .455, .417 at 8, 17, and 34 weeks, respectively. Bang's Index values for WL-TAU were .280, .455, and .579 at 8, 17, and 34 weeks, respectively. Although the limit of the one-sided confidence interval did not cross zero at 8 weeks for CPT-TAU and WL-TAU (−.164 and −.036), it crossed zero at 17 weeks (.142 and .142) and 34 weeks (.111 and .271). These results revealed that the blinding procedure was successful at 8 weeks, but failed at 17 weeks and 34 weeks.

© 2025 Ito M et al. *JAMA Network Open*.

eTable 2. Secondary Outcomes (Dichotomous Variables)

	Incidence proportion				Incidence proportion difference			Incidence proportion ratio		
	CPT-TAU		WL-TAU			95% CI			95% CI	
	Proportion	(SE)	Proportion	(SE)		Lower	Upper		Lower	Upper
Responder status	59.09	10.48	9.09	6.13	50	26.2	73.8	6.5	1.66	25.49
Loss of PTSD diagnosis	63.64	10.26	4.55	4.44	59.09	37.19	81	14	2.01	97.5

CPT-TAU, Cognitive Processing Therapy with Treatment As Usual; WL-TAU, Wait-list with Treatment As Usual

© 2025 Ito M et al. *JAMA Network Open*.

eTable 3. Sensitivity Analyses Using a Stratified Variable as a Covariate

		95% CI	
	**Estimate**	**Lower**	**Upper**
**Primary outcome measure**			
**CAPS-5**	13.85	8.42	19.27
**Secondary outcome measures**			
**PCL-5**	25.23	16.20	34.26
**PHQ-9**	8.89	6.03	11.75
**SIDAS**	6.92	1.38	12.46
**EQ-5D-5L**	-0.15	-0.22	-0.07
**SDS**	8.24	3.94	12.55
**CGI-S**	0.83	0.40	1.26
**CGI-I**	-1.26	-1.76	-0.76

CAPS-5, Clinician-Administered PTSD Scale for DSM-5; PCL-5, Post-traumatic Stress Disorder Checklist for DSM-5; PHQ-9, Patient Health Questionnaire-9; SIDAS, Suicidal Ideation Attributes Scale, EQ-5D-5L, Euro-Qol Five Dimension Five Level; SDS, Sheehan Disability Scale; CGI-S, Clinical Global Impression Severity; CGI-I, Clinical Global Impression Improvement

© 2025 Ito M et al. *JAMA Network Open*.

eTable 4. Sensitivity Analyses Among the Per Protocol Sample

		95% CI	
	**Estimate**	**Lower**	**Upper**
**Primary outcome measure**			
**CAPS-5**	13.86	8.39	19.33
**Secondary outcome measures**			
**PCL-5**	25.45	16.34	34.57
**PHQ-9**	8.80	5.92	11.67
**SIDAS**	7.02	1.43	12.62
**EQ-5D-5L**	-0.14	-0.22	-0.07
**SDS**	8.18	3.83	12.52
**CGI-S**	0.81	0.37	1.24
**CGI-I**	-1.26	-1.76	-0.76

CAPS-5, Clinician-Administered PTSD Scale for DSM-5; PCL-5, Post-traumatic Stress Disorder Checklist for DSM-5; PHQ-9, Patient Health Questionnaire-9; SIDAS, Suicidal Ideation Attributes Scale, EQ-5D-5L, Euro-Qol Five Dimension Five Level; SDS, Sheehan Disability Scale; CGI-S, Clinical Global Impression Severity; CGI-I, Clinical Global Impression Improvement

© 2025 Ito M et al. *JAMA Network Open*.

eTable 5. Differences Between the 17- and 34-Week Time Points in the CPT-TAU Group

Measure	Estimate	95% CI Lower	95% CI Upper
CAPS-5	2.88	-1.47	7.24
PCL-5	4.62	-1.99	11.23
PHQ-9	0.42	-1.94	2.79
SIDAS	-0.41	-4.39	3.56
EQ-5D-5L	-0.02	-0.07	0.04
SDS	4.05	0.99	7.11
CGI-S	0.17	-0.19	0.52
CGI-I	0.46	0.03	0.89

CAPS-5, Clinician-Administered PTSD Scale for DSM-5; PCL-5, Post-traumatic Stress Disorder Checklist for DSM-5; PHQ-9, Patient Health Questionnaire-9; SIDAS, Suicidal Ideation Attributes Scale, EQ-5D-5L, Euro-Qol Five Dimension Five Level; SDS, Sheehan Disability Scale; CGI-S, Clinical Global Impression Severity; CGI-I, Clinical Global Impression Improvement

© 2025 Ito M et al. *JAMA Network Open*.

eTable 6. Summary of Independent Evaluator-Rated Adverse Events

	During intervention period		During follow-up period	
	**CPT-TAU (n = 29)**	**WL-TAU (*n* = 31)**	**CPT-TAU (n = 29)**	**WL-TAU (*n* = 31)**
	**Total**	**Total**	**Total**	**Total**
# of patients reporting AEs	19	23	14	10
# of AEs reported	62	75	25	16
# of SAEs reported	0	3	1	0
AEs by type				
Psychological	17	31	9	6
Medical	45	44	16	10
**Psychological**				
Increased PTSD	0	1	1	0
Anxiety/agitation	4	6	4	1
Depression/anhedonia	5	7	2	0
Irritability/anger	0	6	1	0
Self-harm	2	1	0	1
Nightmares	1	1	1	1
Suicidal ideation	0	5	0	3
Other	5	4	0	0
**Medical**				
Dry mouth	0	0	0	0
Astriction	1	1	0	1

© 2025 Ito M et al. *JAMA Network Open*.

eTable 6. Summary of independent evaluator-rated adverse events, cont

Vision dysregulation	0	0	0	0
Orthostatic hypotension	1	1	1	0
Sleepiness	1	0	0	0
Fatigue	3	5	2	0
Sleeplessness	7	7	3	2
Lack of appetite	2	1	1	1
Gain or loss of body weight	3	2	1	0
Loss of sexual desire	0	0	0	0
Palpitations	1	1	1	0
Tremor	0	2	0	0
Diaphoresis	0	2	0	1
Headache	4	6	1	1
Dizziness	3	2	0	0
Cold symptoms	3	0	3	1
Throat discomfort	0	0	0	0
Gastrointestinal problems	3	1	2	1
Other	13	13	1	2

Participants allocated to WL-TAU received CPT during follow-up period. The adverse events were reported by both therapist and independent evaluators during 0-17 week for CPT-TAU and 17-34 week for WL-TAU. CPT-TAU, Cognitive Processing Therapy with Treatment As Usual; WL-TAU, Wait-list with Treatment As Usual; AE, Adverse Event; SAE, Serious Adverse Event.

© 2025 Ito M et al. *JAMA Network Open*.
