# Supplementary material for: Cognitive Processing Therapy for Posttraumatic Stress Disorder in Japan: A Randomized Clinical Trial
Source: JAMA Netw Open. 2025 Feb 5;8(2):e2458059. doi: 10.1001/jamanetworkopen.2024.58059 (PMC11800015; doi:10.1001/jamanetworkopen.2024.58059)
Supplement: Supplement 3. — Data Sharing Statement [file jamanetwopen-e2458059-s003.pdf]

Data Sharing Statement

Ito. Cognitive Processing Therapy for Posttraumatic Stress Disorder in Japan. *JAMA Netw Open*. Published February 05, 2025. doi:10.1001/jamanetworkopen.2024.58059

Data
Additional Information: umin.ac.jp/ctr UMIN000021670 (https://center6.umin.ac.jp/cgi-open-bin/ctr/ctr_view.cgi?recptno=R000024285)
Data available: Yes
Data types: Deidentified participant data, Data dictionary
How to access data: When the ethical committee at the National Center of Neurology and Psychiatry permits, the de-identified patient data may be shared upon request to the corresponding author.
When available: With publication

Supporting Documents
Document types: Statistical/analytic code
How to access documents: masayait@ncnp.go.jp
When available: With publication

Additional Information
Who can access the data: Researchers whose proposed use of the data has been approved.
Types of analyses: The data will be made available for any purpose except for commercial use.
Mechanisms of data availability: The data will be available after the proposal is approved by the National Center of Neurology and Psychiatry ethical committee.
